# Effects of Posttrial Antihypertensive Drugs on Morbidity and Mortality: Findings from 15-Year Passive Follow-Up after ALLHAT Ended

**DOI:** 10.1155/2021/2261144

**Published:** 2021-12-09

**Authors:** Xianglin L. Du, Lara M. Simpson, Brian C. Tandy, Judy Bettencourt, Barry R. Davis

**Affiliations:** ^1^Department of Epidemiology, Human Genetics and Environmental Sciences, School of Public Health, The University of Texas Health Science Center at Houston, 1200 Pressler St, Houston, TX 77030, USA; ^2^Coordinating Center for Clinical Trials, Department of Biostatistics and Data Science, School of Public Health, The University of Texas Health Science Center at Houston, 1200 Pressler St, Houston, TX 77030, USA

## Abstract

**Background:**

Antihypertensive and Lipid-Lowering Treatment to Prevent Heart Attack Trial (ALLHAT) ended in 2002, but it is important to study its long-term outcomes during the posttrial period by incorporating posttrial antihypertensive medication uses in the analysis.

**Purposes:**

The primary aim is to explore the patterns of antihypertensive medication use during the posttrial period from Medicare Part-D data over the 11-year period from 2007 to 2017. The secondary aim is to examine the potential effects of these posttrial antihypertensive medications on the observed mortality and morbidity benefits.

**Methods:**

This is a posttrial passive follow-up study of ALLHAT participants in 567 US centers in 1994–1998 with the last date of active in-trial follow-up on March 31, 2002, by linking with their Medicare and National Death Index data through 2017 among 8,007 subjects receiving antihypertensive drugs (3,637 for chlorthalidone, 2,189 for amlodipine, and 2,181 for lisinopril). Outcomes included posttrial antihypertensive drug use, all-cause mortality, and cardiovascular disease (CVD) mortality.

**Results:**

Of 8007 subjects, 3,637 participants were initially randomized to diuretic (chlorthalidone). The majority (67.9%) of them still received diuretics in 2007, and 52.7%, 47.2%, and 44.0% received *β*-blockers, angiotensin-converting enzyme (ACE) inhibitors, and calcium channel blockers (CCBs), respectively. Compared to participants who received diuretic-based antihypertensives, those who received CCB had a nonsignificantly higher risk of all-cause mortality (1.17, 0.99–1.37), whereas those who received ACE/ARB (angiotensin receptor blockers) had a significantly higher risk of all-cause mortality (1.26, 1.09–1.45). For the combined fatal or nonfatal hospitalized events, the risk of CVD was significantly higher in patients receiving CCB (1.30, 1.04–1.61) and ACE/ARB (1.49, 1.22–1.81) as compared to patients receiving diuretics.

**Conclusion:**

After the conclusion of the ALLHAT, almost all patients switched to combination antihypertensive therapies, independently by the original drug class, and the combination therapies (mostly based on diuretics) reduced the incidence of major cardiovascular outcomes and mortality.

## 1. Introduction

The Antihypertensive and Lipid-Lowering Treatment to Prevent Heart Attack Trial (ALLHAT) was a multicenter, randomized, double-blind, active-controlled trial conducted in a total of 42,418 participants aged 55 years or older with hypertension and at least 1 other coronary heart disease (CHD) risk factor in 623 North American centers. The ALLHAT's recruitment period was from February 1, 1994, to January 31, 1998 with the last date of active in-trial follow-up on March 31, 2002. This trial addressed the question of comparative efficacy of newer classes of antihypertensive agents with the standard diuretic treatment in preventing cardiovascular disease (CVD) outcomes. As a large community-based, randomized, controlled clinical trial, ALLHAT has demonstrated the relative efficacy of different antihypertensive drug classes overall and in some major subgroups [1–13]. These results have been translated into clinical practice guidance [14–17]. Major results from the antihypertensive component of the trial (AHT) demonstrated that neither amlodipine nor lisinopril nor doxazosin were superior to chlorthalidone in preventing most types of CVD [1, 2], and in fact, chlorthalidone was superior to all 3 of those drugs in preventing heart failure (HF) [1–4]. Those analyses formed a major part of the JNC-7 (The Seventh Report of the Joint National Committee on Prevention, Detection, Evaluation, and Treatment of High Blood Pressure) guidelines published in 2003 [14].

Posttrial follow-up studies used the ALLHAT data that were linked with Medicare data in 2002–2006 [18–25], but none of those previous studies incorporated posttrial medication use and no information on the posttrial antihypertensive medications was available at that time. Although these studies have made significant contributions to our understanding of long-term legacy effects of ALLHAT medications, it would be ideal to consider the impact of medication use during the posttrial period on the long-term study outcomes. The federal Medicare insurance program, as the primary insurance for people aged 65 or older, implemented its Part-D comprehensive drug coverage in 2006 for the first time in Medicare history, making it possible now to monitor the use of these antihypertensive medications during the posttrial period. Therefore, we requested the data of ALLHAT participants to be linked with their Medicare Claims and Part-D drug data through December 2017. The primary aim of this study is to explore the patterns (frequencies and types of drugs) of antihypertensive medication use during the posttrial period from Medicare Part-D data in those eligible patients over the 11-year period from 2007 to 2017. Our secondary aim is to examine the potential effects of these posttrial antihypertensive medications on the observed mortality and morbidity benefits associated with the initial 3 trial treatments (diuretic, angiotensin-converting enzyme (ACE) inhibitor, and calcium channel blocker (CCB)).

## 2. Methods

### 2.1. Study Population and Data Sources

The detailed methods of ALLHAT have been reported previously [1, 2, 4, 19]. In brief, 42,418 participants who were eligible and who agreed to participate in the ALLHAT were randomly assigned to 4 treatment arms: an angiotensin-converting enzyme (ACE) inhibitor (lisinopril) (*n* = 9,054), CCB (amlodipine) (*n* = 9,048), *α*-blocker (doxazosin) (*n* = 9,061), or a thiazide-type diuretic (chlorthalidone) (*n* = 15,255). Average follow-up was 4.9 years (ranging from 4 to 8 years) in all arms except for doxazosin-chlorthalidone comparison which was terminated early with a mean follow-up of 3.2 years due to a higher incidence of CVD events. Therefore, this study did not include those patients on doxazosin. The data of ALLHAT participants have been linked with their Medicare Claims from 1994 to 2017, Part-D comprehensive pharmacy data from 2007 to 2017, and National Death Index data from 1994 to 2017.

**Figure 1:**
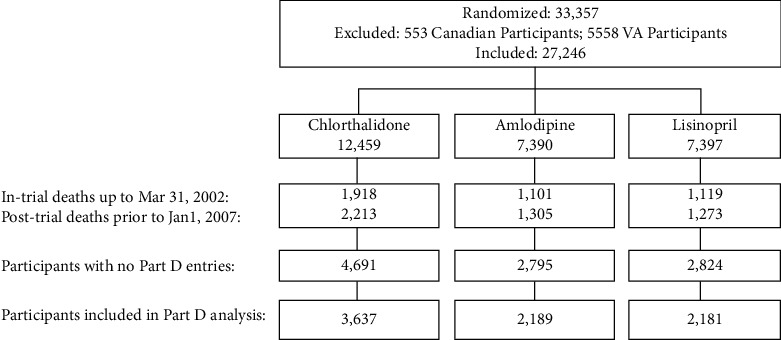
Study participant inclusion and exclusion and number of subjects in the final analysis.

Study participants, exclusions, and number of subjects in the final analysis are illustrated in Figure 1. After excluding those who were not eligible for Medicare, deaths, and no Medicare Part-D enrollment by January 1, 2007, 8,007 subjects (3,637 for chlorthalidone, 2,189 for amlodipine, and 2,181 for lisinopril) in 567 US centers were included in the final analysis. The Committee for the Protection of Human Subjects at the University of Texas Health Science Center in Houston approved this study.

### 2.2. Study Variables

The key variable was to identify what antihypertensive drugs during the posttrial periods in 2007–2017 were received by ALLHAT participants. These posttrial antihypertensive drugs were identified from Medicare Part-D data through brand or generic drug names or the National Drug Codes of antihypertensive drugs, which were then classified into 13 major categories (see Supplementary Table S1). The frequency and patterns of posttrial antihypertensive drug utilization and their changes over time from 2007 to 2017 were presented.

Mortality outcomes were identified through the previously well-validated algorithms that utilized the data from the ALLHAT, the Social Security Administration (SSA), and National Death Index (NDI) [19]. The in-trial deaths in 1994–2002 were ascertained by investigators and were confirmed by death certificates, and the posttrial deaths in 2002–2017 were ascertained from the SSA and NDI. Morbidity variables included the incidence of combined fatal and nonfatal hospitalized events (CVD, CHD, HF, stroke, cancer, kidney disease, or end-stage renal disease). During the posttrial period, nonfatal hospitalized events were ascertained from Medicare inpatient data for hospitalized patients and predefined ICD-9 or ICD-10 codes (see Supplementary Tables S2a–S2e) that have been validated for each mortality and morbidity outcome of interest.

### 2.3. Statistical Analysis

Among the study comparison groups that were arms of ALLHAT, the receipt and percentage of those receiving various categories of antihypertensive drugs according to Part-D data in 2007 are presented. The changing patterns and percentage of those receiving antihypertensive drugs from 2008 to 2017 are also presented among the initial 3 ALLHAT study arms. Patients were then classified into 2 groups: one group continued receiving the same class of study drugs and the other group did not receive the same class of study drugs. We determined if these patients received the study drugs plus additional 1, 2, 3, or more antihypertensive drugs or no study drugs but received 1, 2, 3, or more other classes of antihypertensive drugs. The number and cumulative incidence (probability) of having the mortality and morbidity outcomes over the 11-year period from 2007 to 2017 are presented. Landmark analyses using time to event Cox regression analyses (hazard ratio and 95% confidence intervals) for mortality and morbidity outcomes over the 11-year period from 2007 to 2017 are presented and adjusted for potential confounding factors.

## 3. Results

Although the randomization was no longer intact, distribution of characteristics in those eligible for the follow-up observations was comparable among the 3 groups of the initial trial (see Supplementary Table S3).

Table 1 presents the receipt of antihypertensive drugs in 2007 stratified by the 3 initial trial groups. Among those 3,637 participants assigned to diuretic (chlorthalidone), the majority (67.9%) of them still received diuretic-based medications (i.e., any drugs under the category of diuretics as listed in Supplementary Table S1) in 2007 and 52.7%, 47.2%, and 44.0% received *β*-blockers, ACE inhibitors (any drugs under the category of ACEs as listed in Supplementary Table S1), and CCBs (any drugs under the category of CCBs as listed in Supplementary Table S1), respectively. Among those 2,189 participants assigned to CCB (amlodipine), the majority (62.6%) of them received diuretic-based medications in 2007, followed by CCB (50.7%), *β*-blockers (50.2%), and ACE inhibitors (46.8%). Similarly, in those 2,181 participants assigned to ACE inhibitors (lisinopril), the majority (63.5%) of them now received diuretic-based medications in 2007, followed by ACE inhibitors (51.8%), *β*-blockers (51.4%), and CCB (44.4%). Table 1 also presents the changing utilization of antihypertensive drugs from 2008 to 2017, but the general patterns of receiving the classes of antihypertensive drugs were similar to those of 2007.

Table 2 presents the patterns of receiving multiple combinations of posttrial antihypertensive drugs by 3 initial study arms. In the thiazide-type diuretic arm, 44.3% still used the same type of diuretics, 49.7% did not receive thiazide-type diuretics but received other diuretics (such as loop) or other classes of antihypertensive drugs, and 6.0% did not receive any class of antihypertensive drugs as listed in the table in 2007. In those who used diuretic-based medications (44.3%), only 2.3% received diuretics alone without other classes of antihypertensive drugs, but 10.1%, 14.8%, and 17.2%, respectively, received diuretics with an additional 1, 2, and 3 or more other classes of antihypertensive drugs. In those who did not receive thiazide-type diuretics, 11.4%, 15.4%, and 22.8%, respectively, received 1, 2, and 3 or more other classes of antihypertensive drugs, most of which were ACE/ARBs, *β *-blockers, CCB, or loop diuretics. In the CCB arm, 50.7% continued to receive CCBs (of which, 4.2% received CCB alone and 46.5% received CCB plus other classes of antihypertensive drugs), 44.8% did not receive CCBs but received other antihypertensive classes, and 6.1% received no antihypertensive drugs. In the ACE group, 71.6% continued to receive ACE/ARB (of which, 5.8% received ACE/ARB alone and 65.8% received ACE/ARB plus other classes of antihypertensive drugs), 23.0% did not receive ACE/ARB but received other drugs, and 5.5% did not receive any antihypertensive drugs. These patterns of receiving multiple combinations of posttrial antihypertensive drugs were also stratified by race and gender (see Supplementary Tables S4a–S4d).

The cumulative incidences or mortality and morbidity outcomes that occurred from 2007 to 2017 according to whether patients who received the study drug class with an additional 1, 2, 3, or more other classes of antihypertensive drugs by the 3 initial study arms are presented in Supplementary Table S5. The all-cause mortality rate was lower in those patients treated with diuretics plus one (22.4%) or two (22.4%) antihypertensive drug classes (dual or triple combination therapies) than in those treated with diuretic monotherapy (24.4%) and in those treated with diuretics plus 3 or more other classes of antihypertensive drugs (30.2%).

Table 3 presents the effects of antihypertensive drug categories on the risk of various clinical outcomes (all-cause mortality, CVD mortality, and non-CVD mortality such as cancer and kidney disease mortality and fatal and nonfatal hospitalized events) that occurred between 2007 and 2017. For example, among those trial participants originally assigned to chlorthalidone who continued to receive diuretics only, subjects who received diuretics plus 1 to more than 3 additional antihypertensive drug classes did not have significantly different risks of mortality, whereas those who did not continue to receive diuretics but took 3 (adjusted hazard ratio: 1.45, 95% CI: 1.03–2.05) or 4 (1.97, 1.38–2.81) other classes of antihypertensive drugs had statistically significantly higher risks of all-cause mortality after adjusting for confounders. These higher risks were also observed for combined fatal and nonfatal CVD for subjects who received 3 other antihypertensive drugs (1.60, 1.04–2.49) or 4 other antihypertensive drugs (2.44, 1.57–3.81).

Table 4 presents the risk of mortality and morbidity outcomes by the head-to-head comparisons of the 3 groups. Compared to participants who received diuretic-based antihypertensive drugs without CCB or ACE/ARB, those who received CCB drugs had a nonsignificantly higher adjusted risk of all-cause mortality (1.17, 0.99–1.37), whereas those who received ACE/ARB drugs had a significantly higher adjusted risk of all-cause mortality (1.26, 1.09–1.45). The risk of CVD, CHD, other CVD mortality, and kidney disease mortality was elevated as well in those patients who received CCB or ACE/ARB but was mostly not statistically significant, while risks of stroke mortality and HF mortality were not elevated as compared to patients who received diuretic-based antihypertensive drugs without CCB or ACE/ARB. For the combined fatal or nonfatal hospitalized events, the risk of CVD (1.30, 1.04–1.61), HF (1.49, 1.14–1.95), and kidney disease (3.75, 1.82–7.73) was significantly higher in patients receiving CCB and the risk of CVD (1.49, 1.22–1.81), CHD (1.35, 1.00–1.81), HF (1.69, 1.33–2.16), and stroke (1.60, 1.11–2.29) was significantly higher in patients receiving ACE/ARB as each compared to patients receiving diuretic. The risk of CVD and non-CVD mortality was not significantly different between patients receiving CCB and those receiving ACE/ARB (last column in Table 4). The above findings were supported by internal comparisons (diuretic vs no diuretic, CCB vs no CCB, and ACE/ARB vs no ACE/ARB) and by across-group comparisons in those with CCB and ACE/ARB only as compared to those with diuretic only (see Supplementary Tables S6 and Tables S7a–S7b).

## 4. Discussion

This study examined the patterns of antihypertensive medication drugs received during the posttrial period from Medicare Part-D coverage in 2007 and their changing patterns of antihypertensive drugs over the next 10 years from 2008 to 2017. In 2007, 5 years after the in-trial follow-up ended, most subjects still received the same class of antihypertensive drugs which were randomly assigned to them during the initial trial period. Subjects who received diuretic plus 1 to more than 3 additional antihypertensive drugs had an elevated but not significantly different risk of mortality. However, the risk of CHD mortality and other CVD mortality and the risk of combined fatal and nonfatal hospitalized events for CVD, CHD, HF, and kidney disease were significantly elevated in those taking 4 or more antihypertensive drugs in all 3 groups of subjects. The risk of combined fatal and nonfatal hospitalized events for kidney disease or end-stage renal failure was particularly higher in those who took 4 or more drugs with or without ACE/ARB. Those who received CCB and ACE/ARB drugs had a significantly higher adjusted risk of all-cause mortality than participants who received diuretic-based antihypertensive drugs. The risk of CVD and non-CVD mortality was not significantly different between patients receiving CCB and those receiving ACE/ARB. The findings remained unchanged after adjusting for key confounding factors.

Our study linked the data of ALLHAT participants with their Medicare Claims, Part-D drug files, and NDI data through December 2017. Hence, these datasets enabled us to address the changing patterns of posttrial antihypertensive drugs and the impact of these medications on the long-term risks of mortality and morbidity outcomes. The findings from this study added the following unique and novel information to the existing literature. First, to the best of our knowledge, this was the first study to identify the type and frequency of posttrial antihypertensive medication use in ALLHAT participants during the 11-year period in 2007–2017. Second, our study considered the data on antihypertensive medication changes over time in the analyses and was the first one to examine the risk of long-term outcomes that may be associated with antihypertensive drugs during the posttrial period while adjusting for posttrial antihypertensive medication usage. Therefore, the study added new and significant elements to the existing literature in comparing the risk of long-term overall mortality, CVD-specific mortality, and other causes of death in patients receiving diuretics as compared to those receiving ACE or CCB. Furthermore, findings from our study supported the long-term legacy effects of antihypertensive drugs on mortality and morbidity outcomes over additional 15 years of follow-up from the end of in-trial follow-up in 2002 to December 2017. For example, the adjusted risk of all-cause mortality was significantly higher in those who received ACE/ARB drugs than in participants who received diuretic-based antihypertensive drugs in 2007–2017. Similarly, the risk of combined fatal or nonfatal CVD, CHD, HF, stroke, and kidney disease was significantly higher in those receiving CCB and ACE/ARB than in patients receiving diuretics. The risk of CVD, CHD, other CVD mortality, and kidney disease mortality was elevated as well in these patients but was not statistically significant. Our study conclusions were consistent with what were found in the initial ALLHAT results [1, 2] and also supported several studies using posttrial Medicare follow-up data to 2006 [19–22].

Major results from the antihypertensive component of the trial (AHT) demonstrated that neither amlodipine nor lisinopril were superior to chlorthalidone in preventing most types of CHD [1, 2], and in fact, chlorthalidone was superior to those drugs in preventing HF [1, 2, 8]. Those analyses formed a major part of the JNC-7 (The Seventh Report of the Joint National Committee on Prevention, Detection, Evaluation, and Treatment of High Blood Pressure) guidelines published in 2003 [14]. Analyses of the follow-up data to 2006 also confirmed the main trial's original conclusions on total mortality, CHD, CVD, stroke, or end-stage renal disease [19–22]. Cushman and colleagues used the linked ALLHAT data with the NDI and Medicare data and found no significant differences in cardiovascular mortality for amlodipine or lisinopril as compared with chlorthalidone [19]. The only significant differences in secondary outcomes were for HF, which was higher with amlodipine (HR, 1.12; 95% CI, 1.02–1.22), and stroke mortality, which was higher with lisinopril (HR, 1.20; 95% CI, 1.01–1.41), each compared with chlorthalidone [19]. Our study using the NDI and Medicare data through December 2017 linked with ALLHAT participants also showed that those who received ACE/ARB drugs had a significantly higher adjusted risk of all-cause mortality (1.26; 95% CI, 1.09–1.45) than those with diuretics. The all-cause mortality rate appeared to be lower in those patients treated with diuretics plus one (22.4%) or two (22.4%) antihypertensive drug classes (dual or triple combination therapies) than in those treated with diuretic monotherapy (24.4%), although these lower mortality risks were not statistically significant (Table 4). For the combined fatal or nonfatal hospitalized events, the risk of CVD, HF, and kidney disease was significantly higher in patients receiving CCB and the risk of CVD, CHD, HF, and stroke was significantly higher in patients receiving ACE/ARB as each compared to patients receiving diuretics. In addition, we found a higher risk of CHD mortality and other CVD mortality and the risk of combined fatal and nonfatal hospitalized events for CVD, CHD, HF, and kidney disease in those taking 4 or more antihypertensive drugs in all 3 groups of subjects and also found a higher risk of combined fatal and nonfatal hospitalized events for kidney disease or end-stage renal failure in those who took 4 or more drugs with or without ACE/ARB. These results were observed likely because those patients who took more drugs might have poorly controlled blood pressure and their physicians might have tried to prescribe multiple antihypertensive drugs in order to get their blood pressure under control.

Our study has some limitations. First, we included ALLHAT participants who were still alive and enrolled in Medicare Part-D program in 2007. Hence, initial trial randomization was no longer intact, and any analyses done off-randomization may be subject to unmeasured or unknown confounders. In addition, the time of onset, duration, and severity of comorbidities and their changes over time are difficult to identify accurately in Medicare Claims datasets. Although we adjusted for some measured confounders, unmeasured or unknown confounders among the comparison groups may still be present, affecting the study findings and conclusions. Second, posttrial data were not available on blood pressures during the posttrial period; hence, it was not known how well blood pressures were controlled. Even though we did control for final systolic blood pressures at the end of in-trial period, blood pressure levels in more recent years should be helpful. Third, treatment adherence for hypertension, diabetes, and cardiovascular diseases cannot be measured accurately, and their effects on the findings were not known. One study found that about 73.7% of Medicare Part-D beneficiaries in the US using antihypertensives in 2014 were adherent to their regimen [26]. Another study showed this low adherence to antihypertensive medication has decreased among Medicare beneficiaries from 2007 to 2012 [27]. Finally, because Medicare Part-D drug coverage was implemented in 2006, the posttrial antihypertensive medications between 2002 and 2006 were not available for analyses and their effects on the study outcomes cannot be assessed. Due to these limitations, the study findings and conclusions may be interpreted with caution.

In conclusion, after the ALLHAT trial was completed in 2002, almost all patients switched to combination antihypertensive therapies, independently by the original drug class, and the combination therapies (mostly based on diuretics) reduced the incidence of major cardiovascular outcomes and mortality. The risk of CVD and non-CVD mortality was not significantly different between patients receiving CCB and those receiving ACE/ARB. Further study may be needed to compare the long-term legacy effects of antihypertensive drugs on the mortality and morbidity outcomes during the entire in-trial periods that began in 1994 and the passive follow-up periods through 2017 among three comparison groups.

## Figures and Tables

**Table 1:** Patterns of posttrial antihypertensive drug utilization from Medicare Part-D drug coverage as of 2007.

	N (%) receiving antihypertensive druga in 2007–2017 by initial ALLHAT 3 arms			
	Chlorthalidone	Amlodipine	Lisinopril	Total
Antihypertensive drugs in 2007 as baseline from 1^st^ year enrolled in CMS/Part-D data	3420 (94)	2055 (93.9)	2062 (94.5)	7537 (94.1)
Aldosterone antagonists^*∗*^^†^	187 (5.1)	87 (4.0)	101 (4.6)	375 (4.7)
Alpha 1 adrenergic receptor agonist (selective; *α*-blocker)	201 (5.5)	124 (5.7)	128 (5.9)	453 (5.7)
Angiotensin-converting enzyme (ACE) inhibitor	1717 (47.2)	1024 (46.8)	1129 (51.8)	3870 (48.3)
Angiotensin II receptor blockers (ARB)	983 (27.0)	602 (27.5)	592 (27.1)	2177 (27.2)
Total ACE inhibitor + ARB	2443 (67.2)	1457 (66.6)	1562 (71.6)	5462 (68.2)
Arteriolar vasodilators	177 (4.9)	112 (5.1)	153 (7.0)	442 (5.5)
Autonomic ganglionic vasodilators	0 (0)	0 (0)	0 (0)	0 (0)
Beta adrenergic blockers (*β*-blockers)	1915 (52.7)	1099 (50.2)	1121 (51.4)	4135 (51.6)
Calcium channel blocker (CCB)-dihydropyridine	1316 (36.2)	956 (43.7)	804 (36.9)	3076 (38.4)
Calcium channel blocker (CCB)-nondihydropyridine	345 (9.5)	191 (8.7)	193 (8.8)	729 (9.1)
Total CCB-dihydropyridine + nondihydropyridine	1602 (44.0)	1109 (50.7)	968 (44.4)	3679 (45.9)
Central alpha 2 adrenergic agonists	330 (9.1)	185 (8.5)	219 (10.0)	734 (9.2)
Diuretics: thiazide^*∗*^	1305 (35.9)	805 (36.8)	797 (36.5)	2907 (36.3)
Diuretics: thiazide-type^*∗*^	348 (9.6)	98 (4.5)	97 (4.4)	543 (6.8)
Diuretics: thiazide or thiazide-type	1613 (44.3)	890 (40.7)	877 (40.2)	3380 (42.2)
Diuretics: loop^*∗*^	1095 (30.1)	652 (29.8)	682 (31.3)	2429 (30.3)
Diuretics: potassium-sparing^*∗*^^†^	189 (5.2)	90 (4.1)	92 (4.2)	371 (4.6)
Total-all diuretics^*∗*^	2470 (67.9)	1371 (62.6)	1386 (63.5)	5227 (65.3)
Peripheral adrenergic neuron antagonist^†^	6 (0.2)	2 (0.1)	3 (0.1)	11 (0.1)
Renin inhibitors	11 (0.3)	4 (0.2)	4 (0.2)	19 (0.2)
No drugs	217 (6.0)	134 (6.1)	119 (5.5)	470 (5.9)
Total	3637 (100)	2189 (100)	2181 (100)	8007 (100)
Antihypertensive drugs in 2008–2010 (baseline: enrolled CMS/Part-D 2007, continued through 2008–2010)	3214 (97.4)	1939 (97.1)	1920 (97.6)	7073 (97.4)
Aldosterone antagonists^*∗*^^†^	271 (8.2)	144 (7.2)	126 (6.4)	541 (7.4)
Alpha 1 adrenergic receptor agonist (selective; *α*-blocker)	293 (8.9)	171 (8.6)	185 (9.4)	649 (8.9)
Angiotensin-converting enzyme (ACE) inhibitor	1968 (59.7)	1184 (59.3)	1221 (62.1)	4373 (60.2)
Angiotensin II receptor blockers (ARB)	1142 (34.6)	701 (35.1)	679 (34.5)	2522 (34.7)
Total ACE inhibitor + ARB	2569 (77.9)	1570 (78.6)	1587 (80.7)	5726 (78.8)
Arteriolar vasodilators	282 (8.5)	163 (8.2)	202 (10.3)	647 (8.9)
Autonomic ganglionic vasodilators	0 (0)	0 (0)	0 (0)	0 (0)
Beta adrenergic blockers (*β*-blockers)	2093 (63.4)	1224 (61.3)	1237 (62.9)	4554 (62.7)
Calcium channel blocker (CCB)-dihydropyridine	1621 (49.1)	1113 (55.7)	973 (49.5)	3707 (51.0)
Calcium channel blocker (CCB)-nondihydropyridine	384 (11.6)	211 (10.6)	227 (11.5)	822 (11.3)
Total CCB-dihydropyridine + nondihydropyridine	1850 (56.1)	1245 (62.3)	1111 (56.5)	4206 (57.9)
Central alpha 2 adrenergic agonists	395 (12.0)	241 (12.1)	279 (14.2)	915 (12.6)
Diuretics: thiazide^*∗*^	1452 (44.0)	902 (45.2)	895 (45.5)	3249 (44.7)
Diuretics: thiazide-type^*∗*^	410 (12.4)	131 (6.6)	154 (7.8)	695 (9.6)
Diuretics: thiazide or thiazide-type	1757 (53.3)	998 (50.0)	1000 (50.8)	3755 (51.7)
Diuretics: loop^*∗*^	1393 (42.2)	806 (40.4)	806 (41.0)	3005 (41.4)
Diuretics: potassium-sparing^*∗*^^†^	195 (5.9)	102 (5.1)	99 (5.0)	396 (5.5)
Total-all diuretics^*∗*^	2572 (78.0)	1455 (72.9)	1458 (74.1)	5485 (75.5)
Peripheral adrenergic neuron antagonist^†^	7 (0.2)	3 (0.2)	3 (0.2)	13 (0.2)
Renin inhibitors	51 (1.5)	25 (1.3)	23 (1.2)	99 (1.4)
No drugs	85 (2.6)	58 (2.9)	47 (2.4)	190 (2.6)
Total	3299 (100)	1997 (100)	1967 (100)	7263 (100)

Antihypertensive drugs in 2011–2013 (baseline: enrolled CMS/Part-D 2007, continued through 2010 to 2011–2013)	2376 (97.7)	1449 (96.7)	1412 (97.5)	5237 (97.3)
Aldosterone antagonists^*∗*^^†^	192 (7.9)	115 (7.7)	103 (7.1)	410 (7.6)
Alpha 1 adrenergic receptor agonist (selective; *α*-blocker)	207 (8.5)	123 (8.2)	131 (9.0)	461 (8.6)
Angiotensin-converting enzyme (ACE) inhibitor	1298 (53.3)	768 (51.2)	813 (56.1)	2879 (53.5)
Angiotensin II receptor blockers (ARB)	926 (38.1)	537 (35.8)	546 (37.7)	2009 (37.3)
Total ACE inhibitor + ARB	1898 (78.0)	1126 (75.1)	1180 (81.5)	4204 (78.1)
Arteriolar vasodilators	278 (11.4)	160 (10.7)	191 (13.2)	629 (11.7)
Autonomic ganglionic vasodilators	0 (0)	0 (0)	0 (0)	0 (0)
Beta adrenergic blockers (*β*-blockers)	1570 (64.5)	927 (61.8)	934 (64.5)	3431 (63.8)
Calcium channel blocker (CCB)-dihydropyridine	1247 (51.3)	820 (54.7)	767 (53.0)	2834 (52.7)
Calcium channel blocker-nondihydropyridine	249 (10.2)	144 (9.6)	147 (10.2)	540 (10.0)
Total CCB–dihydropyridine + nondihydropyridine	1414 (58.1)	912 (60.8)	869 (60.0)	3195 (59.4)
Central alpha 2 adrenergic agonists	264 (10.9)	159 (10.6)	172 (11.9)	595 (11.1)
Diuretics: thiazide^*∗*^	1005 (41.3)	602 (40.2)	593 (41.0)	2200 (40.9)
Diuretics: thiazide-type^*∗*^	276 (11.3)	103 (6.9)	106 (7.3)	485 (9.0)
Diuretics: thiazide or thiazide-type	1206 (49.6)	672 (44.8)	666 (46.0)	2544 (47.3)
Diuretics: loop^*∗*^	1039 (42.7)	638 (42.6)	602 (41.6)	2279 (42.4)
Diuretics: potassium-sparing^*∗*^^†^	111 (4.6)	67 (4.5)	57 (3.9)	235 (4.4)
Total-all diuretics^*∗*^	1841 (75.7)	1071 (71.4)	1033 (71.3)	3945 (73.3)
Peripheral adrenergic neuron antagonist^†^	2 (0.1)	2 (0.1)	0 (0)	4 (0.1)
Renin inhibitors	36 (1.5)	18 (1.2)	15 (1.0)	69 (1.3)
No drugs	57 (2.3)	50 (3.3)	36 (2.5)	143 (2.7)
Total	2433 (100)	1499 (100)	1448 (100)	5380 (100)

Antihypertensive drugs in 2014–2017 (baseline: enrolled CMS/Part-D 2007, continued through 2013 into 2014–2017)	1728 (97.9)	1038 (97.1)	1017 (97)	3783 (97.4)
Aldosterone antagonists^*∗*^^†^	173 (9.8)	107 (10.0)	96 (9.2)	376 (9.7)
Alpha 1 adrenergic receptor agonist (selective; *α*-blocker)	137 (7.8)	80 (7.5)	93 (8.9)	310 (8.0)
Angiotensin-converting enzyme (ACE) inhibitor	819 (46.4)	487 (45.6)	492 (46.9)	1798 (46.3)
Angiotensin II receptor blockers (ARB)	716 (40.6)	424 (39.7)	432 (41.2)	1572 (40.5)
Total ACE inhibitor + ARB	1349 (76.4)	793 (74.2)	819 (78.1)	2961 (76.3)
Arteriolar vasodilators	248 (14.1)	156 (14.6)	164 (15.6)	568 (14.6)
Autonomic ganglionic vasodilators	0 (0)	0 (0)	0 (0)	0 (0)
Beta adrenergic blockers (*β*-blockers)	1172 (66.4)	706 (66.0)	694 (66.2)	2572 (66.3)
Calcium channel blocker (CCB)-dihydropyridine	960 (54.4)	629 (58.8)	588 (56.1)	2177 (56.1)
Calcium channel blocker (CCB)-nondihydropyridine	189 (10.7)	84 (7.9)	102 (9.7)	375 (9.7)
Total CCB-dihydropyridine + nondihydropyridine	1071 (60.7)	684 (64.0)	654 (62.4)	2409 (62.1)
Central alpha 2 adrenergic agonists	174 (9.9)	107 (10.0)	101 (9.6)	382 (9.8)
Diuretics: thiazide^*∗*^	659 (37.3)	395 (37.0)	389 (37.1)	1443 (37.2)
Diuretics: thiazide-type^*∗*^	195 (11.0)	89 (8.3)	84 (8.0)	368 (9.5)
Diuretics: thiazide or thiazide-type	804 (45.6)	456 (42.7)	444 (42.4)	1704 (43.9)
Diuretics: loop^*∗*^	816 (46.2)	511 (47.8)	509 (48.6)	1836 (47.3)
Diuretics: potassium-sparing^*∗*^^†^	58 (3.3)	37 (3.5)	29 (2.8)	124 (3.2)
Total-all diuretics^*∗*^	1328 (75.2)	769 (71.9)	762 (72.7)	2859 (73.6)
Peripheral adrenergic neuron antagonist^†^	0 (0)	0 (0)	0 (0)	0 (0)
Renin inhibitors	3 (0.2)	0 (0)	5 (0.5)	8 (0.2)
No drugs	37 (2.1)	31 (2.9)	31 (3.0)	99 (2.6)
Total	1765 (100)	1069 (100)	1048 (100)	3882 (100)

^*∗*^Total-all diuretics include aldosterone antagonists; diuretics include thiazide/thiazide-type/loop/potassium-sparing. ^†^Not used alone/used in combination for chronic hypertension treatment.

**Table 2:** Patterns of antihypertensive drug utilization from Medicare Part-D drug coverage in 2007, by trial arm.

Total participants N = 8007	N (%) receiving antihypertensive drugs who took the study trial class drug as of 2007				N (%) receiving antihypertensive drugs who did not take the study trial drug as of 2007			
Diuretic = thiazide/thiazide-type (randomized to chlorthalidone in trial: 3637 (45.4)) (217 (6.0) on no drugs in 2007)	1) any diuretic (1 drug)	2) any diuretic plus 1 drug (2 drugs)	3) any diuretic plus 2 drugs (3 drugs)	4) any diuretic plus ≥3 drugs (4 + drugs)	1) no diuretic (on 1 drug)	2) no diuretic (on 2 drugs)	3) no diuretic (on 3 drugs)	4) no diuretic (on 4 + drugs)
Aldosterone antagonists ^†^		1 (0)	7 (0.2)	51 (1.4)	3 (0.1)	13 (0.4)	30 (0.8)	82 (2.3)
Alpha 1 adrenergic receptor agonist (selective; *α*-blocker)		2 (0.1)	21 (0.6)	73 (2.0)	7 (0.2)	17 (0.5)	34 (0.9)	47 (1.3)
Total ACE inhibitor + ARB		202 (5.6)	432 (11.9)	571 (15.7)	162 (4.5)	367 (10.1)	371 (10.2)	338 (9.3)
Arteriolar vasodilators		2 (0.1)	11 (0.3)	59 (1.6)	1 (0)	4 (0.1)	21 (0.6)	79 (2.2)
Autonomic ganglionic vasodilators		0 (0)	0 (0)	0 (0)	0 (0)	0 (0)	0 (0)	0 (0)
Beta adrenergic blockers (*β*-blockers)		82 (2.3)	274 (7.5)	508 (14.0)	89 (2.4)	279 (7.7)	353 (9.7)	330 (9.1)
Total CCB-dihydropyridine + nondihydropyridine		34 (0.9)	220 (6.0)	475 (13.1)	106 (2.9)	231 (6.3)	252 (6.9)	284 (7.8)
Central alpha 2 adrenergic agonists		6 (0.2)	17 (0.5)	121 (3.3)	8 (0.2)	27 (0.7)	39 (1.1)	112 (3.1)
Diuretics: thiazide/thiazide-type	82 (2.3)	366 (10.1)	539 (14.8)	626 (17.2)	0 (0)	0 (0)	0 (0)	0 (0)
Diuretics: loop		9 (0.2)	44 (1.2)	232 (6.4)	36 (1.0)	180 (4.9)	273 (7.5)	321 (8.8)
Diuretics: potassium-sparing^†^		28 (0.8)	51 (1.4)	91 (2.5)	3 (0.1)	6 (0.2)	4 (0.1)	6 (0.2)
Peripheral adrenergic neuron antagonist^†^		0 (0)	1 (0)	3 (0.1)	0 (0)	0 (0)	0 (0)	2 (0.1)
Renin inhibitors		0 (0)	0 (0)	11 (0.3)	0 (0)	0 (0)	0 (0)	0 (0)
Total participants	82 (2.3)	366 (10.1)	539 (14.8)	626 (17.2)	415 (11.4)	562 (15.4)	459 (12.6)	371 (10.2)

Calcium channel blocker (CCB) (randomized to amlodipine in trial: 2189 (27.3)) (134 (6.1) on no drugs in 2007)	1) any CCB (1 drug)	2) any CCB plus 1 drug (2 drugs)	3) any CCB plus 2 drugs (3 drugs)	4) any CCB plus ≥3 drugs (4 + drugs)	1) no CCB (on 1 drug)	2) no CCB (on 2 drugs)	3) no CCB (on 3 drugs)	4) no CCB (on 4 + drugs)
Aldosterone antagonists ^†^		2 (0.1)	5 (0.2)	32 (1.5)	1 (0)	6 (0.3)	16 (0.7)	25 (1.1)
Alpha 1 adrenergic receptor agonist (selective; *α*-blocker)		4 (0.2)	7 (0.3)	53 (2.4)	5 (0.2)	10 (0.5)	21 (1.0)	24 (1.1)
Total ACE inhibitor + ARB		114 (5.2)	237 (10.8)	428 (19.5)	99 (4.5)	259 (11.8)	220 (10.0)	100 (4.6)
Arteriolar vasodilators		0 (0)	10 (0.5)	68 (3.1)	0 (0)	6 (0.3)	7 (0.3)	21 (1)
Autonomic ganglionic vasodilators		0 (0)	0 (0)	0 (0)	0 (0)	0 (0)	0 (0)	0 (0)
Beta adrenergic blockers (*β*-blockers)		52 (2.4)	141 (6.4)	386 (17.6)	71 (3.2)	145 (6.6)	201 (9.2)	103 (4.7)
Total CCB–dihydropyridine + non-dihydropyridine	93 (4.2)	223 (10.2)	322 (14.7)	471 (21.5)	0 (0)	0 (0)	0 (0)	0 (0)
Central alpha 2 adrenergic agonists		5 (0.2)	17 (0.8)	105 (4.8)	1 (0)	8 (0.4)	24 (1.1)	25 (1.1)
Diuretics: thiazide/thiazide-type		28 (1.3)	135 (6.2)	301 (13.7)	26 (1.2)	170 (7.8)	154 (7.0)	76 (3.5)
Diuretics: loop		17 (0.8)	80 (3.7)	250 (11.4)	23 (1.1)	86 (3.9)	113 (5.2)	83 (3.8)
Diuretics: potassium-sparing^†^		1 (0)	12 (0.5)	40 (1.8)	0 (0)	9 (0.4)	16 (0.7)	12 (0.5)
Peripheral adrenergic neuron antagonist^†^		0 (0)	0 (0)	0 (0)	0 (0)	1 (0)	1 (0)	0 (0)
Renin inhibitors		0 (0)	0 (0)	2 (0.1)	0 (0)	0 (0)	1 (0)	1 (0)
Total participants	93 (4.2)	223 (10.2)	322 (14.7)	471 (21.5)	226 (10.3)	350 (16.0)	258 (11.8)	112 (5.1)

Angiotensin-converting enzyme (ACE) inhibitor/angiotensin II receptor blockers (ARB) (randomized to lisinopril in trial: 2181 (27.2)) (119 (5.5) on no drugs in 2007)	1) any ACE/ARB (1 drug)	2) any ACE/ARB plus 1 drug (2 drugs)	3) any ACE/ARB plus 2 drugs (3 drugs)	4) any ACE/ARB plus ≥3 drugs (4 + drugs)	1) no ACE/ARB (on 1 drug)	2) no ACE/ARB (on 2 drugs)	3) no ACE/ARB (on 3 drugs)	4) no ACE/ARB (on 4 + drugs)
		0 (0)	7 (0.3)	63 (2.9)	3 (0.1)	10 (0.5)	12 (0.5)	6 (0.3)
Alpha 1 adrenergic receptor agonist (selective; *α*-blocker)		6 (0.3)	24 (1.1)	68 (3.1)	5 (0.2)	12 (0.5)	7 (0.3)	6 (0.3)
Total ACE inhibitor + ARB	126 (5.8)	382 (17.5)	526 (24.1)	528 (24.2)	0 (0)	0 (0)	0 (0)	0 (0)
Arteriolar vasodilators		1 (0)	19 (0.9)	88 (4)	1 (0)	13 (0.6)	12 (0.5)	19 (0.9)
Autonomic ganglionic vasodilators		0 (0)	0 (0)	0 (0)	0 (0)	0 (0)	0 (0)	0 (0)
Beta adrenergic blockers (*β*-blockers)		92 (4.2)	292 (13.4)	434 (19.9)	72 (3.3)	105 (4.8)	91 (4.2)	35 (1.6)
Total CCB-dihydropyridine + nondihydropyridine		98 (4.5)	231 (10.6)	408 (18.7)	52 (2.4)	70 (3.2)	79 (3.6)	30 (1.4)
Central alpha 2 adrenergic agonists		6 (0.3)	29 (1.3)	135 (6.2)	4 (0.2)	15 (0.7)	13 (0.6)	17 (0.8)
Diuretics: thiazide/thiazide-type		133 (6.1)	262 (12.0)	334 (15.3)	18 (0.8)	47 (2.2)	58 (2.7)	25 (1.1)
Diuretics: loop		46 (2.1)	172 (7.9)	303 (13.9)	22 (1.0)	52 (2.4)	61 (2.8)	26 (1.2)
Diuretics: potassium-sparing^†^		0 (0)	16 (0.7)	46 (2.1)	1 (0)	2 (0.1)	17 (0.8)	10 (0.5)
Peripheral adrenergic neuron antagonist^†^		0 (0)	0 (0)	3 (0.1)	0 (0)	0 (0)	0 (0)	0 (0)
Renin inhibitors		0 (0)	0 (0)	2 (0.1)	1 (0)	0 (0)	1 (0)	0 (0)
Total participants	126 (5.8)	382 (17.5)	526 (24.1)	528 (24.2)	179 (8.2)	163 (7.5)	117 (5.4)	41 (1.9)

^**†**^Aldosterone antagonists.

**Table 3:** Adjusted hazard ratio (95% CI) of morbidity and mortality by the end of posttrial (12-31-2017) period by 3 arms.

Total participants N = 8007	In trial participants who received the study trial drug				In trial participants who did not receive the study trial drug				
Diuretic = thiazide/thiazide-type (randomized to chlorthalidone in trial: 3637 (45.4))	1) any diuretic (1 drug)	2) any diuretic plus 1 drug (2 drugs)	3) any diuretic plus 2 drugs (3 drugs)	4) any diuretic plus ≥3 drugs (4 + drugs)	1) no diuretic (on 1 drug)	2) no diuretic (on 2 drugs)	3) no diuretic (on 3 drugs)	4) no diuretic (on 4 + drugs)	5) No drugs
*Mortality outcomes*									
All-cause mortality	1.00 (ref)	1.01 (0.71-1.43)	0.93 (0.65-1.31)	1.16 (0.82-1.62)	1.11 (0.79-1.56)	1.17 (0.84-1.64)	1.45 (1.03-2.05)	1.97 (1.38-2.81)	1.12 (0.77-1.62)
CVD mortality	1.00 (ref)	1.02 (0.60-1.75)	0.84 (0.49-1.44)	1.16 (0.70-1.95)	1.01 (0.59-1.72)	1.12 (0.67-1.87)	1.66 (0.99-2.77)	2.40 (1.42-4.08)	0.95 (0.53-1.70)
CHD mortality	1.00 (ref)	0.90 (0.40-2.01)	0.63 (0.28-1.43)	1.22 (0.57-2.60)	0.83 (0.38-1.84)	0.99 (0.46-2.16)	1.88 (0.87-4.04)	2.82 (1.29-6.15)	0.94 (0.40-2.25)
Stroke mortality	1.00 (ref)	0.81 (0.21-3.16)	0.69 (0.17-2.75)	1.20 (0.33-4.38)	1.44 (0.38-5.43)	1.06 (0.30-3.69)	1.63 (0.41-6.44)	1.41 (0.28-6.96)	0.75 (0.15-3.75)
Heart failure mortality	1.00 (ref)	1.04 (0.12-8.97)	1.03 (0.20-5.36)	0.69 (0.17-2.80)	0.74 (0.14-3.91)	0.76 (0.17-3.35)	0.77 (0.17-3.44)	1.97 (0.37-10.42)	0.60 (0.06-5.71)
Other CVD mortality	1.00 (ref)	1.67 (0.56-4.96)	1.46 (0.48-4.47)	1.33 (0.45-3.96)	1.37 (0.45-4.20)	1.68 (0.59-4.77)	2.41 (0.84-6.98)	3.38 (1.14-10.05)	1.65 (0.48-5.64)
Non-CVD mortality	1.00 (ref)	1.04 (0.65-1.66)	1.00 (0.63-1.58)	1.17 (0.74-1.84)	1.21 (0.77-1.90)	1.19 (0.76-1.87)	1.29 (0.81-2.05)	1.68 (1.04-2.72)	1.27 (0.78-2.07)
Cancer	1.00 (ref)	1.79 (0.62-5.16)	1.35 (0.47-3.90)	1.58 (0.54-4.58)	1.51 (0.52-4.40)	1.77 (0.62-5.06)	1.98 (0.68-5.81)	2.45 (0.76-7.84)	1.76 (0.56-5.51)
Kidney disease	1.00 (ref)	− (^*∗*^-^*∗*^)	− (^*∗*^-^*∗*^)	− (^*∗*^-^*∗*^)	− (^*∗*^-^*∗*^)	− (^*∗*^^*∗*^)	− (^*∗*^-^*∗*^)	− (^*∗*^-^*∗*^)	− (^*∗*^-^*∗*^)
Accident/suicide/homicide	1.00 (ref)	− (^*∗*^-^*∗*^)	0.87 (0.09-8.63)	1.00 (0.10-10.51)	1.38 (0.16-11.84)	0.99 (0.09-10.36)	0.99 (0.08-12.78)	1.91 (0.19-19.59)	0.33 (0.01-11.19)
Other non-CVD disease	1.00 (ref)	0.81 (0.47-1.40)	0.87 (0.52-1.47)	1.00 (0.59-1.70)	1.10 (0.66-1.84)	1.00 (0.60-1.67)	1.05 (0.61-1.80)	1.45 (0.83-2.52)	1.13 (0.65-1.99)

*Combined fatal and nonfatal hospitalized events*									
CVD	1.00 (ref)	0.79 (0.50-1.25)	1.02 (0.66-1.58)	1.15 (0.75-1.77)	0.98 (0.63-1.53)	1.05 (0.68-1.63)	1.60 (1.04-2.49)	2.44 (1.57-3.81)	0.82 (0.50-1.36)
CHD	1.00 (ref)	0.88 (0.43-1.80)	1.01 (0.51-2.03)	1.43 (0.73-2.82)	1.02 (0.51-2.03)	1.06 (0.53-2.10)	1.93 (0.98-3.80)	2.96 (1.48-5.89)	0.94 (0.44-2.03)
Heart failure	1.00 (ref)	0.72 (0.39-1.32)	1.24 (0.71-2.19)	1.32 (0.76-2.28)	1.05 (0.59-1.87)	1.23 (0.71-2.15)	1.94 (1.11-3.38)	3.09 (1.77-5.40)	0.91 (0.48-1.75)
Stroke	1.00 (ref)	1.41 (0.58-3.43)	1.07 (0.45-2.57)	1.38 (0.57-3.32)	1.30 (0.52-3.20)	1.51 (0.64-3.56)	1.42 (0.58-3.48)	1.93 (0.77-4.84)	1.15 (0.43-3.11)
Cancer	1.00 (ref)	1.01 (0.51-1.98)	0.81 (0.42-1.58)	1.01 (0.52-1.96)	0.73 (0.37-1.43)	0.96 (0.50-1.86)	1.24 (0.63-2.44)	1.19 (0.58-2.45)	0.97 (0.46-2.03)
Kidney disease/ESRD	1.00 (ref)	0.81 (0.08-7.89)	1.36 (0.16-11.59)	3.07 (0.40-23.31)	1.58 (0.19-13.16)	3.07 (0.40-23.29)	2.67 (0.33-21.50)	4.91 (0.63-38.06)	3.25 (0.17-63.50)

Calcium channel blocker (CCB) (randomized to amlodipine in trial: 2189 (27.3))	1) any CCB (1 drug)	2) any CCB plus 1 drug (2 drugs)	3) any CCB plus 2 drugs (3 drugs)	4) any CCB plus ≥3 drugs (4 + drugs)	1) no CCB (on 1 drug)	2) no CCB (on 2 drugs)	3) no CCB (on 3 drugs)	4) no CCB (on 4 + drugs)	5) No Drugs
*Mortality outcomes*									
All-cause mortality	1.00 (ref)	0.92 (0.67-1.27)	1.04 (0.76-1.42)	1.23 (0.91-1.66)	0.97 (0.70-1.35)	0.97 (0.71-1.32)	1.15 (0.83-1.60)	1.76 (1.16-2.67)	1.06 (0.71-1.56)
CVD mortality	1.00 (ref)	0.78 (0.45-1.34)	1.36 (0.82-2.24)	1.36 (0.83-2.23)	1.05 (0.61-1.78)	0.97 (0.58-1.61)	1.48 (0.89-2.47)	2.41 (1.23-4.73)	0.99 (0.53-1.88)
CHD mortality	1.00 (ref)	2.49 (0.65-9.49)	4.54 (1.34-15.40)	4.97 (1.50-16.48)	3.71 (1.05-13.04)	2.90 (0.85-9.86)	5.03 (1.39-18.25)	8.87 (2.11-37.23)	4.42 (1.09-17.95)
Stroke mortality	1.00 (ref)	1.81 (0.14-22.57)	3.77 (0.47-30.42)	3.35 (0.41-27.26)	2.18 (0.20-24.06)	3.39 (0.42-27.53)	4.34 (0.50-37.46)	2.80 (0.08-96.99)	7.12 (0.59-85.51)
Heart failure mortality	1.00 (ref)	1.64 (0.08-34.42)	6.41 (0.73-56.17)	5.54 (0.69-44.29)	3.00 (0.28-32.28)	1.17 (0.10-14.23)	7.47 (0.71-78.01)	70.21 (4.06-1213.94)	− (^*∗*^-^*∗*^)
Other CVD mortality	1.00 (ref)	0.48 (0.23-0.97)	0.43 (0.21-0.87)	0.36 (0.18-0.72)	0.35 (0.16-0.75)	0.39 (0.20-0.77)	0.62 (0.31-1.24)	0.37 (0.12-1.20)	0.30 (0.10-0.86)
Non-CVD mortality	1.00 (ref)	1.00 (0.67-1.49)	0.84 (0.56-1.26)	1.15 (0.78-1.68)	0.95 (0.63-1.44)	0.95 (0.65-1.41)	0.95 (0.62-1.45)	1.42 (0.82-2.44)	1.06 (0.64-1.74)
Cancer	1.00 (ref)	1.09 (0.48-2.47)	0.66 (0.29-1.53)	0.94 (0.43-2.09)	1.03 (0.43-2.47)	0.73 (0.33-1.60)	0.77 (0.31-1.90)	1.23 (0.39-3.88)	0.76 (0.23-2.46)
Kidney disease	1.00 (ref)	0.49 (0.10-2.47)	0.17 (0.03-1.16)	0.74 (0.20-2.78)	0.93 (0.20-4.38)	0.29 (0.05-1.71)	0.72 (0.11-4.50)	1.01 (0.18-5.74)	− (^*∗*^-^*∗*^)
Accident/suicide/homicide	1.00 (ref)	− (^*∗*^-^*∗*^)	0.39 (0.03-5.14)	0.83 (0.08-8.40)	0.73 (0.06-9.72)	0.54 (0.07-4.41)	− (^*∗*^-^*∗*^)	− (^*∗*^-^*∗*^)	− (^*∗*^-^*∗*^)
Other non-CVD disease	1.00 (ref)	1.09 (0.65-1.82)	1.02 (0.61-1.70)	1.35 (0.83-2.19)	0.93 (0.55-1.58)	1.17 (0.71-1.91)	1.20 (0.70-2.05)	1.84 (0.92-3.68)	1.55 (0.84-2.85)

*Combined fatal and nonfatal hospitalized events*									
CVD	1.00 (ref)	1.23 (0.74-2.04)	1.76 (1.10-2.83)	2.21 (1.39-3.52)	1.42 (0.86-2.35)	1.37 (0.84-2.22)	1.93 (1.17-3.17)	2.77 (1.53-5.04)	1.58 (0.85-2.93)
CHD	1.00 (ref)	1.90 (0.78-4.61)	2.91 (1.30-6.51)	2.94 (1.32-6.53)	1.89 (0.80-4.46)	1.79 (0.78-4.08)	2.46 (1.03-5.85)	5.60 (1.92-16.31)	2.80 (1.09-7.22)
Heart failure	1.00 (ref)	0.86 (0.50-1.49)	1.36 (0.83-2.23)	1.78 (1.10-2.87)	0.86 (0.50-1.50)	1.06 (0.63-1.76)	1.76 (1.05-2.95)	3.06 (1.66-5.67)	1.02 (0.51-2.04)
Stroke	1.00 (ref)	2.09 (0.69-6.28)	2.51 (0.88-7.18)	2.58 (0.90-7.37)	2.37 (0.79-7.13)	3.23 (1.14-9.17)	3.21 (1.04-9.91)	3.82 (0.90-16.14)	2.74 (0.63-11.91)
Cancer	1.00 (ref)	1.14 (0.57-2.28)	0.80 (0.41-1.56)	1.08 (0.57-2.05)	0.94 (0.47-1.89)	0.87 (0.46-1.68)	0.79 (0.38-1.64)	1.70 (0.66-4.37)	0.81 (0.33-1.96)
Kidney disease/ESRD	1.00 (ref)	0.24 (0.07-0.89)	0.56 (0.20-1.53)	0.89 (0.36-2.21)	0.59 (0.19-1.78)	0.44 (0.16-1.25)	0.32 (0.08-1.25)	0.84 (0.25-2.80)	0.00 (0.00-1.17)

Angiotensin-converting enzyme (ACE) inhibitor/angiotensin II receptor blockers (ARB) (randomized to lisinopril in trial: 2181 (27.2))	1) any ACE/ARB (1 drug)	2) any ACE/ARB plus 1 drug (2 drugs)	3) any ACE/ARB plus 2 drugs (3 drugs)	4) any ACE/ARB plus ≥3 drugs (4 + drugs)	1) no ACE/ARB (on 1 drug)	2) no ACE/ARB (on 2 drugs)	3) no ACE/ARB (on 3 drugs)	4) no ACE/ARB (on 4 + drugs)	5) No Drugs
*Mortality outcomes*									
All-cause mortality	1.00 (ref)	0.85 (0.63–1.13)	1.11 (0.85–1.46)	1.21 (0.91–1.62)	1.06 (0.75–1.50)	1.05 (0.75–1.46)	1.01 (0.68–1.50)	0.85 (0.48–1.48)	0.97 (0.67–1.40)
CVD mortality	1.00 (ref)	0.76 (0.47–1.23)	1.22 (0.78–1.91)	1.67 (1.05–2.64)	1.14 (0.66–1.98)	1.24 (0.73–2.10)	0.84 (0.43–1.64)	0.59 (0.21–1.62)	0.92 (0.49–1.72)
CHD mortality	1.00 (ref)	0.57 (0.28–1.17)	1.11 (0.58–2.11)	1.44 (0.74–2.79)	1.33 (0.61–2.88)	0.83 (0.37–1.85)	1.45 (0.62–3.38)	1.25 (0.29–5.44)	0.59 (0.22–1.59)
Stroke mortality	1.00 (ref)	0.93 (0.30–2.90)	0.55 (0.17–1.75)	1.21 (0.41–3.53)	1.14 (0.20–6.33)	1.87 (0.57–6.08)	− (^*∗*^-^*∗*^)	− (^*∗*^-^*∗*^)	0.34 (0.06–1.78)
Heart failure mortality	1.00 (ref)	1.05 (0.19–5.85)	1.64 (0.35–7.74)	3.27 (0.67–15.95)	3.59 (0.02–522.24)	7.74 (0.97–61.48)	− (^*∗*^-^*∗*^)	1.21 (0.01–130.04)	− (^*∗*^-^*∗*^)
Other CVD mortality	1.00 (ref)	0.87 (0.33–2.29)	1.83 (0.75–4.47)	2.13 (0.84–5.43)	1.16 (0.40–3.39)	0.71 (0.21–2.38)	0.42 (0.09–2.00)	0.43 (0.05–3.53)	2.38 (0.68–8.29)
Non-CVD mortality	1.00 (ref)	0.90 (0.63–1.30)	1.05 (0.74–1.49)	0.93 (0.64–1.37)	1.01 (0.65–1.58)	0.94 (0.60–1.45)	1.11 (0.68–1.80)	1.02 (0.52–1.99)	0.99 (0.63–1.56)
Cancer	1.00 (ref)	0.62 (0.30–1.27)	0.89 (0.46–1.72)	0.49 (0.23–1.05)	0.91 (0.40–2.08)	0.55 (0.23–1.28)	0.73 (0.26–2.04)	0.63 (0.15–2.55)	1.34 (0.60–2.99)
Kidney disease	1.00 (ref)	1.92 (0.21–17.40)	1.00 (0.10–9.65)	5.78 (0.68–49.00)	7.22 (0.64–81.59)	3.52 (0.32–39.20)	0.80 (0.01–68.65)	− (^*∗*^-^*∗*^)	6.01 (0.40-–0.29)
Accident/suicide/homicide	1.00 (ref)	1.37 (0.26–7.25)	1.12 (0.19–6.43)	1.02 (0.12–8.86)	1.39 (0.11–17.16)	3.26 (0.29–36.35)	− (^*∗*^-^*∗*^)	− (^*∗*^-^*∗*^)	− (^*∗*^-^*∗*^)
Other non-CVD disease	1.00 (ref)	0.97 (0.62–1.52)	1.18 (0.76–1.82)	0.98 (0.61–1.57)	0.88 (0.50–1.57)	0.97 (0.55–1.70)	1.34 (0.74–2.42)	0.91 (0.37–2.19)	0.70 (0.38–1.29)
*Combined fatal and nonfatal hospitalized events*									
CVD	1.00 (ref)	0.84 (0.56–1.27)	1.43 (0.98–2.09)	1.78 (1.19–2.64)	0.91 (0.56–1.49)	1.22 (0.77–1.94)	1.05 (0.61–1.81)	0.81 (0.36–1.82)	0.69 (0.38–1.24)
CHD	1.00 (ref)	0.59 (0.33–1.06)	1.14 (0.67–1.93)	1.27 (0.73–2.22)	0.82 (0.41–1.62)	0.91 (0.47–1.74)	1.00 (0.48–2.10)	0.60 (0.17–2.06)	0.44 (0.18–1.07)
Heart failure	1.00 (ref)	1.17 (0.70–1.95)	1.79 (1.10–2.89)	2.68 (1.64–4.41)	0.99 (0.52–1.90)	1.86 (1.04–3.31)	1.36 (0.69–2.66)	1.01 (0.38–2.66)	0.78 (0.36–1.69)
Stroke	1.00 (ref)	0.80 (0.40–1.60)	1.06 (0.56–2.00)	1.05 (0.53–2.06)	0.73 (0.32–1.69)	1.01 (0.47–2.20)	0.30 (0.09–1.04)	0.36 (0.09–1.53)	0.89 (0.35–2.25)
Cancer	1.00 (ref)	0.64 (0.35–1.18)	0.90 (0.52–1.56)	0.68 (0.37–1.23)	0.86 (0.43–1.75)	0.74 (0.37–1.48)	0.81 (0.35–1.88)	0.63 (0.19–2.06)	1.12 (0.55–2.31)
Kidney disease/ESRD	1.00 (ref)	3.62 (0.44–29.84)	3.51 (0.44–27.92)	8.80 (1.17–66.24)	6.99 (0.78–62.32)	6.15 (0.57–66.57)	9.88 (0.44–220.67)	629.84 (3.28–120934.30)	13.46 (1.37–132.69)

^*∗*^Likelihood did not converge due to the small number of events. Baseline covariates in the adjusted hazard ratio models include age (years), race (black/non-black), gender, education (years), current smoking status, BMI, entry criterion HDL<35, LDL, GFR, history of diabetes, antihypertensive treatment, on pravastatin, and SBP. In-trial covariate included final SBP.

**Table 4:** Adjusted hazard ratio (95% CI) of morbidity and mortality by the end of posttrial (12-31-2017) period.

Total participants N = 8007	Randomization class, non-crossover^*∗*^					
	1) Diuretic (N = 463 (5.8%))	2) CCB (N = 685 (8.6%))	3) ACE/ARB (N = 1,444 (18.0%))	4) 2 + of diuretic/ CCB/ACE/ARB OR non-RZ class AHTs (N = 4,945 (61.8%))	5) No drugs (N = 470 (5.9%))	ACE/ARB vs CCB (N = 2,129 (26.6%0)
*Mortality outcomes*						
All-cause mortality	1.00 (ref)	1.17 (0.99–1.37)	1.26 (1.09–1.45)	1.08 (0.95–1.23)	1.04 (0.87–1.24)	1.04 (0.92–1.17)
CVD mortality	1.00 (ref)	1.12 (0.87–1.44)	1.29 (1.03–1.61)	1.07 (0.88–1.31)	0.90 (0.68–1.20)	1.10 (0.91–1.33)
CHD mortality	1.00 (ref)	1.25 (0.84–1.85)	1.39 (0.98–1.99)	1.30 (0.94–1.80)	0.98 (0.63–1.54)	0.99 (0.75–1.32)
Stroke mortality	1.00 (ref)	0.91 (0.46–1.77)	1.17 (0.66–2.08)	0.94 (0.57–1.56)	1.00 (0.48–2.09)	1.28 (0.75–2.20)
Heart failure mortality	1.00 (ref)	0.59 (0.27–1.31)	0.88 (0.47–1.65)	0.91 (0.53–1.57)	0.57 (0.24–1.34)	1.31 (0.66–2.57)
Other CVD mortality	1.00 (ref)	1.26 (0.82–1.94)	1.38 (0.94–2.03)	0.94 (0.66–1.33)	0.89 (0.53–1.48)	1.15 (0.83–1.60)
Non-CVD Mortality	1.00 (ref)	1.19 (0.96–1.47)	1.23 (1.01–1.48)	1.08 (0.91–1.28)	1.12 (0.89–1.41)	1.00 (0.85–1.17)
Cancer	1.00 (ref)	1.10 (0.71–1.70)	1.21 (0.83–1.78)	1.01 (0.72–1.43)	1.33 (0.84–2.11)	1.16 (0.82–1.65)
Kidney disease	1.00 (ref)	2.61 (1.04–6.54)	1.44 (0.58–3.59)	1.39 (0.60–3.19)	0.86 (0.25–3.02)	0.55 (0.31–0.95)
Accident/suicide/homicide	1.00 (ref)	0.85 (0.27–2.63)	1.12 (0.43–2.89)	0.97 (0.42–2.28)	0.46 (0.11–1.85)	1.22 (0.50–2.96)
Other non-CVD disease	1.00 (ref)	1.15 (0.88–1.49)	1.22 (0.97–1.54)	1.08 (0.88–1.33)	1.11 (0.83–1.47)	1.01 (0.83–1.22)
*Combined fatal and non-fatal hospitalized events*						
CVD	1.00 (ref)	1.30 (1.04–1.61)	1.49 (1.22–1.81)	1.28 (1.07–1.54)	0.93 (0.71–1.21)	1.06 (0.90–1.24)
CHD	1.00 (ref)	1.31 (0.94–1.81)	1.35 (1.00–1.81)	1.25 (0.96–1.63)	0.93 (0.63–1.36)	0.97 (0.77–1.23)
Heart failure	1.00 (ref)	1.49 (1.14–1.95)	1.69 (1.33–2.16)	1.45 (1.16–1.81)	0.93 (0.67–1.29)	1.08 (0.90–1.30)
Stroke	1.00 (ref)	1.04 (0.69–1.58)	1.60 (1.11–2.29)	1.24 (0.89–1.72)	1.10 (0.68–1.77)	1.38 (1.02–1.87)
Cancer	1.00 (ref)	1.02 (0.73–1.44)	1.07 (0.79–1.44)	0.93 (0.71–1.20)	1.06 (0.73–1.53)	1.07 (0.82–1.41)
Kidney disease/ESRD	1.00 (ref)	3.75 (1.82–7.73)	1.96 (0.95–4.05)	2.07 (1.06–4.05)	2.15 (0.92–5.07)	0.50 (0.34–0.74)

^*∗*^Randomization class indicates on at least a diuretic or CCB or ACE/ARB without class crossover. Abbreviations: ACE/ARB = ACE-inhibitor or alpha receptor blocker; AHT = antihypertensive; CHD = coronary heart disease; CCB = calcium channel blocker; CVD = cardiovascular disease; ESRD = end-stage renal disease. Baseline covariates in the adjusted hazard ratio models include age (years), race (black/non-black), gender, education (years), current smoking status, BMI, entry criterion HDL<35, LDL, GFR, history of diabetes, antihypertensive treatment, on pravastatin, and SBP. In-trial covariate included final SBP.

## Data Availability

The ALLHAT data, Medicare Claims data, Medicare Part-D, and National Death Index (NDI) data are not public-use datasets. However, researchers may request the ALLHAT data with the approval from the ALLHAT Coordinating Center in Houston, the Medicare Claims data and Medicare Part-D data with the approval from the Center for Medicare and Medicaid Services (CMS), and the National Death Index (NDI) data with the approval from the National Center for Health Statistics (NCHS). The authors plan to share the statistical models and statistical programs that were used to analyze these data upon request.

## Supplementary Materials

Supplementary Materials Table S1: systemic antihypertensive medication classes and common generic and brand names. Table S2 (a–e): ICD-9 and ICD-10 codes for heart failures (a), for ischemic heart disease, or coronary heart disease (CHD) or coronary artery disease (b), cerebrovascular disease or stroke (c), cancer (d), and kidney disease or other complications due to renal dialysis (e). Table S3: baseline characteristics of participants eligible for Medicare and enrolled for Medicare Part-D by three comparison groups in 2007. Table S4 (a–d): patterns of antihypertensive drug utilization from Medicare Part-D drug coverage in 2007, by 3 arms and added-on drugs by black (a), non-black (b), male (c), and female (d). Table S5: cumulative incidence for morbidity/mortality outcomes between 2007 and 2017 by the changing patterns of antihypertensive drugs as of 2007. Table S6: morbidity/mortality outcomes between 2007 and 2017 by the pattern of antihypertensive drug use. Table S7 (a, b): adjusted hazard ratio (95% CI) of morbidity and mortality by the end of posttrial (12-31-2017) period by 3 arms (a) and by posttrial drug uses (b).
