# Supplementary material for: Effects of Posttrial Antihypertensive Drugs on Morbidity and Mortality: Findings from 15-Year Passive Follow-Up after ALLHAT Ended
Source: Int J Hypertens. 2021 Dec 9;2021:2261144. doi: 10.1155/2021/2261144 (PMC8677412; doi:10.1155/2021/2261144)
Supplement: Supplementary Materials — Table S1: systemic antihypertensive medication classes and common generic and brand names. Table S2 (a–e): ICD-9 and ICD-10 codes for heart failures (a), for ischemic heart disease, or coronary heart disease (CHD) or coronary artery disease (b), cerebrovascular disease or stroke (c), cancer (d), and kidney disease or other complications due to renal dialysis (e). Table S3: baseline characteristics of participants eligible for Medicare and enrolled for Medicare Part-D by three comparison groups in 2007. Table S4 (a–d): patterns of antihypertensive drug utilization from Medicare Part-D drug coverage in 2007, by 3 arms and added-on drugs by black (a), non-black (b), male (c), and female (d). Table S5: cumulative incidence for morbidity/mortality outcomes between 2007 and 2017 by the changing patterns of antihypertensive drugs as of 2007. Table S6: morbidity/mortality outcomes between 2007 and 2017 by the pattern of antihypertensive drug use. Table S7 (a, b): adjusted hazard ratio (95% CI) of morbidity and mortality by the end of posttrial (12-31-2017) period by 3 arms (a) and by posttrial drug uses (b). [file 2261144.f1.pdf]

Supplemental Tables:

Table S1. Systemic Antihypertensive Medication Classes and Common Generic and Brand Names

#	Antihypertensive medication classes<br>FDA Chronic Antihypertensive Treatment Classes	Generic Name (GNN)	Brand Name (BN)
1	Aldosterone antagonists †	Eplerenone<br>Spironolactone	Inspra<br>Aldactone, Carospir, Aldactazide‡
2	Alpha 1 adrenergic receptor agonist (selective; **α-blocker**)	**Doxazosin [Step 1 in ALLHAT]**<br>Prazosin<br>Terazosin	Cardura (XL)<br>Minipress, Prazin, Prazo<br>Hytrin
3	Angiotensin-converting enzyme (**ACE**) inhibitor	Benazepril<br>Captopril<br>Enalapril<br>Fosinopril<br>**Lisinopril [Step I]**<br>Moexipril<br>Perindopril<br>Quinapril<br>Ramipril<br>Trandolapril	Lotensin, Lotensin HCT‡, Lotrel‡<br>Capoten, Captoril, Capozide‡<br>Vasotec, Enalaprilat, Epaned, Lexxel‡§, Vaseretic‡<br>Monopril<br>Prinivil, Qbrelis, Zestril, Zestoretic‡<br>Univasc, Uniretic‡<br>Aceon, Prestalia‡<br>Accupril, Accuretic‡<br>Altace<br>Mavik, Tarka‡
4	Angiotensin II receptor blockers (**ARB**)	Azilsartan<br>Candesartan<br>Eprosartan<br>Irbesartan<br>Losartan<br>Olmesartan<br>Telmisartan<br>Valsartan	Edarbi, Edarbychlor‡<br>Atacand, Atacand HCT‡<br>Teveten, Teveten HCT‡, Teveten Plus‡<br>Avapro, Avalide‡<br>Cozaar, Hyzaar‡<br>Benicar, Azor‡, Benicar HCT‡, Tribenzor‡<br>Micardis, Micardis HCT‡, Micardis Plus‡,Twynsta‡<br>Diovan, Prexxartan§, Byvalson‡, Diovan HCT‡, Exforge HCT‡, Entresto‡, Valturna‡
5	Arteriolar vasodilators	**Hydralazine [Step 3 in ALLHAT]**<br>Minoxidil	-, Hydra-zide‡, *BiDil*<br>Loniten, Minodyl, Minoxidil HTN
6	Autonomic ganglionic vasodilators	Mecamylamine	-
7	Beta adrenergic blockers (**β-blockers**)	Acebutolol<br>**Atenolol [Step 2 in ALLHAT]**<br>Betaxolol<br>Bisoprolol<br>Carvedilol {*also with α1 properties*}<br>Labetalol {*also with α1 properties*}<br>Metoprolol<br><br>Nadolol<br>Nebivolol<br>*Pindolol*<br>Propranolol	Sectral<br>Tenormin, Tenoretic‡<br>Kerlone<br>Monocor, Zebeta, Ziac‡<br>Coreg, Coreg CR<br>Trandate<br>Kapspargo Sprinkle, Lopressor, Toprol XL, Dutoprol‡, Lopressor HCT‡<br>Corgard, Corzide‡<br>Bystolic, Byvalson‡<br>*Visken*<br>Inderal, Inderal LA, InnoPran XL, Inderide‡

8	Calcium channel blocker (CCB)-<br>dihydropyridine	**Amlodipine [Step I]**<br><br>Felodipine<br><br><br>Isradipine<br>Nicardipine<br>Nifedipine<br><br>Nisoldipine	Norvasc, Amturnide‡, Azor‡, Exforge HCT‡, Lotrel‡, Prestalia‡,<br>Tekamlo‡, Tribenzor‡, Twynsta‡<br>Cabren, Cardioplen XL, Felendil XL, Felogen XL, Felotens XL,<br>Keloc SR, Neofel XL, Plendil, Renedil, Vascalpha, Lexxel‡§<br>-<br>Cardene, Cardene SR<br>Adalat, Adalat CC, Afeditab CR, Nifediac CC, Nifedical XL,<br>Procardia, Procardia XL<br>Sular
9	Calcium channel blocker – non-<br>dihydropyridine	Diltiazem<br><br><br>Verapamil	Cardizem, Cardizem CD, Cardizem LA, *Cartia XT,* Dilacor, Dilacor<br>XR, Dilatrate, Diltazem, Diltazem CD, *Diltia XT,* Diltiaz, Diltiaz CD,<br>Diltiaz SR, *Diltzac, Taztia,* Tiazac<br>Calan, Calan HS, Calan SR, Covera HS, Isoptin, Isoptin SR,<br>Verelan, Verelan PM, Tarka‡
10	Central alpha 2 adrenergic agonists	**Clonidine [Step 2]**<br>Guanfacine<br>Methyldopa	Catapres, Jenloga, Kapvay, Nexiclon XR, Clorpres‡<br>Intuniv, Tenex
11	Diuretics: thiazide	Bendroflumenthiazide<br>Chlorothiazide<br>Hydrochlorothiazide	Naturetin§, NeoNaClex§, Urizide§, Corzide‡<br>Diuril, Tekturna HCT‡<br>Microzide, Accuretic‡, Aldactazide‡, Atacand HCT‡, Avalide‡,<br>Benicar HCT‡, Capozide‡, Corzide‡, Dutoprol‡, Exforge HCT‡,<br>Hydra-zide‡, Hyzaar‡, Inderide‡, Lopressor HCT‡, Lotensin HCT‡,<br>Maxide‡, Micardis HCT‡, Micardis Plus‡, Moduretic‡, Teveten HCT‡,<br>Teveten Plus‡, Tribenzor‡, Uniretic‡, Vaseretic‡, Zestoretic‡, Ziac‡
	Diuretics: thiazide-type	**Chlorthalidone [Step I]**<br><br>Indapamide<br>Metolazone	Hygroton, Thalitone, Chlorthalid, Clorpres‡, Edarbychlor‡, Tenoretic‡<br>-<br>Zaroxolyn
	Diuretics: loop	Bumetanide<br>Furosemide<br>Torsemide	Bumex, Burinex<br>Lasix<br>Demadex
	Diuretics: potassium-sparing†	Amiloride<br>Triamterene	Midamor, Moduretic‡<br>Dyrenium, *Dyazide,* Maxide‡
12	Peripheral adrenergic neuron<br>antagonist†	**Reserpine [Step 2]**	Serpasil
13	Renin inhibitors	Aliskiren	Tekturna, Tekturna HCT‡, Teklamo‡, Amturnide‡, Valturna‡

†Not used alone/used in combination for chronic hypertension treatment
‡Combination medication also listed in another class
§Currently discontinued in the US but in use during ALLHAT

Table S2a. ICD-9 and ICD-10 Codes for Heart Failures

ICD-10 Codes	Description	ICD-9 Codes	Description
I09.81	Rheumatic heart failure	398.91	Rheumatic heart failure (congestive)
I09.89	Other specified rheumatic heart diseases	398.99	Other rheumatic heart diseases
I11.0	Hypertensive heart disease with heart failure	402.01	Malignant hypertensive heart disease with heart failure
I11.0	Hypertensive heart disease with heart failure	402.11	Benign hypertensive heart disease with heart failure
I11.0	Hypertensive heart disease with heart failure	402.91	Unspecified hypertensive heart disease with heart failure
I13.0	Hypertensive heart and chronic kidney disease with heart failure and stage 1 through stage 4 chronic kidney disease, or unspecified chronic kidney disease	404.01	Malignant hypertensive heart and renal disease with heart failure
I13.2	Hypertensive heart and chronic kidney disease with heart failure and with stage 5 chronic kidney disease, or end stage renal disease	404.03	Malignant hypertensive heart and renal disease with heart failure and renal failure
I13.0	Hypertensive heart and chronic kidney disease with heart failure and stage 1 through stage 4 chronic kidney disease, or unspecified chronic kidney disease	404.11	Benign hypertensive heart and renal disease with heart failure
I13.2	Hypertensive heart and chronic kidney disease with heart failure and with stage 5 chronic kidney disease, or end stage renal disease	404.13	Benign hypertensive heart and renal disease with heart failure and renal failure
I13.0	Hypertensive heart and chronic kidney disease with heart failure and stage 1 through stage 4 chronic kidney disease, or unspecified chronic kidney disease	404.91	Unspecified hypertensive heart and renal disease with heart failure
I13.2	Hypertensive heart and chronic kidney disease with heart failure and with stage 5 chronic kidney disease, or end stage renal disease	404.93	Unspecified hypertensive heart and renal disease with heart failure and renal failure
I26.09	Other pulmonary embolism with acute cor pulmonale	415	Acute pulmonary heart disease
I40.9	Acute myocarditis, unspecified	422.90	Acute myocarditis, unspecified
I42.5	Other restrictive cardiomyopathy	425.4	Other primary cardiomyopathies
I42.7	Cardiomyopathy due to drug and external agent	425.9	Secondary cardiomyopathy, unspecified
I50	Heart failure	428	Heart failure

Table S2b. ICD-9 and ICD-10 Codes for Ischemic heart disease, or coronary heart disease (CHD) or coronary artery disease

ICD-10 Codes	Description	ICD9 Codes	Description
I21.x	Acute myocardial infarction	410.x	Acute myocardial infarction
I24.x	Other acute and subacute forms of ischemic heart disease	411.x	Other acute and subacute forms of ischemic heart disease
I25.2	Old myocardial infarction	412.x	Old myocardial infarction
I20.x	Angina pectoris	413.x	Angina pectoris
I25.x	Other forms of chronic ischemic heart disease	414.x	Other forms of chronic ischemic heart disease

Table S2c. ICD-9 and ICD-10 Codes for Cerebrovascular Disease or Stroke

ICD-10 Codes	Description	ICD-9 Codes	Description
I60.x	Nontraumatic subarachnoid hemorrhage, unspecified	430.x	Subarachnoid hemorrhage
I61.x	Nontraumatic intracerebral hemorrhage, unspecified	431.x	Intracerebral hemorrhage
I62.x	Nontraumatic extradural hemorrhage	432.x	Other and unspecified intracranial hemorrhage
I65.x	Occlusion and stenosis of ‘various’ artery	433.x	Occlusion and stenosis of precerebral arteries
I66.x	Occlusion and stenosis of ‘various’ cerebral artery	434.x	Occlusion of cerebral arteries
G45.x	Other transient cerebral ischemic attacks and related syndromes	435.x	Transient cerebral ischemia
I67.89	Other cerebrovascular disease	436.x	Acute, but ill-defined, cerebrovascular disease
I67.x	Cerebral atherosclerosis, or Cerebrovascular disease, unspecified	437.x	Other and ill-defined cerebrovascular disease
I69.x	Unspecified sequelae of unspecified cerebrovascular disease	438.x	Late effects of cerebrovascular disease

Table S2d. ICD-9 and ICD-10 Codes for Cancer

ICD-10 Codes	Description	ICD-9 Codes	Description
C00	Malignant neoplasm of lip	140	MALIGNANT NEOPLASM LIP
C000	MALIGNANT NEOPLASM OF EXTERNAL UPPER LIP	1400	MAL NEO UPPER VERMILION
C001	MALIGNANT NEOPLASM OF EXTERNAL LOWER LIP	1401	MAL NEO LOWER VERMILION
C003	MALIGNANT NEOPLASM OF UPPER LIP, INNER ASPECT	1403	MAL NEO UPPER LIP, INNER
C004	MALIGNANT NEOPLASM OF LOWER LIP, INNER ASPECT	1404	MAL NEO LOWER LIP, INNER
C005	MALIGNANT NEOPLASM OF LIP, UNSPECIFIED, INNER ASPECT	1405	MAL NEO LIP, INNER NOS
C006	MALIGNANT NEOPLASM OF COMMISSURE OF LIP, UNSPECIFIED	1406	MAL NEO LIP, COMMISSURE
C008	MALIGNANT NEOPLASM OF OVERLAPPING SITES OF LIP	1408	MAL NEO LIP NEC
C002	MALIGNANT NEOPLASM OF EXTERNAL LIP, UNSPECIFIED	1409	MAL NEO LIP/VERMIL NOS
C029	MALIGNANT NEOPLASM OF TONGUE, UNSPECIFIED	141	MALIG NEO TONGUE
C01	MALIGNANT NEOPLASM OF BASE OF TONGUE	1410	MAL NEO TONGUE BASE
C020	MALIGNANT NEOPLASM OF DORSAL SURFACE OF TONGUE	1411	MAL NEO DORSAL TONGUE
C021	MALIGNANT NEOPLASM OF BORDER OF TONGUE	1412	MAL NEO TIP/LAT TONGUE
C022	MALIGNANT NEOPLASM OF VENTRAL SURFACE OF TONGUE	1413	MAL NEO VENTRAL TONGUE
C023	MALIGNANT NEOPLASM OF ANTERIOR TWO-THIRDS OF TONGUE, PART UNSPECIFIED	1414	MAL NEO ANT 2/3 TONGUE
C028	MALIGNANT NEOPLASM OF OVERLAPPING SITES OF TONGUE	1415	MAL NEO TONGUE JUNCTION
C024	MALIGNANT NEOPLASM OF LINGUAL TONSIL	1416	MAL NEO LINGUAL TONSIL
C028	MALIGNANT NEOPLASM OF OVERLAPPING SITES OF TONGUE	1418	MALIG NEO TONGUE NEC
C029	MALIGNANT NEOPLASM OF TONGUE, UNSPECIFIED	1419	MALIG NEO TONGUE NOS
C089	MALIGNANT NEOPLASM OF MAJOR SALIVARY GLAND, UNSPECIFIED	142	MAL NEO MAJOR SALIVARY
C07	MALIGNANT NEOPLASM OF PAROTID GLAND	1420	MALIG NEO PAROTID
C080	MALIGNANT NEOPLASM OF SUBMANDIBULAR GLAND	1421	MALIG NEO SUBMANDIBULAR
C081	MALIGNANT NEOPLASM OF SUBLINGUAL GLAND	1422	MALIG NEO SUBLINGUAL
C088	MALIGNANT NEOPLASM OF MAJOR SALIVARY GLAND, UNSPECIFIED	1428	MAL NEO MAJ SALIVARY NEC
C089	MALIGNANT NEOPLASM OF MAJOR SALIVARY GLAND, UNSPECIFIED	1429	MAL NEO SALIVARY NOS
C03	Malignant neoplasm of gum	143	MALIGNANT NEOPLASM GUM
C030	MALIGNANT NEOPLASM OF UPPER GUM	1430	MALIG NEO UPPER GUM
C031	MALIGNANT NEOPLASM OF LOWER GUM	1431	MALIG NEO LOWER GUM
C039	MALIGNANT NEOPLASM OF GUM, UNSPECIFIED	1438	MALIG NEO GUM NEC
C039	MALIGNANT NEOPLASM OF GUM, UNSPECIFIED	1439	MALIG NEO GUM NOS
C04	Malignant neoplasm of floor of mouth	144	MALIG NEO MOUTH FLOOR
C040	MALIGNANT NEOPLASM OF ANTERIOR FLOOR OF MOUTH	1440	MAL NEO ANT FLOOR MOUTH
C041	MALIGNANT NEOPLASM OF LATERAL FLOOR OF MOUTH	1441	MAL NEO LAT FLOOR MOUTH
C048	MALIGNANT NEOPLASM OF OVERLAPPING SITES OF FLOOR OF MOUTH	1448	MAL NEO MOUTH FLOOR NEC
C049	MALIGNANT NEOPLASM OF FLOOR OF MOUTH, UNSPECIFIED	1449	MAL NEO MOUTH FLOOR NOS
C06	Malignant neoplasm of other and unspecified parts of mouth	145	MALIG NEO MOUTH NEC/NOS
C060	MALIGNANT NEOPLASM OF CHEEK MUCOSA	1450	MAL NEO CHEEK MUCOSA
C061	MALIGNANT NEOPLASM OF VESTIBULE OF MOUTH	1451	MAL NEO MOUTH VESTIBULE

C050	MALIGNANT NEOPLASM OF HARD PALATE	1452	MALIG NEO HARD PALATE
C051	MALIGNANT NEOPLASM OF SOFT PALATE	1453	MALIG NEO SOFT PALATE
C052	MALIGNANT NEOPLASM OF UVULA	1454	MALIGNANT NEOPLASM UVULA
C059	MALIGNANT NEOPLASM OF PALATE, UNSPECIFIED	1455	MALIGNANT NEO PALATE NOS
C062	MALIGNANT NEOPLASM OF RETROMOLAR AREA	1456	MALIG NEO RETROMOLAR
C0689	MALIGNANT NEOPLASM OF OVERLAPPING SITES OF OTHER PARTS OF MOUTH	1458	MALIG NEOPLASM MOUTH NEC
C069	MALIGNANT NEOPLASM OF MOUTH, UNSPECIFIED	1459	MALIG NEOPLASM MOUTH NOS
C10	Malignant neoplasm of oropharynx	146	MALIG NEO OROPHARYNX
C099	MALIGNANT NEOPLASM OF TONSIL, UNSPECIFIED	1460	MALIGNANT NEOPL TONSIL
C090	MALIGNANT NEOPLASM OF TONSILLAR FOSSA	1461	MAL NEO TONSILLAR FOSSA
C091	MALIGNANT NEOPLASM OF TONSILLAR PILLAR (ANTERIOR) (POSTERIOR)	1462	MAL NEO TONSIL PILLARS
C100	MALIGNANT NEOPLASM OF VALLECULA	1463	MALIGN NEOPL VALLECULA
C101	MALIGNANT NEOPLASM OF ANTERIOR SURFACE OF EPIGLOTTIS	1464	MAL NEO ANT EPIGLOTTIS
C108	MALIGNANT NEOPLASM OF OVERLAPPING SITES OF OROPHARYNX	1465	MAL NEO EPIGLOTTIS JUNCT
C102	MALIGNANT NEOPLASM OF LATERAL WALL OF OROPHARYNX	1466	MAL NEO LAT OROPHARYNX
C103	MALIGNANT NEOPLASM OF POSTERIOR WALL OF OROPHARYNX	1467	MAL NEO POST OROPHARYNX
C109	MALIGNANT NEOPLASM OF OROPHARYNX, UNSPECIFIED	1468	MAL NEO OROPHARYNX NEC
C109	MALIGNANT NEOPLASM OF OROPHARYNX, UNSPECIFIED	1469	MALIG NEO OROPHARYNX NOS
C11	Malignant neoplasm of nasopharynx	147	MALIG NEO NASOPHARYNX
C110	MALIGNANT NEOPLASM OF SUPERIOR WALL OF NASOPHARYNX	1470	MAL NEO SUPER NASOPHARYN
C111	MALIGNANT NEOPLASM OF POSTERIOR WALL OF NASOPHARYNX	1471	MAL NEO POST NASOPHARYNX
C112	MALIGNANT NEOPLASM OF LATERAL WALL OF NASOPHARYNX	1472	MAL NEO LAT NASOPHARYNX
C113	MALIGNANT NEOPLASM OF ANTERIOR WALL OF NASOPHARYNX	1473	MAL NEO ANT NASOPHARYNX
C118	MALIGNANT NEOPLASM OF OVERLAPPING SITES OF NASOPHARYNX	1478	MAL NEO NASOPHARYNX NEC
C119	MALIGNANT NEOPLASM OF NASOPHARYNX, UNSPECIFIED	1479	MAL NEO NASOPHARYNX NOS
C13	Malignant neoplasm of hypopharynx	148	MALIG NEOPL HYPOPHARYNX
C130	MALIGNANT NEOPLASM OF POSTCRICOID REGION	1480	MAL NEO POSTCRICOID
C12	MALIGNANT NEOPLASM OF PYRIFORM SINUS	1481	MAL NEO PYRIFORM SINUS
C131	MALIGNANT NEOPLASM OF ARYEPIGLOTTIC FOLD, HYPOPHARYNGEAL ASPECT	1482	MAL NEO ARYEPIGLOTT FOLD
C132	MALIGNANT NEOPLASM OF POSTERIOR WALL OF HYPOPHARYNX	1483	MAL NEO POST HYPOPHARYNX
C138	MALIGNANT NEOPLASM OF OVERLAPPING SITES OF HYPOPHARYNX	1488	MAL NEO HYPOPHARYNX NEC
C139	MALIGNANT NEOPLASM OF HYPOPHARYNX, UNSPECIFIED	1489	MAL NEO HYPOPHARYNX NOS
C14	Malignant neoplasm of other and ill-defined sites in the lip, oral cavity, and pharynx	149	OTH MALIG NEO OROPHARYNX
C140	MALIGNANT NEOPLASM OF PHARYNX, UNSPECIFIED	1490	MAL NEO PHARYNX NOS
C142	MALIGNANT NEOPLASM OF WALDEYER'S RING	1491	MAL NEO WALDEYER'S RING
C148	MALIGNANT NEOPLASM OF OVERLAPPING SITES OF LIP, ORAL CAVITY AND PHARYNX	1498	MAL NEO ORAL/PHARYNX NEC
C148	MALIGNANT NEOPLASM OF OVERLAPPING SITES OF LIP, ORAL CAVITY AND PHARYNX	1499	MAL NEO OROPHRYN ILL-DEF
C15	Malignant neoplasm of esophagus	150	MALIGNANT NEO ESOPHAGUS
C150	MALIGNANT NEOPLASM OF UPPER THIRD OF ESOPHAGUS	1500	MAL NEO CERVICAL ESOPHAG
C151	MALIGNANT NEOPLASM OF MIDDLE THIRD OF ESOPHAGUS	1501	MAL NEO THORACIC ESOPHAG
C152	MALIGNANT NEOPLASM OF LOWER THIRD OF ESOPHAGUS	1502	MAL NEO ABDOMIN ESOPHAG
C153	MALIGNANT NEOPLASM OF UPPER THIRD OF ESOPHAGUS	1503	MAL NEO UPPER 3RD ESOPH
C154	MALIGNANT NEOPLASM OF MIDDLE THIRD OF ESOPHAGUS	1504	MAL NEO MIDDLE 3RD ESOPH
C155	MALIGNANT NEOPLASM OF LOWER THIRD OF ESOPHAGUS	1505	MAL NEO LOWER 3RD ESOPH

C158	MALIGNANT NEOPLASM OF OVERLAPPING SITES OF ESOPHAGUS	1508	MAL NEO ESOPHAGUS NEC
C159	MALIGNANT NEOPLASM OF ESOPHAGUS, UNSPECIFIED	1509	MAL NEO ESOPHAGUS NOS
C16	Malignant neoplasm of stomach	151	MALIGNANT NEO STOMACH
C160	MALIGNANT NEOPLASM OF CARDIA	1510	MAL NEO STOMACH CARDIA
C164	MALIGNANT NEOPLASM OF PYLORUS	1511	MALIGNANT NEO PYLORUS
C163	MALIGNANT NEOPLASM OF PYLORIC ANTRUM	1512	MAL NEO PYLORIC ANTRUM
C161	MALIGNANT NEOPLASM OF FUNDUS OF STOMACH	1513	MAL NEO STOMACH FUNDUS
C162	MALIGNANT NEOPLASM OF BODY OF STOMACH	1514	MAL NEO STOMACH BODY
C165	MALIGNANT NEOPLASM OF LESSER CURVATURE OF STOMACH, UNSPECIFIED	1515	MAL NEO STOM LESSER CURV
C166	MALIGNANT NEOPLASM OF GREATER CURVATURE OF STOMACH, UNSPECIFIED	1516	MAL NEO STOM GREAT CURV
C168	MALIGNANT NEOPLASM OF OVERLAPPING SITES OF STOMACH	1518	MALIG NEOPL STOMACH NEC
C169	MALIGNANT NEOPLASM OF STOMACH, UNSPECIFIED	1519	MALIG NEOPL STOMACH NOS
C17	Malignant neoplasm of small intestine	152	MALIG NEO SMALL BOWEL
C170	MALIGNANT NEOPLASM OF DUODENUM	1520	MALIGNANT NEOPL DUODENUM
C171	MALIGNANT NEOPLASM OF JEJUNUM	1521	MALIGNANT NEOPL JEJUNUM
C172	MALIGNANT NEOPLASM OF ILEUM	1522	MALIGNANT NEOPLASM ILEUM
C173	MECKEL'S DIVERTICULUM, MALIGNANT	1523	MAL NEO MECKEL'S DIVERT
C178	MALIGNANT NEOPLASM OF OVERLAPPING SITES OF SMALL INTESTINE	1528	MAL NEO SMALL BOWEL NEC
C179	MALIGNANT NEOPLASM OF SMALL INTESTINE, UNSPECIFIED	1529	MAL NEO SMALL BOWEL NOS
C18	Malignant neoplasm of colon	153	MALIGNANT NEOPLASM COLON
C183	MALIGNANT NEOPLASM OF HEPATIC FLEXURE	1530	MAL NEO HEPATIC FLEXURE
C184	MALIGNANT NEOPLASM OF TRANSVERSE COLON	1531	MAL NEO TRANSVERSE COLON
C186	MALIGNANT NEOPLASM OF DESCENDING COLON	1532	MAL NEO DESCEND COLON
C187	MALIGNANT NEOPLASM OF SIGMOID COLON	1533	MAL NEO SIGMOID COLON
C180	MALIGNANT NEOPLASM OF CECUM	1534	MALIGNANT NEOPLASM CECUM
C181	MALIGNANT NEOPLASM OF APPENDIX	1535	MALIGNANT NEO APPENDIX
C182	MALIGNANT NEOPLASM OF ASCENDING COLON	1536	MALIG NEO ASCEND COLON
C185	MALIGNANT NEOPLASM OF SPLENIC FLEXURE	1537	MAL NEO SPLENIC FLEXURE
C188	MALIGNANT NEOPLASM OF OVERLAPPING SITES OF COLON	1538	MALIGNANT NEO COLON NEC
C189	MALIGNANT NEOPLASM OF COLON, UNSPECIFIED	1539	MALIGNANT NEO COLON NOS
C218	MALIGNANT NEOPLASM OF OVERLAPPING SITES OF RECTUM, ANUS AND ANAL CANAL	154	MALIG NEO RECTUM/ANUS
C19	MALIGNANT NEOPLASM OF RECTOSIGMOID JUNCTION	1540	MAL NEO RECTOSIGMOID JCT
C20	MALIGNANT NEOPLASM OF RECTUM	1541	MALIGNANT NEOPL RECTUM
C211	MALIGNANT NEOPLASM OF ANAL CANAL	1542	MALIG NEOPL ANAL CANAL
C210	MALIGNANT NEOPLASM OF ANUS, UNSPECIFIED	1543	MALIGNANT NEO ANUS NOS
C218	MALIGNANT NEOPLASM OF OVERLAPPING SITES OF RECTUM, ANUS AND ANAL CANAL	1548	MAL NEO RECTUM/ANUS NEC
C22	Malignant neoplasm of liver and intrahepatic bile ducts	155	MALIGNANT NEOPLASM LIVER
C228	MALIGNANT NEOPLASM OF LIVER, PRIMARY, UNSPECIFIED AS TO TYPE	1550	MAL NEO LIVER, PRIMARY
C221	INTRAHEPATIC BILE DUCT CARCINOMA	1551	MAL NEO INTRAHEPAT DUCTS
C229	MALIGNANT NEOPLASM OF LIVER, NOT SPECIFIED AS PRIMARY OR SECONDARY	1552	MALIGNANT NEO LIVER NOS
C248	MALIGNANT NEOPLASM OF OVERLAPPING SITES OF BILIARY TRACT	156	MAL NEO GB/EXTRAHEPATIC
C23	MALIGNANT NEOPLASM OF GALLBLADDER	1560	MALIG NEO GALLBLADDER
C240	MALIGNANT NEOPLASM OF EXTRAHEPATIC BILE DUCT	1561	MAL NEO EXTRAHEPAT DUCTS
C241	MALIGNANT NEOPLASM OF AMPULLA OF VATER	1562	MAL NEO AMPULLA OF VATER

C248	MALIGNANT NEOPLASM OF OVERLAPPING SITES OF BILIARY TRACT	1568	MALIG NEO BILIARY NEC
C249	MALIGNANT NEOPLASM OF BILIARY TRACT, UNSPECIFIED	1569	MALIG NEO BILIARY NOS
C25	Malignant neoplasm of pancreas	157	MALIGNANT NEO PANCREAS
C250	MALIGNANT NEOPLASM OF HEAD OF PANCREAS	1570	MAL NEO PANCREAS HEAD
C251	MALIGNANT NEOPLASM OF BODY OF PANCREAS	1571	MAL NEO PANCREAS BODY
C252	MALIGNANT NEOPLASM OF TAIL OF PANCREAS	1572	MAL NEO PANCREAS TAIL
C253	MALIGNANT NEOPLASM OF PANCREATIC DUCT	1573	MAL NEO PANCREATIC DUCT
C254	MALIGNANT NEOPLASM OF ENDOCRINE PANCREAS	1574	MAL NEO ISLET LANGERHANS
C257	MALIGNANT NEOPLASM OF OTHER PARTS OF PANCREAS	1578	MALIG NEO PANCREAS NEC
C259	MALIGNANT NEOPLASM OF PANCREAS, UNSPECIFIED	1579	MALIG NEO PANCREAS NOS
C48	Malignant neoplasm of retroperitoneum and peritoneum	158	MALIG NEO PERITONEUM
C480	MALIGNANT NEOPLASM OF RETROPERITONEUM	1580	MAL NEO RETROPERITONEUM
C481	MALIGNANT NEOPLASM OF SPECIFIED PARTS OF PERITONEUM	1588	MAL NEO PERITONEUM NEC
C482	MALIGNANT NEOPLASM OF PERITONEUM, UNSPECIFIED	1589	MAL NEO PERITONEUM NOS
C26	Malignant neoplasm of other and ill-defined digestive organs	159	OTH MALIG NEO GI/PERITON
C260	MALIGNANT NEOPLASM OF INTESTINAL TRACT, PART UNSPECIFIED	1590	MALIG NEO INTESTINE NOS
C261	MALIGNANT NEOPLASM OF SPLEEN	1591	MALIGNANT NEO SPLEEN NEC
C269	MALIGNANT NEOPLASM OF ILL-DEFINED SITES WITHIN THE DIGESTIVE SYSTEM	1598	MAL NEO GI/INTRA-ABD NEC
C269	MALIGNANT NEOPLASM OF ILL-DEFINED SITES WITHIN THE DIGESTIVE SYSTEM	1599	MAL NEO GI TRACT ILL-DEF
C30	Malignant neoplasm of respiratory and intrathoracic organs	160	MAL NEO NASAL CAV/SINUS
C300	MALIGNANT NEOPLASM OF NASAL CAVITY	1600	MAL NEO NASAL CAVITIES
C301	MALIGNANT NEOPLASM OF MIDDLE EAR	1601	MALIG NEO MIDDLE EAR
C310	MALIGNANT NEOPLASM OF MAXILLARY SINUS	1602	MAL NEO MAXILLARY SINUS
C311	MALIGNANT NEOPLASM OF ETHMOIDAL SINUS	1603	MAL NEO ETHMOIDAL SINUS
C312	MALIGNANT NEOPLASM OF FRONTAL SINUS	1604	MALIG NEO FRONTAL SINUS
C313	MALIGNANT NEOPLASM OF SPHENOID SINUS	1605	MAL NEO SPHENOID SINUS
C318	MALIGNANT NEOPLASM OF OVERLAPPING SITES OF ACCESSORY SINUSES	1608	MAL NEO ACCESS SINUS NEC
C319	MALIGNANT NEOPLASM OF ACCESSORY SINUS, UNSPECIFIED	1609	MAL NEO ACCESS SINUS NOS
C32	Malignant neoplasm of larynx	161	MALIGNANT NEO LARYNX
C320	MALIGNANT NEOPLASM OF GLOTTIS	1610	MALIGNANT NEO GLOTTIS
C321	MALIGNANT NEOPLASM OF SUPRAGLOTTIS	1611	MALIG NEO SUPRAGLOTTIS
C322	MALIGNANT NEOPLASM OF SUBGLOTTIS	1612	MALIG NEO SUBGLOTTIS
C323	MALIGNANT NEOPLASM OF LARYNGEAL CARTILAGE	1613	MAL NEO CARTILAGE LARYNX
C328	MALIGNANT NEOPLASM OF OVERLAPPING SITES OF LARYNX	1618	MALIGNANT NEO LARYNX NEC
C329	MALIGNANT NEOPLASM OF LARYNX, UNSPECIFIED	1619	MALIGNANT NEO LARYNX NOS
C34	Malignant neoplasm of trachea and lung	162	MAL NEO TRACHEA/LUNG*
C33	Malignant neoplasm of trachea	1620	MALIGNANT NEO TRACHEA
C340	Malignant neoplasm of main bronchus	1622	MALIG NEO MAIN BRONCHUS
C341	Malignant neoplasm of upper lobe, bronchus or lung	1623	MAL NEO UPPER LOBE LUNG
C342	Malignant neoplasm of middle lobe, bronchus or lung	1624	MAL NEO MIDDLE LOBE LUNG
C343	Malignant neoplasm of lower lobe, bronchus or lung	1625	MAL NEO LOWER LOBE LUNG
C348	Malignant neoplasm of overlapping sites of bronchus and lung	1628	MAL NEO BRONCH/LUNG NEC
C349	Malignant neoplasm of unspecified part of bronchus or lung	1629	MAL NEO BRONCH/LUNG NOS
C384	MALIGNANT NEOPLASM OF PLEURA	163	MALIGNANT NEOPL PLEURA

C384	MALIGNANT NEOPLASM OF PLEURA	1630	MAL NEO PARIETAL PLEURA
C384	MALIGNANT NEOPLASM OF PLEURA	1631	MAL NEO VISCERAL PLEURA
C384	MALIGNANT NEOPLASM OF PLEURA	1638	MALIG NEOPL PLEURA NEC
C384	MALIGNANT NEOPLASM OF PLEURA	1639	MALIG NEOPL PLEURA NOS
C457	MALIGNANT NEOPLASM OF MEDIASTINUM, PART UNSPECIFIED	164	MAL NEO THYMUS/MEDIASTIN
C37	Malignant neoplasm of thymus	1640	MALIGNANT NEOPL THYMUS
C380	MALIGNANT NEOPLASM OF HEART	1641	MALIGNANT NEOPL HEART
C381	MALIGNANT NEOPLASM OF ANTERIOR MEDIASTINUM	1642	MAL NEO ANT MEDIASTINUM
C382	MALIGNANT NEOPLASM OF POSTERIOR MEDIASTINUM	1643	MAL NEO POST MEDIASTINUM
C388	MALIGNANT NEOPLASM OF OVERLAPPING SITES OF HEART, MEDIASTINUM AND PLEURA	1648	MAL NEO MEDIASTINUM NEC
C457	MALIGNANT NEOPLASM OF MEDIASTINUM, PART UNSPECIFIED	1649	MAL NEO MEDIASTINUM NOS
C39	Malignant neoplasm of other and ill-defined sited in the respiratory system and intrathoracic organs	165	OTH/ILL-DEF MAL NEO RESP
C390	MALIGNANT NEOPLASM OF UPPER RESPIRATORY TRACT, PART UNSPECIFIED	1650	MAL NEO UPPER RESP NOS
C399	MALIGNANT NEOPLASM OF LOWER RESPIRATORY TRACT, PART UNSPECIFIED	1658	MAL NEO THORAX/RESP NEC
C399	MALIGNANT NEOPLASM OF LOWER RESPIRATORY TRACT, PART UNSPECIFIED	1659	MAL NEO RESP SYSTEM NOS
C41	Malignant neoplasm of bone and articular cartilage of other and unspecified sites	170	MAL NEO BONE/ARTIC CART
C410	MALIGNANT NEOPLASM OF BONES OF SKULL AND FACE	1700	MAL NEO SKULL/FACE BONE
C411	MALIGNANT NEOPLASM OF MANDIBLE	1701	MALIGNANT NEO MANDIBLE
C412	MALIGNANT NEOPLASM OF VERTEBRAL COLUMN	1702	MALIG NEO VERTEBRAE
C413	MALIGNANT NEOPLASM OF RIBS, STERNUM AND CLAVICLE	1703	MAL NEO RIBS/STERN/CLAV
C4000	MALIGNANT NEOPLASM OF SCAPULA AND LONG BONES OF UNSPECIFIED UPPER LIMB	1704	MAL NEO LONG BONES ARM
C4010	MALIGNANT NEOPLASM OF SHORT BONES OF UNSPECIFIED UPPER LIMB	1705	MAL NEO BONES WRIST/HAND
C414	MALIGNANT NEOPLASM OF PELVIC BONES, SACRUM AND COCCYX	1706	MAL NEO PELVIC GIRDLE
C4020	MALIGNANT NEOPLASM OF LONG BONES OF UNSPECIFIED LOWER LIMB	1707	MAL NEO LONG BONES LEG
C4030	MALIGNANT NEOPLASM OF SHORT BONES OF UNSPECIFIED LOWER LIMB	1708	MAL NEO BONES ANKLE/FOOT
C419	MALIGNANT NEOPLASM OF BONE AND ARTICULAR CARTILAGE, UNSPECIFIED	1709	MALIG NEOPL BONE NOS
C49	Malignant neoplasm of other connective and soft tissue	171	MAL NEO SOFT TISSUE
C490	MALIGNANT NEOPLASM OF CONNECTIVE AND SOFT TISSUE OF HEAD, FACE AND NECK	1710	MAL NEO SOFT TISSUE HEAD
C4910	MALIGNANT NEOPLASM OF CONNECTIVE AND SOFT TISSUE OF UNSPECIFIED UPPER LIMB, INCLUDING SHOULDER	1712	MAL NEO SOFT TISSUE ARM
C4920	MALIGNANT NEOPLASM OF CONNECTIVE AND SOFT TISSUE OF UNSPECIFIED LOWER LIMB, INCLUDING HIP	1713	MAL NEO SOFT TISSUE LEG
C493	MALIGNANT NEOPLASM OF CONNECTIVE AND SOFT TISSUE OF THORAX	1714	MAL NEO SOFT TIS THORAX
C494	MALIGNANT NEOPLASM OF CONNECTIVE AND SOFT TISSUE OF ABDOMEN	1715	MAL NEO SOFT TIS ABDOMEN
C495	MALIGNANT NEOPLASM OF CONNECTIVE AND SOFT TISSUE OF PELVIS	1716	MAL NEO SOFT TIS PELVIS
C496	MALIGNANT NEOPLASM OF CONNECTIVE AND SOFT TISSUE OF TRUNK, UNSPECIFIED	1717	MAL NEOPL TRUNK NOS
C498	MALIGNANT NEOPLASM OF OVERLAPPING SITES OF CONNECTIVE AND SOFT TISSUE	1718	MAL NEO SOFT TISSUE NEC
C499	MALIGNANT NEOPLASM OF CONNECTIVE AND SOFT TISSUE, UNSPECIFIED	1719	MAL NEO SOFT TISSUE NOS
C43	Malignant melanoma of skin	172	MALIGNANT MELANOMA SKIN
C430	MALIGNANT MELANOMA OF LIP	1720	MALIG MELANOMA LIP
C4310	MALIGNANT MELANOMA OF UNSPECIFIED EYELID, INCLUDING CANTHUS	1721	MALIG MELANOMA EYELID
C4320	MALIGNANT MELANOMA OF UNSPECIFIED EAR AND EXTERNAL AURICULAR CANAL	1722	MALIG MELANOMA EAR
C4330	MALIGNANT MELANOMA OF UNSPECIFIED PART OF FACE	1723	MAL MELANOM FACE NEC/NOS
C434	MALIGNANT MELANOMA OF SCALP AND NECK	1724	MAL MELANOMA SCALP/NECK

C435	Malignant melanoma of trunk	1725	MALIG MELANOMA TRUNK
C4360	MALIGNANT MELANOMA OF UNSPECIFIED UPPER LIMB, INCLUDING SHOULDER	1726	MALIG MELANOMA ARM
C4370	MALIGNANT MELANOMA OF UNSPECIFIED LOWER LIMB, INCLUDING HIP	1727	MALIG MELANOMA LEG
C438	MALIGNANT MELANOMA OF OVERLAPPING SITES OF SKIN	1728	MALIG MELANOMA SKIN NEC
C439	MALIGNANT MELANOMA OF SKIN, UNSPECIFIED	1729	MALIG MELANOMA SKIN NOS
C5091	Malignant neoplasm of breast of unspecified site, female	174	MALIG NEO FEMALE BREAST
C500	Malignant neoplasm of nipple and areola	1740	MALIG NEO NIPPLE
C50119	MALIGNANT NEOPLASM OF CENTRAL PORTION OF UNSPECIFIED FEMALE BREAST	1741	MAL NEO BREAST-CENTRAL
C50219	MALIGNANT NEOPLASM OF UPPER-INNER QUADRANT OF UNSPECIFIED FEMALE BREAST	1742	MAL NEO BREAST UP-INNER
C50319	MALIGNANT NEOPLASM OF LOWER-INNER QUADRANT OF UNSPECIFIED FEMALE BREAST	1743	MAL NEO BREAST LOW-INNER
C50419	MALIGNANT NEOPLASM OF UPPER-OUTER QUADRANT OF UNSPECIFIED FEMALE BREAST	1744	MAL NEO BREAST UP-OUTER
C50519	MALIGNANT NEOPLASM OF LOWER-OUTER QUADRANT OF UNSPECIFIED FEMALE BREAST	1745	MAL NEO BREAST LOW-OUTER
C50619	MALIGNANT NEOPLASM OF AXILLARY TAIL OF UNSPECIFIED FEMALE BREAST	1746	MAL NEO BREAST-AXILLARY
C50819	MALIGNANT NEOPLASM OF OVERLAPPING SITES OF UNSPECIFIED FEMALE BREAST	1748	MALIGN NEOPL BREAST NEC
C50919	MALIGNANT NEOPLASM OF UNSPECIFIED SITE OF UNSPECIFIED FEMALE BREAST	1749	MALIGN NEOPL BREAST NOS
C50929	MALIGNANT NEOPLASM OF UNSPECIFIED SITE OF UNSPECIFIED MALE BREAST	175	MALIG NEO MALE BREAST
C50929	MALIGNANT NEOPLASM OF UNSPECIFIED SITE OF UNSPECIFIED MALE BREAST	1759	MAL NEO MALE BREAST NEC
C46	NULL	176	Kaposi's sarcoma
C460	KAPOSI'S SARCOMA OF SKIN	1760	SKIN - KAPOSI'S SARCOMA
C461	KAPOSI'S SARCOMA OF SOFT TISSUE	1761	SFT TISUE - KPSI'S SRCMA
C462	KAPOSI'S SARCOMA OF PALATE	1762	PALATE - KPSI's SARCOMA
C464	KAPOSI'S SARCOMA OF GASTROINTESTINAL SITES	1763	GI SITES - KPSI'S SRCOMA
C4650	KAPOSI'S SARCOMA OF UNSPECIFIED LUNG	1764	LUNG - KAPOSI'S SARCOMA
C463	KAPOSI'S SARCOMA OF LYMPH NODES	1765	LYM NDS - KPSI'S SARCOMA
C467	KAPOSI'S SARCOMA OF OTHER SITES	1768	SPF STS - KPSI'S SARCOMA
C469	KAPOSI'S SARCOMA, UNSPECIFIED	1769	KAPOSI'S SARCOMA NOS
C55	MALIGNANT NEOPLASM OF UTERUS, PART UNSPECIFIED	179	MALIG NEOPL UTERUS NOS
C53	Malignant neoplasm of cervix uteri	180	MALIG NEOPL CERVIX UTERI*
C530	MALIGNANT NEOPLASM OF ENDOCERVIX	1800	MALIG NEO ENDOCERVIX
C531	MALIGNANT NEOPLASM OF EXOCERVIX	1801	MALIG NEO EXOCERVIX
C538	MALIGNANT NEOPLASM OF OVERLAPPING SITES OF CERVIX UTERI	1808	MALIG NEO CERVIX NEC
C539	MALIGNANT NEOPLASM OF CERVIX UTERI, UNSPECIFIED	1809	MAL NEO CERVIX UTERI NOS
C574	MALIGNANT NEOPLASM OF UTERINE ADNEXA, UNSPECIFIED	183	MAL NEO UTERINE ADNEXA*
C569	MALIGNANT NEOPLASM OF UNSPECIFIED OVARY	1830	MALIGN NEOPL OVARY
C5700	MALIGNANT NEOPLASM OF UNSPECIFIED FALLOPIAN TUBE	1832	MAL NEO FALLOPIAN TUBE
C5710	MALIGNANT NEOPLASM OF UNSPECIFIED BROAD LIGAMENT	1833	MAL NEO BROAD LIGAMENT
C573	MALIGNANT NEOPLASM OF PARAMETRIUM	1834	MALIG NEO PARAMETRIUM
C5720	MALIGNANT NEOPLASM OF UNSPECIFIED ROUND LIGAMENT	1835	MAL NEO ROUND LIGAMENT
C577	MALIGNANT NEOPLASM OF UTERINE ADNEXA, UNSPECIFIED	1838	MAL NEO ADNEXA NEC
C574	MALIGNANT NEOPLASM OF UTERINE ADNEXA, UNSPECIFIED	1839	MAL NEO ADNEXA NOS
C57	Malignant neoplasm of other and unspecified female genital organs	184	MAL NEO FEM GEN NEC/NOS*
C52	MALIGNANT NEOPLASM OF VAGINA	1840	MALIGN NEOPL VAGINA
C510	MALIGNANT NEOPLASM OF LABIUM MAJUS	1841	MAL NEO LABIA MAJORA
C511	MALIGNANT NEOPLASM OF LABIUM MINUS	1842	MAL NEO LABIA MINORA

C512	MALIGNANT NEOPLASM OF CLITORIS	1843	MALIGN NEOPL CLITORIS
C519	MALIGNANT NEOPLASM OF VULVA, UNSPECIFIED	1844	MALIGN NEOPL VULVA NOS
C577	MALIGNANT NEOPLASM OF OTHER SPECIFIED FEMALE GENITAL ORGANS	1848	MAL NEO FEMALE GENIT NEC
C579	MALIGNANT NEOPLASM OF FEMALE GENITAL ORGAN, UNSPECIFIED	1849	MAL NEO FEMALE GENIT NOS
C61	MALIGNANT NEOPLASM OF PROSTATE	185	MALIGN NEOPL PROSTATE
C62	Malignant neoplasm of testis	186	MALIGN NEOPL TESTIS*
C6200	MALIGNANT NEOPLASM OF UNSPECIFIED UNDESCENDED TESTIS	1860	MAL NEO UNDESCEND TESTIS
C6290	MALIGNANT NEOPLASM OF UNSPECIFIED TESTIS, UNSPECIFIED WHETHER DESCENDED OR UNDESCENDED	1869	MALIG NEO TESTIS NEC
C639	MALIGNANT NEOPLASM OF MALE GENITAL ORGAN, UNSPECIFIED	187	MAL NEO MALE GENITAL NEC*
C600	MALIGNANT NEOPLASM OF PREPUCE	1871	MALIGN NEOPL PREPUCE
C601	MALIGNANT NEOPLASM OF GLANS PENIS	1872	MALIG NEO GLANS PENIS
C602	MALIGNANT NEOPLASM OF BODY OF PENIS	1873	MALIG NEO PENIS BODY
C609	MALIGNANT NEOPLASM OF PENIS, UNSPECIFIED	1874	MALIG NEO PENIS NOS
C6300	MALIGNANT NEOPLASM OF UNSPECIFIED EPIDIDYMIS	1875	MALIG NEO EPIDIDYMIS
C6310	MALIGNANT NEOPLASM OF UNSPECIFIED SPERMATIC CORD	1876	MAL NEO SPERMATIC CORD
C632	MALIGNANT NEOPLASM OF SCROTUM	1877	MALIGN NEOPL SCROTUM
C637	MALIGNANT NEOPLASM OF OTHER SPECIFIED MALE GENITAL ORGANS	1878	MAL NEO MALE GENITAL NEC
C639	MALIGNANT NEOPLASM OF MALE GENITAL ORGAN, UNSPECIFIED	1879	MAL NEO MALE GENITAL NOS
C67	MALIGNANT NEOPLASM OF BLADDER	188	MALIGN NEOPL BLADDER*
C670	MALIGNANT NEOPLASM OF TRIGONE OF BLADDER	1880	MAL NEO BLADDER-TRIGONE
C671	MALIGNANT NEOPLASM OF DOME OF BLADDER	1881	MAL NEO BLADDER-DOME
C672	MALIGNANT NEOPLASM OF LATERAL WALL OF BLADDER	1882	MAL NEO BLADDER-LATERAL
C673	MALIGNANT NEOPLASM OF ANTERIOR WALL OF BLADDER	1883	MAL NEO BLADDER-ANTERIOR
C674	MALIGNANT NEOPLASM OF POSTERIOR WALL OF BLADDER	1884	MAL NEO BLADDER-POST
C675	MALIGNANT NEOPLASM OF BLADDER NECK	1885	MAL NEO BLADDER NECK
C676	MALIGNANT NEOPLASM OF URETERIC ORIFICE	1886	MAL NEO URETERIC ORIFICE
C677	MALIGNANT NEOPLASM OF URACHUS	1887	MALIG NEO URACHUS
C678	MALIGNANT NEOPLASM OF OVERLAPPING SITES OF BLADDER	1888	MALIG NEO BLADDER NEC
C679	MALIGNANT NEOPLASM OF BLADDER, UNSPECIFIED	1889	MALIG NEO BLADDER NOS
C68	Malignant neoplasm of other and unspecified urinary organs	189	MAL NEO URINARY NEC/NOS*
C64	MALIGNANT NEOPLASM OF UNSPECIFIED KIDNEY, EXCEPT RENAL PELVIS	1890	MALIG NEOPL KIDNEY
C65	MALIGNANT NEOPLASM OF UNSPECIFIED RENAL PELVIS	1891	MALIG NEO RENAL PELVIS
C66	MALIGNANT NEOPLASM OF UNSPECIFIED URETER	1892	MALIGN NEOPL URETER
C680	MALIGNANT NEOPLASM OF URETHRA	1893	MALIGN NEOPL URETHRA
C681	MALIGNANT NEOPLASM OF PARAURETHRAL GLANDS	1894	MAL NEO PARAURETHRAL
C688	MALIGNANT NEOPLASM OF OVERLAPPING SITES OF URINARY ORGANS	1898	MAL NEO URINARY NEC
C689	MALIGNANT NEOPLASM OF URINARY ORGAN, UNSPECIFIED	1899	MAL NEO URINARY NOS
C69	Malignant neoplasm of eye and adnexa	190	MALIGNANT NEOPLASM EYE*
C6940	MALIGNANT NEOPLASM OF UNSPECIFIED CILIARY BODY	1900	MALIGN NEOPL EYEBALL
C6960	MALIGNANT NEOPLASM OF UNSPECIFIED ORBIT	1901	MALIGN NEOPL ORBIT
C6950	MALIGNANT NEOPLASM OF UNSPECIFIED LACRIMAL GLAND AND DUCT	1902	MAL NEO LACRIMAL GLAND
C6900	MALIGNANT NEOPLASM OF UNSPECIFIED CONJUNCTIVA	1903	MAL NEO CONJUNCTIVA
C6910	MALIGNANT NEOPLASM OF UNSPECIFIED CORNEA	1904	MALIGN NEOPL CORNEA

C6920	MALIGNANT NEOPLASM OF UNSPECIFIED RETINA	1905	MALIGN NEOPL RETINA
C6930	MALIGNANT NEOPLASM OF UNSPECIFIED CHOROID	1906	MALIGN NEOPL CHOROID
C6950	MALIGNANT NEOPLASM OF UNSPECIFIED LACRIMAL GLAND AND DUCT	1907	MAL NEO LACRIMAL DUCT
C6980	MALIGNANT NEOPLASM OF OVERLAPPING SITES OF UNSPECIFIED EYE AND ADNEXA	1908	MALIGN NEOPL EYE NEC
C699	MALIGNANT NEOPLASM OF UNSPECIFIED SITE OF UNSPECIFIED EYE	1909	MALIGN NEOPL EYE NOS
C71	Malignant neoplasm of brain	191	MALIGNANT NEOPLASM BRAIN*
C710	MALIGNANT NEOPLASM OF CEREBRUM, EXCEPT LOBES AND VENTRICLES	1910	MALIGN NEOPL CEREBRUM
C711	MALIGNANT NEOPLASM OF FRONTAL LOBE	1911	MALIG NEO FRONTAL LOBE
C712	MALIGNANT NEOPLASM OF TEMPORAL LOBE	1912	MAL NEO TEMPORAL LOBE
C713	MALIGNANT NEOPLASM OF PARIETAL LOBE	1913	MAL NEO PARIETAL LOBE
C714	MALIGNANT NEOPLASM OF OCCIPITAL LOBE	1914	MAL NEO OCCIPITAL LOBE
C715	MALIGNANT NEOPLASM OF CEREBRAL VENTRICLE	1915	MAL NEO CEREB VENTRICLE
C716	MALIGNANT NEOPLASM OF CEREBELLUM	1916	MAL NEO CEREBELLUM NOS
C717	MALIGNANT NEOPLASM OF BRAIN STEM	1917	MAL NEO BRAIN STEM
C718	MALIGNANT NEOPLASM OF OVERLAPPING SITES OF BRAIN	1918	MALIG NEO BRAIN NEC
C719	MALIGNANT NEOPLASM OF BRAIN, UNSPECIFIED	1919	MALIG NEO BRAIN NOS
C72	Malignant neoplasm of spinal cord, cranial nerves and other parts of central nervous system	192	MAL NEO NERVE NEC/NOS*
C7250	MALIGNANT NEOPLASM OF UNSPECIFIED CRANIAL NERVE	1920	MAL NEO CRANIAL NERVES
C700	MALIGNANT NEOPLASM OF CEREBRAL MENINGES	1921	MAL NEO CEREBRAL MENING
C720	MALIGNANT NEOPLASM OF SPINAL CORD	1922	MAL NEO SPINAL CORD
C701	MALIGNANT NEOPLASM OF SPINAL MENINGES	1923	MAL NEO SPINAL MENINGES
C728	MALIGNANT NEOPLASM OF CENTRAL NERVOUS SYSTEM, UNSPECIFIED	1928	MAL NEO NERVOUS SYST NEC
C729	MALIGNANT NEOPLASM OF CENTRAL NERVOUS SYSTEM, UNSPECIFIED	1929	MAL NEO NERVOUS SYST NOS
C73	MALIGNANT NEOPLASM OF THYROID GLAND	193	MALIGN NEOPL THYROID
C759	MALIGNANT NEOPLASM OF ENDOCRINE GLAND, UNSPECIFIED	194	MAL NEO OTHER ENDOCRINE*
C7490	MALIGNANT NEOPLASM OF UNSPECIFIED PART OF UNSPECIFIED ADRENAL GLAND	1940	MALIGN NEOPL ADRENAL
C750	MALIGNANT NEOPLASM OF PARATHYROID GLAND	1941	MALIG NEO PARATHYROID
C751	MALIGNANT NEOPLASM OF PITUITARY GLAND	1943	MALIG NEO PITUITARY
C753	MALIGNANT NEOPLASM OF PINEAL GLAND	1944	MALIGN NEO PINEAL GLAND
C754	MALIGNANT NEOPLASM OF CAROTID BODY	1945	MAL NEO CAROTID BODY
C755	MALIGNANT NEOPLASM OF AORTIC BODY AND OTHER PARAGANGLIA	1946	MAL NEO PARAGANGLIA NEC
C758	MALIGNANT NEOPLASM WITH PLURIGLANDULAR INVOLVEMENT, UNSPECIFIED	1948	MAL NEO ENDOCRINE NEC
C759	MALIGNANT NEOPLASM OF ENDOCRINE GLAND, UNSPECIFIED	1949	MAL NEO ENDOCRINE NOS
C76	Malignant neoplasm of other and ill-defined sites	195	MAL NEO OTH/ILL-DEF SITE*
C760	MALIGNANT NEOPLASM OF HEAD, FACE AND NECK	1950	MAL NEO HEAD/FACE/NECK
C761	MALIGNANT NEOPLASM OF THORAX	1951	MALIGN NEOPL THORAX
C762	MALIGNANT NEOPLASM OF ABDOMEN	1952	MALIG NEO ABDOMEN
C763	MALIGNANT NEOPLASM OF PELVIS	1953	MALIGN NEOPL PELVIS
C764	MALIGNANT NEOPLASM OF UPPER LIMB	1954	MALIGN NEOPL ARM
C765	MALIGNANT NEOPLASM OF LOWER LIMB	1955	MALIGN NEOPL LEG
C768	MALIGNANT NEOPLASM OF OTHER SPECIFIED ILL-DEFINED SITES	1958	MALIG NEO SITE NEC
C77	Secondary and unspecified malignant neoplasm of lymph nodes	196	MALIG NEO LYMPH NODES*
C770	SECONDARY AND UNSPECIFIED MALIGNANT NEOPLASM OF LYMPH NODES OF HEAD, FACE AND NECK	1960	MAL NEO LYMPH-HEAD/NECK

C771	SECONDARY AND UNSPECIFIED MALIGNANT NEOPLASM OF INTRATHORACIC LYMPH NODES	1961	MAL NEO LYMPH-INTRATHOR
C772	SECONDARY AND UNSPECIFIED MALIGNANT NEOPLASM OF INTRA-ABDOMINAL LYMPH NODES	1962	MAL NEO LYMPH INTRA-ABD
C773	SECONDARY AND UNSPECIFIED MALIGNANT NEOPLASM OF AXILLA AND UPPER LIMB LYMPH NODES	1963	MAL NEO LYMPH-AXILLA/ARM
C774	SECONDARY AND UNSPECIFIED MALIGNANT NEOPLASM OF INGUINAL AND LOWER LIMB LYMPH NODES	1965	MAL NEO LYMPH-INGUIN/LEG
C775	SECONDARY AND UNSPECIFIED MALIGNANT NEOPLASM OF INTRAPELVIC LYMPH NODES	1966	MAL NEO LYMPH-INTRAPELV
C778	SECONDARY AND UNSPECIFIED MALIGNANT NEOPLASM OF LYMPH NODES OF MULTIPLE REGIONS	1968	MAL NEO LYMPH NODE-MULT
C779	SECONDARY AND UNSPECIFIED MALIGNANT NEOPLASM OF LYMPH NODE, UNSPECIFIED	1969	MAL NEO LYMPH NODE NOS
C78	Secondary malignant neoplasm of respiratory and digestive organs	197	SECONDRY MAL NEO GI/RESP*
C7800	SECONDARY MALIGNANT NEOPLASM OF UNSPECIFIED LUNG	1970	SECONDARY MALIG NEO LUNG
C781	SECONDARY MALIGNANT NEOPLASM OF MEDIASTINUM	1971	SEC MAL NEO MEDIASTINUM
C782	SECONDARY MALIGNANT NEOPLASM OF PLEURA	1972	SECOND MALIG NEO PLEURA
C7839	SECONDARY MALIGNANT NEOPLASM OF OTHER RESPIRATORY ORGANS	1973	SEC MALIG NEO RESP NEC
C784	SECONDARY MALIGNANT NEOPLASM OF SMALL INTESTINE	1974	SEC MALIG NEO SM BOWEL
C785	SECONDARY MALIGNANT NEOPLASM OF LARGE INTESTINE AND RECTUM	1975	SEC MALIG NEO LG BOWEL
C786	SECONDARY MALIGNANT NEOPLASM OF RETROPERITONEUM AND PERITONEUM	1976	SEC MAL NEO PERITONEUM
C787	SECONDARY MALIGNANT NEOPLASM OF LIVER AND INTRAHEPATIC BILE DUCT	1977	SECOND MALIG NEO LIVER
C788	SECONDARY MALIGNANT NEOPLASM OF OTHER DIGESTIVE ORGANS	1978	SEC MAL NEO GI NEC
C79	Secondary malignant neoplasm of other and unspecified sites	198	SEC MALIG NEO OTH SITES*
C7900	SECONDARY MALIGNANT NEOPLASM OF UNSPECIFIED KIDNEY AND RENAL PELVIS	1980	SECOND MALIG NEO KIDNEY
C7919	SECONDARY MALIGNANT NEOPLASM OF OTHER URINARY ORGANS	1981	SEC MALIG NEO URIN NEC
C792	SECONDARY MALIGNANT NEOPLASM OF SKIN	1982	SECONDARY MALIG NEO SKIN
C7931	SECONDARY MALIGNANT NEOPLASM OF BRAIN	1983	SEC MAL NEO BRAIN/SPINE
C7949	SECONDARY MALIGNANT NEOPLASM OF OTHER PARTS OF NERVOUS SYSTEM	1984	SEC MALIG NEO NERVE NEC
C795	Secondary malignant neoplasm of bone and bone marrow	1985	SECONDARY MALIG NEO BONE
C7960	SECONDARY MALIGNANT NEOPLASM OF UNSPECIFIED OVARY	1986	SECOND MALIG NEO OVARY
C797	SECONDARY MALIGNANT NEOPLASM OF UNSPECIFIED ADRENAL GLAND	1987	SECOND MALIG NEO ADRENAL
C798	Secondary malignant neoplasm of other specified sites	1988	OTH SECONDARY MALIG NEO*
C7981	SECONDARY MALIGNANT NEOPLASM OF BREAST	19881	SECOND MALIG NEO BREAST
19882	SECONDARY MALIGNANT NEOPLASM OF GENITAL ORGANS	19882	SECOND MALIG NEO GENITAL
C7989	SECONDARY MALIGNANT NEOPLASM OF OTHER SPECIFIED SITES	19889	SECONDARY MALIG NEO NEC
C80	Malignant neoplasm without specification of site	199	MALIGNANT NEOPLASM NOS*
C800	DISSEMINATED MALIGNANT NEOPLASM, UNSPECIFIED	1990	MALIG NEO DISSEMINATED
C801	MALIGNANT (PRIMARY) NEOPLASM, UNSPECIFIED	1991	MALIGNANT NEOPLASM NOS
C8331	Non-follicular lymphoma	200	LYMPHOSARC/RETICULOSARC*
C830	Diffuse large B-cell lymphoma, unspecified site	2000	RETICULOSARCOMA*
C8330	DIFFUSE LARGE B-CELL LYMPHOMA, UNSPECIFIED SITE	20000	RETCLSRC UNSP XTRNDL ORG
C8331	DIFFUSE LARGE B-CELL LYMPHOMA, LYMPH NODES OF HEAD, FACE, AND NECK	20001	RETICULOSARCOMA HEAD
C8332	DIFFUSE LARGE B-CELL LYMPHOMA, INTRATHORACIC LYMPH NODES	20002	RETICULOSARCOMA THORAX
C8333	DIFFUSE LARGE B-CELL LYMPHOMA, INTRA-ABDOMINAL LYMPH NODES	20003	RETICULOSARCOMA ABDOM
C8334	DIFFUSE LARGE B-CELL LYMPHOMA, LYMPH NODES OF AXILLA AND UPPER LIMB	20004	RETICULOSARCOMA AXILLA
C8335	DIFFUSE LARGE B-CELL LYMPHOMA, LYMPH NODES OF INGUINAL REGION AND LOWER LIMB	20005	RETICULOSARCOMA INGUIN
C8336	DIFFUSE LARGE B-CELL LYMPHOMA, INTRAPELVIC LYMPH NODES	20006	RETICULOSARCOMA PELVIC
C8337	DIFFUSE LARGE B-CELL LYMPHOMA, SPLEEN	20007	RETICULOSARCOMA SPLEEN

C8338	DIFFUSE LARGE B-CELL LYMPHOMA, LYMPH NODES OF MULTIPLE SITES	20008	RETICULOSARCOMA MULT
C835	Lymphoblastic (diffuse) lymphoma	2001	LYMPHOSARCOMA*
C8350	LYMPHOBLASTIC (DIFFUSE) LYMPHOMA, UNSPECIFIED SITE	20010	LYMPHSRC UNSP XTRNDL ORG
C8351	LYMPHOBLASTIC (DIFFUSE) LYMPHOMA, LYMPH NODES OF HEAD, FACE, AND NECK	20011	LYMPHOSARCOMA HEAD
C8352	LYMPHOBLASTIC (DIFFUSE) LYMPHOMA, INTRATHORACIC LYMPH NODES	20012	LYMPHOSARCOMA THORAX
C8353	LYMPHOBLASTIC (DIFFUSE) LYMPHOMA, INTRA-ABDOMINAL LYMPH NODES	20013	LYMPHOSARCOMA ABDOM
C8354	LYMPHOBLASTIC (DIFFUSE) LYMPHOMA, LYMPH NODES OF AXILLA AND UPPER LIMB	20014	LYMPHOSARCOMA AXILLA
C8355	LYMPHOBLASTIC (DIFFUSE) LYMPHOMA, LYMPH NODES OF INGUINAL REGION AND LOWER LIMB	20015	LYMPHOSARCOMA INGUIN
C8356	LYMPHOBLASTIC (DIFFUSE) LYMPHOMA, INTRAPELVIC LYMPH NODES	20016	LYMPHOSARCOMA PELVIC
C8357	LYMPHOBLASTIC (DIFFUSE) LYMPHOMA, SPLEEN	20017	LYMPHOSARCOMA SPLEEN
C8358	LYMPHOBLASTIC (DIFFUSE) LYMPHOMA, LYMPH NODES OF MULTIPLE SITES	20018	LYMPHOSARCOMA MULT
C837	Burkitt lymphoma	2002	BURKITT'S TUMOR/LYMPHOMA*
C8370	BURKITT LYMPHOMA, UNSPECIFIED SITE	20020	BRKT TMR UNSP XTRNDL ORG
C8371	BURKITT LYMPHOMA, LYMPH NODES OF HEAD, FACE, AND NECK	20021	BURKITT'S TUMOR HEAD
C8372	BURKITT LYMPHOMA, INTRATHORACIC LYMPH NODES	20022	BURKITT'S TUMOR THORAX
C8373	BURKITT LYMPHOMA, INTRA-ABDOMINAL LYMPH NODES	20023	BURKITT'S TUMOR ABDOM
C8374	BURKITT LYMPHOMA, LYMPH NODES OF AXILLA AND UPPER LIMB	20024	BURKITT'S TUMOR AXILLA
C8375	BURKITT LYMPHOMA, LYMPH NODES OF INGUINAL REGION AND LOWER LIMB	20025	BURKITT'S TUMOR INGUIN
C8376	BURKITT LYMPHOMA, INTRAPELVIC LYMPH NODES	20026	BURKITT'S TUMOR PELVIC
C8377	BURKITT LYMPHOMA, SPLEEN	20027	BURKITT'S TUMOR SPLEEN
C8378	BURKITT LYMPHOMA, LYMPH NODES OF MULTIPLE SITES	20028	BURKITT'S TUMOR MULT
C8389	OTHER NON-FOLLICULAR LYMPHOMA, EXTRANODAL AND SOLID ORGAN SITES	20030	MARGINAL ZONE LYMPHOMA, UNSPECIFIED SITE, EXTRANODAL AND SOLID ORGAN SITES
C8381	OTHER NON-FOLLICULAR LYMPHOMA, LYMPH NODES OF HEAD, FACE, AND NECK	20031	MARGINAL ZONE LYMPHOMA, LYMPH NODES OF HEAD, FACE AND NECK
C8382	OTHER NON-FOLLICULAR LYMPHOMA, INTRATHORACIC LYMPH NODES	20032	MARGINAL ZONE LYMPHOMA, INTRATHORACIC LYMPH NODES
C8383	OTHER NON-FOLLICULAR LYMPHOMA, INTRA-ABDOMINAL LYMPH NODES	20033	MARGINAL ZONE LYMPHOMA, INTRAABDOMINAL LYMPH NODES
C8384	OTHER NON-FOLLICULAR LYMPHOMA, LYMPH NODES OF AXILLA AND UPPER LIMB	20034	MARGINAL ZONE LYMPHOMA, LYMPH NODES OF AXILLA AND UPPER LIMB
C8385	OTHER NON-FOLLICULAR LYMPHOMA, LYMPH NODES OF INGUINAL REGION AND LOWER LIMB	20035	MARGINAL ZONE LYMPHOMA, LYMPH NODES OF INGUINAL REGION AND LOWER LIMB
C8386	OTHER NON-FOLLICULAR LYMPHOMA, INTRAPELVIC LYMPH NODES	20036	MARGINAL ZONE LYMPHOMA, INTRAPELVIC LYMPH NODES
C8387	OTHER NON-FOLLICULAR LYMPHOMA, SPLEEN	20037	MARGINAL ZONE LYMPHOMA, SPLEEN
C8388	OTHER NON-FOLLICULAR LYMPHOMA, LYMPH NODES OF MULTIPLE SITES	20038	MARGINAL ZONE LYMPHOMA, LYMPH NODES OF MULTIPLE SITES
C8319	MANTLE CELL LYMPHOMA, EXTRANODAL AND SOLID ORGAN SITES	20040	MANTLE CELL LYMPHOMA, UNSPECIFIED SITE, EXTRANODAL AND SOLID ORGAN SITES
C8311	MANTLE CELL LYMPHOMA, LYMPH NODES OF HEAD, FACE, AND NECK	20041	MANTLE CELL LYMPHOMA, LYMPH NODES OF HEAD, FACE AND NECK
C8312	MANTLE CELL LYMPHOMA, INTRATHORACIC LYMPH NODES	20042	MANTLE CELL LYMPHOMA, INTRATHORACIC LYMPH NODES
C8313	MANTLE CELL LYMPHOMA, INTRA-ABDOMINAL LYMPH NODES	20043	MANTLE CELL LYMPHOMA, INTRA-ABDOMINAL LYMPH NODES
C8314	MANTLE CELL LYMPHOMA, LYMPH NODES OF AXILLA AND UPPER LIMB	20044	MANTLE CELL LYMPHOMA, LYMPH NODES OF AXILLA AND UPPER LIMB
C8315	MANTLE CELL LYMPHOMA, LYMPH NODES OF INGUINAL REGION AND LOWER LIMB	20045	MANTLE CELL LYMPHOMA, LYMPH NODES OF INGUINAL REGION AND LOWER LIMB

C8316	MANTLE CELL LYMPHOMA, INTRAPELVIC LYMPH NODES	20046	MANTLE CELL LYMPHOMA, INTRAPELVIC LYMPH NODES
C8317	MANTLE CELL LYMPHOMA, SPLEEN	20047	MANTLE CELL LYMPHOMA, SPLEEN
C8318	MANTLE CELL LYMPHOMA, LYMPH NODES OF MULTIPLE SITE	20048	MANTLE CELL LYMPHOMA, LYMPH NODES MULTIPLE SITES
C8339	DIFFUSE LARGE B-CELL LYMPHOMA, EXTRANODAL AND SOLID ORGAN SITES	20050	PRIMARY CENTRAL NERVOUS SYSTEM LYMPHOMA, UNSPECIFIED SITE, EXTRANODAL AND SOLID ORGAN SITES
C8331	DIFFUSE LARGE B-CELL LYMPHOMA, LYMPH NODES OF HEAD, FACE, AND NECK	20051	PRIMARY CENTRAL NERVOUS SYSTEM LYMPHOMA, LYMPH NODES OF HEAD, FACE, AND NECK
C8332	DIFFUSE LARGE B-CELL LYMPHOMA, INTRATHORACIC LYMPH NODES	20052	PRIMARY CENTRAL NERVOUS SYSTEM LYMPHOMA, INTRATHORACIC LYMPH NODES
C8333	DIFFUSE LARGE B-CELL LYMPHOMA, INTRA-ABDOMINAL LYMPH NODES	20053	PRIMARY CENTRAL NERVOUS SYSTEM LYMPHOMA, INTRA-ABDOMINAL LYMPH NODES
C8334	DIFFUSE LARGE B-CELL LYMPHOMA, LYMPH NODES OF AXILLA AND UPPER LIMB	20054	PRIMARY CENTRAL NERVOUS SYSTEM LYMPHOMA, LYMPH NODES OF AXILLA AND UPPER LIMB
C8335	DIFFUSE LARGE B-CELL LYMPHOMA, LYMPH NODES OF INGUINAL REGION AND LOWER LIMB	20055	PRIMARY CENTRAL NERVOUS SYSTEM LYMPHOMA, LYMPH NODES OF INGUINAL REGION AND LOWER LIMB
C8336	DIFFUSE LARGE B-CELL LYMPHOMA, INTRAPELVIC LYMPH NODES	20056	PRIMARY CENTRAL NERVOUS SYSTEM LYMPHOMA, INTRAPELVIC LYMPH NODES
C8337	DIFFUSE LARGE B-CELL LYMPHOMA, SPLEEN	20057	PRIMARY CENTRAL NERVOUS SYSTEM LYMPHOMA, SPLEEN
C8338	DIFFUSE LARGE B-CELL LYMPHOMA, LYMPH NODES OF MULTIPLE SITES	20058	PRIMARY CENTRAL NERVOUS SYSTEM LYMPHOMA, LYMPH NODES OF MULTIPLE SITES
C8479	ANAPLASTIC LARGE CELL LYMPHOMA, ALK-NEGATIVE, EXTRANODAL AND SOLID ORGAN SITES	20060	ANAPLASTIC LARGE CELL LYMPHOMA, UNSPECIFIED SITE, EXTRANODAL AND SOLID ORGAN SITES
C8471	ANAPLASTIC LARGE CELL LYMPHOMA, ALK-NEGATIVE, LYMPH NODES OF HEAD, FACE, AND NECK	20061	ANAPLASTIC LARGE CELL LYMPHOMA, LYMPH NODES OF HEAD, FACE, AND NECK
C8472	ANAPLASTIC LARGE CELL LYMPHOMA, ALK-NEGATIVE, INTRATHORACIC LYMPH NODES	20062	ANAPLASTIC LARGE CELL LYMPHOMA, INTRATHORACIC LYMPH NODES
C8473	ANAPLASTIC LARGE CELL LYMPHOMA, ALK-NEGATIVE, INTRA-ABDOMINAL LYMPH NODES	20063	ANAPLASTIC LARGE CELL LYMPHOMA, INTRA-ABDOMINAL LYMPH NODES
C8474	ANAPLASTIC LARGE CELL LYMPHOMA, ALK-NEGATIVE, LYMPH NODES OF AXILLA AND UPPER LIMB	20064	ANAPLASTIC LARGE CELL LYMPHOMA, LYMPH NODES OF AXILLA AND UPPER LIMB
C8475	ANAPLASTIC LARGE CELL LYMPHOMA, ALK-NEGATIVE, LYMPH NODES OF INGUINAL REGION AND LOWER LIMB	20065	ANAPLASTIC LARGE CELL LYMPHOMA, LYMPH NODES OF INGUINAL REGION AND LOWER LIMB
C8476	ANAPLASTIC LARGE CELL LYMPHOMA, ALK-NEGATIVE, INTRAPELVIC LYMPH NODES	20066	ANAPLASTIC LARGE CELL LYMPHOMA, INTRAPELVIC LYMPH NODES
C8477	ANAPLASTIC LARGE CELL LYMPHOMA, ALK-NEGATIVE, SPLEEN	20067	ANAPLASTIC LARGE CELL LYMPHOMA, SPLEEN
C8478	ANAPLASTIC LARGE CELL LYMPHOMA, ALK-NEGATIVE, LYMPH NODES OF MULTIPLE SITES	20068	ANAPLASTIC LARGE CELL LYMPHOMA, LYMPH NODES OF MULTIPLE SITES
C8339	DIFFUSE LARGE B-CELL LYMPHOMA, EXTRANODAL AND SOLID ORGAN SITES	20070	LARGE CELL LYMPHOMA, UNSPECIFIED SITE, EXTRANODAL AND SOLID ORGAN SITES
C8331	DIFFUSE LARGE B-CELL LYMPHOMA, LYMPH NODES OF HEAD, FACE, AND NECK	20071	LARGE CELL LYMPHOMA, LYMPH NODES OF HEAD, FACE, AND NECK
C8332	DIFFUSE LARGE B-CELL LYMPHOMA, INTRATHORACIC LYMPH NODES	20072	LARGE CELL LYMPHOMA, INTRATHORACIC LYMPH NODES
C8333	DIFFUSE LARGE B-CELL LYMPHOMA, INTRA-ABDOMINAL LYMPH NODES	20073	LARGE CELL LYMPHOMA, INTRA-ABDOMINAL LYMPH NODES
C8334	DIFFUSE LARGE B-CELL LYMPHOMA, LYMPH NODES OF AXILLA AND UPPER LIMB	20074	LARGE CELL LYMPHOMA, LYMPH NODES OF AXILLA AND UPPER LIMB

C8335	DIFFUSE LARGE B-CELL LYMPHOMA, LYMPH NODES OF INGUINAL REGION AND LOWER LIMB	20075	LARGE CELL LYMPHOMA, LYMPH NODES OF INGUINAL REGION AND LOWER LIMB
C8336	DIFFUSE LARGE B-CELL LYMPHOMA, INTRAPELVIC LYMPH NODES	20076	LARGE CELL LYMPHOMA, INTRAPELVIC LYMPH NODES
C8337	DIFFUSE LARGE B-CELL LYMPHOMA, SPLEEN	20077	LARGE CELL LYMPHOMA, SPLEEN
C8338	DIFFUSE LARGE B-CELL LYMPHOMA, LYMPH NODES OF MULTIPLE SITES	20078	LARGE CELL LYMPHOMA, LYMPH NODES OF MULTIPLE SITES
C838	Other non-follicular lymphoma	2008	MIXED LYMPHOSARCOMA*
C8380	OTHER NON-FOLLICULAR LYMPHOMA, UNSPECIFIED SITE	20080	OTH VARN UNSP XTRNDL ORG
C8381	OTHER NON-FOLLICULAR LYMPHOMA, LYMPH NODES OF HEAD, FACE, AND NECK	20081	MIXED LYMPHOSARC HEAD
C8382	OTHER NON-FOLLICULAR LYMPHOMA, INTRATHORACIC LYMPH NODES	20082	MIXED LYMPHOSARC THORAX
C8383	OTHER NON-FOLLICULAR LYMPHOMA, INTRA-ABDOMINAL LYMPH NODES	20083	MIXED LYMPHOSARC ABDOM
C8384	OTHER NON-FOLLICULAR LYMPHOMA, LYMPH NODES OF AXILLA AND UPPER LIMB	20084	MIXED LYMPHOSARC AXILLA
C8385	OTHER NON-FOLLICULAR LYMPHOMA, LYMPH NODES OF INGUINAL REGION AND LOWER LIMB	20085	MIXED LYMPHOSARC INGUIN
C8386	OTHER NON-FOLLICULAR LYMPHOMA, INTRAPELVIC LYMPH NODES	20086	MIXED LYMPHOSARC PELVIC
C8387	OTHER NON-FOLLICULAR LYMPHOMA, SPLEEN	20087	MIXED LYMPHOSARC SPLEEN
C8388	OTHER NON-FOLLICULAR LYMPHOMA, LYMPH NODES OF MULTIPLE SITES	20088	MIXED LYMPHOSARC MULT
C81	Hodgkin lymphoma	201	HODGKIN'S DISEASE*
C8170	OTHER CLASSICAL HODGKIN LYMPHOMA, UNSPECIFIED SITE	2010	HODGKIN'S PARAGRANULOMA*
C8179	OTHER CLASSICAL HODGKIN LYMPHOMA, EXTRANODAL AND SOLID ORGAN SITES	20100	HDGK PRG UNSP XTRNDL ORG
C8171	OTHER CLASSICAL HODGKIN LYMPHOMA, LYMPH NODES OF HEAD, FACE, AND NECK	20101	HODGKINS PARAGRAN HEAD
C8172	OTHER CLASSICAL HODGKIN LYMPHOMA, INTRATHORACIC LYMPH NODES	20102	HODGKINS PARAGRAN THORAX
C8173	OTHER CLASSICAL HODGKIN LYMPHOMA, INTRA-ABDOMINAL LYMPH NODES	20103	HODGKINS PARAGRAN ABDOM
C8174	OTHER CLASSICAL HODGKIN LYMPHOMA, LYMPH NODES OF AXILLA AND UPPER LIMB	20104	HODGKINS PARAGRAN AXILLA
C8175	OTHER CLASSICAL HODGKIN LYMPHOMA, LYMPH NODES OF INGUINAL REGION AND LOWER LIMB	20105	HODGKINS PARAGRAN INGUIN
C8176	OTHER CLASSICAL HODGKIN LYMPHOMA, INTRAPELVIC LYMPH NODES	20106	HODGKINS PARAGRAN PELVIC
C8177	OTHER CLASSICAL HODGKIN LYMPHOMA, SPLEEN	20107	HODGKINS PARAGRAN SPLEEN
C8178	OTHER CLASSICAL HODGKIN LYMPHOMA, LYMPH NODES OF MULTIPLE SITES	20108	HODGKINS PARAGRAN MULT
C817	Other Hodgkin lymphoma	2011	HODGKIN'S GRANULOMA*
C8179	OTHER CLASSICAL HODGKIN LYMPHOMA, EXTRANODAL AND SOLID ORGAN SITES	20110	HDGK GRN UNSP XTRNDL ORG
C8171	OTHER CLASSICAL HODGKIN LYMPHOMA, LYMPH NODES OF HEAD, FACE, AND NECK	20111	HODGKINS GRANULOM HEAD
C8172	OTHER CLASSICAL HODGKIN LYMPHOMA, INTRATHORACIC LYMPH NODES	20112	HODGKINS GRANULOM THORAX
C8173	OTHER CLASSICAL HODGKIN LYMPHOMA, INTRA-ABDOMINAL LYMPH NODES	20113	HODGKINS GRANULOM ABDOM
C8174	OTHER CLASSICAL HODGKIN LYMPHOMA, LYMPH NODES OF AXILLA AND UPPER LIMB	20114	HODGKINS GRANULOM AXILLA
C8175	OTHER CLASSICAL HODGKIN LYMPHOMA, LYMPH NODES OF INGUINAL REGION AND LOWER LIMB	20115	HODGKINS GRANULOM INGUIN
C8176	OTHER CLASSICAL HODGKIN LYMPHOMA, INTRAPELVIC LYMPH NODES	20116	HODGKINS GRANULOM PELVIC
C8177	OTHER CLASSICAL HODGKIN LYMPHOMA, SPLEEN	20117	HODGKINS GRANULOM SPLEEN
C8178	OTHER CLASSICAL HODGKIN LYMPHOMA, LYMPH NODES OF MULTIPLE SITES	20118	HODGKINS GRANULOM MULT
C817	Other Hodgkin lymphoma	2012	HODGKIN'S SARCOMA*
C8179	OTHER CLASSICAL HODGKIN LYMPHOMA, EXTRANODAL AND SOLID ORGAN SITES	20120	HDGK SRC UNSP XTRNDL ORG
C8171	OTHER CLASSICAL HODGKIN LYMPHOMA, LYMPH NODES OF HEAD, FACE, AND NECK	20121	HODGKINS SARCOMA HEAD
C8172	OTHER CLASSICAL HODGKIN LYMPHOMA, INTRATHORACIC LYMPH NODES	20122	HODGKINS SARCOMA THORAX
C8173	OTHER CLASSICAL HODGKIN LYMPHOMA, INTRA-ABDOMINAL LYMPH NODES	20123	HODGKINS SARCOMA ABDOM
C8174	OTHER CLASSICAL HODGKIN LYMPHOMA, LYMPH NODES OF AXILLA AND UPPER LIMB	20124	HODGKINS SARCOMA AXILLA
C8175	OTHER CLASSICAL HODGKIN LYMPHOMA, LYMPH NODES OF INGUINAL REGION AND LOWER LIMB	20125	HODGKINS SARCOMA INGUIN

C8176	OTHER CLASSICAL HODGKIN LYMPHOMA, INTRAPELVIC LYMPH NODES	20126	HODGKINS SARCOMA PELVIC
C8177	OTHER CLASSICAL HODGKIN LYMPHOMA, SPLEEN	20127	HODGKINS SARCOMA SPLEEN
C8178	OTHER CLASSICAL HODGKIN LYMPHOMA, LYMPH NODES OF MULTIPLE SITES	20128	HODGKINS SARCOMA MULT
C810	Nodular lymphocyte predominant Hodgkin lymphoma	2014	HODGKINS LYMPH-HISTIOCYT*
C8100	NODULAR LYMPHOCYTE PREDOMINANT HODGKIN LYMPHOMA, UNSPECIFIED SITE	20140	LYM-HST UNSP XTRNDL ORGN
C8101	NODULAR LYMPHOCYTE PREDOMINANT HODGKIN LYMPHOMA, LYMPH NODES OF HEAD, FACE, AND NECK	20141	HODG LYMPH-HISTIO HEAD
C8102	NODULAR LYMPHOCYTE PREDOMINANT HODGKIN LYMPHOMA, INTRATHORACIC LYMPH NODES	20142	HODG LYMPH-HISTIO THORAX
C8103	NODULAR LYMPHOCYTE PREDOMINANT HODGKIN LYMPHOMA, INTRA-ABDOMINAL LYMPH NODES	20143	HODG LYMPH-HISTIO ABDOM
C8104	NODULAR LYMPHOCYTE PREDOMINANT HODGKIN LYMPHOMA, LYMPH NODES OF AXILLA AND UPPER LIMB	20144	HODG LYMPH-HISTIO AXILLA
C8105	NODULAR LYMPHOCYTE PREDOMINANT HODGKIN LYMPHOMA, LYMPH NODES OF INGUINAL REGION AND LOWER LIMB	20145	HODG LYMPH-HISTIO INGUIN
C8106	NODULAR LYMPHOCYTE PREDOMINANT HODGKIN LYMPHOMA, INTRAPELVIC LYMPH NODES	20146	HODG LYMPH-HISTIO PELVIC
C8107	NODULAR LYMPHOCYTE PREDOMINANT HODGKIN LYMPHOMA, SPLEEN	20147	HODG LYMPH-HISTIO SPLEEN
C8108	NODULAR LYMPHOCYTE PREDOMINANT HODGKIN LYMPHOMA, LYMPH NODES OF MULTIPLE SITES	20148	HODG LYMPH-HISTIO MULT
C811	Nodular sclerosis Hodgkin lymphoma	2015	HODGKINS NODULAR SCLEROS*
C8110	NODULAR SCLEROSIS CLASSICAL HODGKIN LYMPHOMA, UNSPECIFIED SITE	20150	NDR SCLR UNSP XTRNDL ORG
C8111	NODULAR SCLEROSIS CLASSICAL HODGKIN LYMPHOMA, LYMPH NODES OF HEAD, FACE, AND NECK	20151	HODG NODUL SCLERO HEAD
C8112	NODULAR SCLEROSIS CLASSICAL HODGKIN LYMPHOMA, INTRATHORACIC LYMPH NODES	20152	HODG NODUL SCLERO THORAX
C8113	NODULAR SCLEROSIS CLASSICAL HODGKIN LYMPHOMA, INTRA-ABDOMINAL LYMPH NODES	20153	HODG NODUL SCLERO ABDOM
C8114	NODULAR SCLEROSIS CLASSICAL HODGKIN LYMPHOMA, LYMPH NODES OF AXILLA AND UPPER LIMB	20154	HODG NODUL SCLERO AXILLA
C8115	NODULAR SCLEROSIS CLASSICAL HODGKIN LYMPHOMA, LYMPH NODES OF INGUINAL REGION AND LOWER LIMB	20155	HODG NODUL SCLERO INGUIN
C8116	NODULAR SCLEROSIS CLASSICAL HODGKIN LYMPHOMA, INTRAPELVIC LYMPH NODES	20156	HODG NODUL SCLERO PELVIC
C8117	NODULAR SCLEROSIS CLASSICAL HODGKIN LYMPHOMA, SPLEEN	20157	HODG NODUL SCLERO SPLEEN
C8118	NODULAR SCLEROSIS CLASSICAL HODGKIN LYMPHOMA, LYMPH NODES OF MULTIPLE SITES	20158	HODG NODUL SCLERO MULT
C812	Mixed cellularity Hodgkin lymphoma	2016	HODGKINS MIX CELLULARITY*
C8120	MIXED CELLULARITY CLASSICAL HODGKIN LYMPHOMA, UNSPECIFIED SITE	20160	MXD CELR UNSP XTRNDL ORG
C8121	MIXED CELLULARITY CLASSICAL HODGKIN LYMPHOMA, LYMPH NODES OF HEAD, FACE, AND NECK	20161	HODGKINS MIX CELL HEAD
C8122	MIXED CELLULARITY CLASSICAL HODGKIN LYMPHOMA, INTRATHORACIC LYMPH NODES	20162	HODGKINS MIX CELL THORAX
C8123	MIXED CELLULARITY CLASSICAL HODGKIN LYMPHOMA, INTRA-ABDOMINAL LYMPH NODES	20163	HODGKINS MIX CELL ABDOM
C8124	MIXED CELLULARITY CLASSICAL HODGKIN LYMPHOMA, LYMPH NODES OF AXILLA AND UPPER LIMB	20164	HODGKINS MIX CELL AXILLA
C8125	MIXED CELLULARITY CLASSICAL HODGKIN LYMPHOMA, LYMPH NODES OF INGUINAL REGION AND LOWER LIMB	20165	HODGKINS MIX CELL INGUIN
C8126	MIXED CELLULARITY CLASSICAL HODGKIN LYMPHOMA, INTRAPELVIC LYMPH NODES	20166	HODGKINS MIX CELL PELVIC
C8127	MIXED CELLULARITY CLASSICAL HODGKIN LYMPHOMA, SPLEEN	20167	HODGKINS MIX CELL SPLEEN
C8128	MIXED CELLULARITY CLASSICAL HODGKIN LYMPHOMA, LYMPH NODES OF MULTIPLE SITES	20168	HODGKINS MIX CELL MULT
C813	Lymphocyte depleted Hodgkin lymphoma	2017	HODG LYMPHOCYTIC DEPLET*
C8130	LYMPHOCYTE DEPLETED CLASSICAL HODGKIN LYMPHOMA, UNSPECIFIED SITE	20170	LYM DPLT UNSP XTRNDL ORG
C8131	LYMPHOCYTE DEPLETED CLASSICAL HODGKIN LYMPHOMA, LYMPH NODES OF HEAD, FACE, AND NECK	20171	HODG LYMPH DEPLET HEAD
C8132	LYMPHOCYTE DEPLETED CLASSICAL HODGKIN LYMPHOMA, INTRATHORACIC LYMPH NODES	20172	HODG LYMPH DEPLET THORAX
C8133	LYMPHOCYTE DEPLETED CLASSICAL HODGKIN LYMPHOMA, INTRA-ABDOMINAL LYMPH NODES	20173	HODG LYMPH DEPLET ABDOM
C8134	LYMPHOCYTE DEPLETED CLASSICAL HODGKIN LYMPHOMA, LYMPH NODES OF AXILLA AND UPPER LIMB	20174	HODG LYMPH DEPLET AXILLA

C8135	LYMPHOCYTE DEPLETED CLASSICAL HODGKIN LYMPHOMA, LYMPH NODES OF INGUINAL REGION AND LOWER LIMB	20175	HODG LYMPH DEPLET INGUIN
C8136	LYMPHOCYTE DEPLETED CLASSICAL HODGKIN LYMPHOMA, INTRAPELVIC LYMPH NODES	20176	HODG LYMPH DEPLET PELVIC
C8137	LYMPHOCYTE DEPLETED CLASSICAL HODGKIN LYMPHOMA, SPLEEN	20177	HODG LYMPH DEPLET SPLEEN
C8138	LYMPHOCYTE DEPLETED CLASSICAL HODGKIN LYMPHOMA, LYMPH NODES OF MULTIPLE SITES	20178	HODG LYMPH DEPLET MULT
C819	Hodgkin lymphoma, unspecified	2019	HODGKINS DISEASE NOS*
C8190	HODGKIN LYMPHOMA, UNSPECIFIED, UNSPECIFIED SITE	20190	HDGK DIS UNSP XTRNDL ORG
C8191	HODGKIN LYMPHOMA, UNSPECIFIED, LYMPH NODES OF HEAD, FACE, AND NECK	20191	HODGKINS DIS NOS HEAD
C8192	HODGKIN LYMPHOMA, UNSPECIFIED, INTRATHORACIC LYMPH NODES	20192	HODGKINS DIS NOS THORAX
C8193	HODGKIN LYMPHOMA, UNSPECIFIED, INTRA-ABDOMINAL LYMPH NODES	20193	HODGKINS DIS NOS ABDOM
C8194	HODGKIN LYMPHOMA, UNSPECIFIED, LYMPH NODES OF AXILLA AND UPPER LIMB	20194	HODGKINS DIS NOS AXILLA
C8195	HODGKIN LYMPHOMA, UNSPECIFIED, LYMPH NODES OF INGUINAL REGION AND LOWER LIMB	20195	HODGKINS DIS NOS INGUIN
C8196	HODGKIN LYMPHOMA, UNSPECIFIED, INTRAPELVIC LYMPH NODES	20196	HODGKINS DIS NOS PELVIC
C8197	HODGKIN LYMPHOMA, UNSPECIFIED, SPLEEN	20197	HODGKINS DIS NOS SPLEEN
C8198	HODGKIN LYMPHOMA, UNSPECIFIED, LYMPH NODES OF MULTIPLE SITES	20198	HODGKINS DIS NOS MULT
C8290	FOLLICULAR LYMPHOMA, UNSPECIFIED, UNSPECIFIED SITE	20200	NDLR LYM UNSP XTRNDL ORG
C8291	FOLLICULAR LYMPHOMA, UNSPECIFIED, LYMPH NODES OF HEAD, FACE, AND NECK	20201	NODULAR LYMPHOMA HEAD
C8292	FOLLICULAR LYMPHOMA, UNSPECIFIED, INTRATHORACIC LYMPH NODES	20202	NODULAR LYMPHOMA THORAX
C8293	FOLLICULAR LYMPHOMA, UNSPECIFIED, INTRA-ABDOMINAL LYMPH NODES	20203	NODULAR LYMPHOMA ABDOM
C8294	FOLLICULAR LYMPHOMA, UNSPECIFIED, LYMPH NODES OF AXILLA AND UPPER LIMB	20204	NODULAR LYMPHOMA AXILLA
C8295	FOLLICULAR LYMPHOMA, UNSPECIFIED, LYMPH NODES OF INGUINAL REGION AND LOWER LIMB	20205	NODULAR LYMPHOMA INGUIN
C8296	FOLLICULAR LYMPHOMA, UNSPECIFIED, INTRAPELVIC LYMPH NODES	20206	NODULAR LYMPHOMA PELVIC
C8297	FOLLICULAR LYMPHOMA, UNSPECIFIED, SPLEEN	20207	NODULAR LYMPHOMA SPLEEN
C8298	FOLLICULAR LYMPHOMA, UNSPECIFIED, LYMPH NODES OF MULTIPLE SITES	20208	NODULAR LYMPHOMA MULT
C840	MYCOSIS FUNGOIDES	2021	MYCOSIS FUNGOIDES*
C8409	MYCOSIS FUNGOIDES, EXTRANODAL AND SOLID ORGAN SITES	20210	MYCS FNG UNSP XTRNDL ORG
C8401	MYCOSIS FUNGOIDES, LYMPH NODES OF HEAD, FACE, AND NECK	20211	MYCOSIS FUNGOIDES HEAD
C8401	MYCOSIS FUNGOIDES, INTRATHORACIC LYMPH NODES	20212	MYCOSIS FUNGOIDES THORAX
C8403	MYCOSIS FUNGOIDES, INTRA-ABDOMINAL LYMPH NODES	20213	MYCOSIS FUNGOIDES ABDOM
C8404	MYCOSIS FUNGOIDES, LYMPH NODES OF AXILLA AND UPPER LIMB	20214	MYCOSIS FUNGOIDES AXILLA
C8405	MYCOSIS FUNGOIDES, LYMPH NODES OF INGUINAL REGION AND LOWER LIMB	20215	MYCOSIS FUNGOIDES INGUIN
C8406	MYCOSIS FUNGOIDES, INTRAPELVIC LYMPH NODES	20216	MYCOSIS FUNGOIDES PELVIC
C8407	MYCOSIS FUNGOIDES, SPLEEN	20217	MYCOSIS FUNGOIDES SPLEEN
C8408	MYCOSIS FUNGOIDES, LYMPH NODES OF MULTIPLE SITES	20218	MYCOSIS FUNGOIDES MULT
C841	Sézary disease	2022	SEZARY'S DISEASE*
C8419	SEZARY DISEASE, EXTRANODAL AND SOLID ORGAN SITES	20220	SZRY DIS UNSP XTRNDL ORG
C8411	SEZARY DISEASE, LYMPH NODES OF HEAD, FACE, AND NECK	20221	SEZARY'S DISEASE HEAD
C8412	SEZARY DISEASE, INTRATHORACIC LYMPH NODES	20222	SEZARY'S DISEASE THORAX
C8413	SEZARY DISEASE, INTRA-ABDOMINAL LYMPH NODES	20223	SEZARY'S DISEASE ABDOM
C8414	SEZARY DISEASE, LYMPH NODES OF AXILLA AND UPPER LIMB	20224	SEZARY'S DISEASE AXILLA
C8415	SEZARY DISEASE, LYMPH NODES OF INGUINAL REGION AND LOWER LIMB	20225	SEZARY'S DISEASE INGUIN
C8416	SEZARY DISEASE, INTRAPELVIC LYMPH NODES	20226	SEZARY'S DISEASE PELVIC
C8417	SEZARY DISEASE, SPLEEN	20227	SEZARY'S DISEASE SPLEEN
C8418	SEZARY DISEASE, LYMPH NODES OF MULTIPLE SITES	20228	SEZARY'S DISEASE MULT

C96	Other and unspecified malignant neoplasms of lymphoid, hematopoietic and related tissue	2023	MALIGNANT HISTIOCYTOSIS*
C96A	HISTIOCYTIC SARCOMA	20230	MLG HIST UNSP XTRNDL ORG
C96A	HISTIOCYTIC SARCOMA	20231	MAL HISTIOCYTOSIS HEAD
C96A	HISTIOCYTIC SARCOMA	20232	MAL HISTIOCYTOSIS THORAX
C96A	HISTIOCYTIC SARCOMA	20233	MAL HISTIOCYTOSIS ABDOM
C96A	HISTIOCYTIC SARCOMA	20234	MAL HISTIOCYTOSIS AXILLA
C96A	HISTIOCYTIC SARCOMA	20235	MAL HISTIOCYTOSIS INGUIN
C96A	HISTIOCYTIC SARCOMA	20236	MAL HISTIOCYTOSIS PELVIC
C96A	HISTIOCYTIC SARCOMA	20237	MAL HISTIOCYTOSIS SPLEEN
C96A	HISTIOCYTIC SARCOMA	20238	MAL HISTIOCYTOSIS MULT
C914	Hairy cell leukemia	2024	LEUKEM RETICULOENDOTHEL*
C9140	Hairy cell leukemia not having achieved remission	20240	LK RTCTL UNSP XTRNDL ORG
C9140	Hairy cell leukemia not having achieved remission	20241	HAIRY-CELL LEUKEM HEAD
C9140	Hairy cell leukemia not having achieved remission	20242	HAIRY-CELL LEUKEM THORAX
C9140	Hairy cell leukemia not having achieved remission	20243	HAIRY-CELL LEUKEM ABDOM
C9140	Hairy cell leukemia not having achieved remission	20244	HAIRY-CELL LEUKEM AXILLA
C9140	Hairy cell leukemia not having achieved remission	20245	HAIRY-CELL LEUKEM INGUIN
C9140	Hairy cell leukemia not having achieved remission	20246	HAIRY-CELL LEUKEM PELVIC
C9140	Hairy cell leukemia not having achieved remission	20247	HAIRY-CELL LEUKEM SPLEEN
C9140	Hairy cell leukemia not having achieved remission	20248	HAIRY-CELL LEUKEM MULT
C960	MULTIFOCAL AND MULTISYSTEMIC (DISSEMINATED) LANGERHANS-CELL HISTIOCYTOSIS	2025	LETTERER-SIWE DISEASE*
C960	MULTIFOCAL AND MULTISYSTEMIC (DISSEMINATED) LANGERHANS-CELL HISTIOCYTOSIS	20250	LTR-SIWE UNSP XTRNDL ORG
C960	MULTIFOCAL AND MULTISYSTEMIC (DISSEMINATED) LANGERHANS-CELL HISTIOCYTOSIS	20251	LETTERER-SIWE DIS HEAD
C960	MULTIFOCAL AND MULTISYSTEMIC (DISSEMINATED) LANGERHANS-CELL HISTIOCYTOSIS	20252	LETTERER-SIWE DIS THORAX
C960	MULTIFOCAL AND MULTISYSTEMIC (DISSEMINATED) LANGERHANS-CELL HISTIOCYTOSIS	20253	LETTERER-SIWE DIS ABDOM
C960	MULTIFOCAL AND MULTISYSTEMIC (DISSEMINATED) LANGERHANS-CELL HISTIOCYTOSIS	20254	LETTERER-SIWE DIS AXILLA
C960	MULTIFOCAL AND MULTISYSTEMIC (DISSEMINATED) LANGERHANS-CELL HISTIOCYTOSIS	20255	LETTERER-SIWE DIS INGUIN
C960	MULTIFOCAL AND MULTISYSTEMIC (DISSEMINATED) LANGERHANS-CELL HISTIOCYTOSIS	20256	LETTERER-SIWE DIS PELVIC
C960	MULTIFOCAL AND MULTISYSTEMIC (DISSEMINATED) LANGERHANS-CELL HISTIOCYTOSIS	20257	LETTERER-SIWE DIS SPLEEN
C960	MULTIFOCAL AND MULTISYSTEMIC (DISSEMINATED) LANGERHANS-CELL HISTIOCYTOSIS	20258	LETTERER-SIWE DIS MULT
C962	MALIGNANT MAST CELL TUMOR	2026	MALIG MAST CELL TUMORS*
C962	MALIGNANT MAST CELL TUMOR	20260	MLG MAST UNSP XTRNDL ORG
C962	MALIGNANT MAST CELL TUMOR	20261	MAL MASTOCYTOSIS HEAD
C962	MALIGNANT MAST CELL TUMOR	20262	MAL MASTOCYTOSIS THORAX
C962	MALIGNANT MAST CELL TUMOR	20263	MAL MASTOCYTOSIS ABDOM
C962	MALIGNANT MAST CELL TUMOR	20264	MAL MASTOCYTOSIS AXILLA
C962	MALIGNANT MAST CELL TUMOR	20265	MAL MASTOCYTOSIS INGUIN
C962	MALIGNANT MAST CELL TUMOR	20266	MAL MASTOCYTOSIS PELVIC
C962	MALIGNANT MAST CELL TUMOR	20267	MAL MASTOCYTOSIS SPLEEN
C962	MALIGNANT MAST CELL TUMOR	20268	MAL MASTOCYTOSIS MULT
C858	Other specified types of non-Hodgkin lymphoma	2028	LYMPHOMAS NEC*
C8580	OTHER MALIGNANT LYMPHOMAS UNSPECIFIED SITE	20280	OTHER MALIGNANT LYMPHOMAS UNSPECIFIED SITE
C8581	OTHER SPECIFIED TYPES OF NON-HODGKIN LYMPHOMA, LYMPH NODES OF HEAD, FACE, AND NECK	20281	LYMPHOMAS NEC HEAD

C8582	OTHER SPECIFIED TYPES OF NON-HODGKIN LYMPHOMA, INTRATHORACIC LYMPH NODES	20282	LYMPHOMAS NEC THORAX
C8583	OTHER SPECIFIED TYPES OF NON-HODGKIN LYMPHOMA, INTRA-ABDOMINAL LYMPH NODES	20283	LYMPHOMAS NEC ABDOM
C8584	OTHER MALIGNANT LYMPHOMAS INVOLVING LYMPH NODES OF AXILLA AND UPPER LIMB	20284	LYMPHOMAS NEC AXILLA
C8585	OTHER MALIGNANT LYMPHOMAS INVOLVING LYMPH NODES OF INGUINAL REGION AND LOWER LIMB	20285	LYMPHOMAS NEC INGUIN
C8586	OTHER MALIGNANT LYMPHOMAS INVOLVING INTRAPELVIC LYMPH NODES	20286	LYMPHOMAS NEC PELVIC
C8587	OTHER MALIGNANT LYMPHOMAS INVOLVING SPLEEN	20287	LYMPHOMAS NEC SPLEEN
C8588	OTHER MALIGNANT LYMPHOMAS INVOLVING LYMPH NODES OF MULTIPLE SITES	20288	LYMPHOMAS NEC MULT
C96	Other and unspecified malignant neoplasms of lymphoid, hematopoietic and related tissue	2029	MAL NEO LYM/HIST TIS NEC*
C969	MALIGNANT NEOPLASM OF LYMPHOID, HEMATOPOIETIC AND RELATED TISSUE, UNSPECIFIED	20290	UNSP LYM UNSP XTRNDL ORG
C969	MALIGNANT NEOPLASM OF LYMPHOID, HEMATOPOIETIC AND RELATED TISSUE, UNSPECIFIED	20291	LYMPHOID MAL NEC HEAD
C969	MALIGNANT NEOPLASM OF LYMPHOID, HEMATOPOIETIC AND RELATED TISSUE, UNSPECIFIED	20292	LYMPHOID MAL NEC THORAX
C969	MALIGNANT NEOPLASM OF LYMPHOID, HEMATOPOIETIC AND RELATED TISSUE, UNSPECIFIED	20293	LYMPHOID MAL NEC ABDOM
C969	MALIGNANT NEOPLASM OF LYMPHOID, HEMATOPOIETIC AND RELATED TISSUE, UNSPECIFIED	20294	LYMPHOID MAL NEC AXILLA
C969	MALIGNANT NEOPLASM OF LYMPHOID, HEMATOPOIETIC AND RELATED TISSUE, UNSPECIFIED	20295	LYMPHOID MAL NEC INGUIN
C969	MALIGNANT NEOPLASM OF LYMPHOID, HEMATOPOIETIC AND RELATED TISSUE, UNSPECIFIED	20296	LYMPHOID MAL NEC PELVIC
C969	MALIGNANT NEOPLASM OF LYMPHOID, HEMATOPOIETIC AND RELATED TISSUE, UNSPECIFIED	20297	LYMPHOID MAL NEC SPLEEN
C969	MALIGNANT NEOPLASM OF LYMPHOID, HEMATOPOIETIC AND RELATED TISSUE, UNSPECIFIED	20298	LYMPHOID MAL NEC MULT
C90	Multiple myeloma and malignant plasma cell neoplasms	203	MULTIPLE MYELOMA ET AL*
C900	Multiple myeloma	2030	MULTIPLE MYELOMA*
C9000	MULTIPLE MYELOMA NOT HAVING ACHIEVED REMISSION	20300	MULT MYELM W/O REMISSION
C9001	MULTIPLE MYELOMA IN REMISSION	20301	MULT MYELM W REMISSION
C901	Plasma cell leukemia	2031	PLASMA CELL LEUKEMIA*
C9010	PLASMA CELL LEUKEMIA NOT HAVING ACHIEVED REMISSION	20310	PLSM CELL LEUK W/O RMSON
C9011	PLASMA CELL LEUKEMIA IN REMISSION	20311	PLSM CELL LEUK W RMSON
C833		2038	IMMUNOPROLIFERAT NEO NEC*
C8330		20380	OTH IMNPRFL NPL W/O RMSN
C8331		20381	OTH IMNPRFL NPL W RMSN
C91	Lymphoid leukemia	204	LYMPHOID LEUKEMIA*
C910	Acute lymphoblastic leukemia	2040	ACUTE LYMPHOID LEUKEMIA*
C9100	LYMPHOID LEUKEMIA ACUTE WITHOUT MENTION OF HAVING ACHIEVED REMISSION	20400	ACT LYM LEUK W/O RMSION
C9101	ACUTE LYMPHOBLASTIC LEUKEMIA, IN REMISSION	20401	ACT LYM LEUK W RMSION
C9102	ACUTE LYMPHOBLASTIC LEUKEMIA, IN RELAPSE	20402	
C911	Chronic lymphocytic leukemia	2041	CHR LYMPHOID LEUKEMIA*
C9110	CHRONIC LYMPHOCYTIC LEUKEMIA OF B-CELL TYPE NOT HAVING ACHIEVED REMISSION	20410	CHR LYM LEUK W/O RMSION
C9111	CHRONIC LYMPHOCYTIC LEUKEMIA OF B-CELL TYPE IN REMISSION	20411	CHR LYM LEUK W RMSION
C91Z	Subacute lymphocytic leukemia	2042	SUBAC LYMPHOID LEUKEMIA*
C91Z0	LYMPHOID LEUKEMIA, SUBACUTE, WITHOUT MENTION OF HAVING ACHIEVED REMISSION, FAILED REMISSION	20420	SBAC LYM LEUK W/O RMSION
C91Z1	LYMPHOID LEUKEMIA SUBACUTE IN REMISSION	20421	SBAC LYM LEUK W RMSION
C917	Other lymphoid leukemia	2048	LYMPHOID LEUKEMIA NEC*
C91Z0	OTHER LYMPHOID LEUKEMIA NOT HAVING ACHIEVED REMISSION	20480	OTH LYM LEUK W/O RMSION
C91Z1	OTHER LYMPHOID LEUKEMIA, IN REMISSION	20481	OTH LYM LEUK W RMSION
C91Z2	OTHER LYMPHOID LEUKEMIA, IN RELAPSE	20482	OTHER LYMPHOID LEUKEMIA, IN RELAPSE
C919	Lymphoid Leukemia, unspecified	2049	LYMPHOID LEUKEMIA NOS*

C9190	LYMPHOID LEUKEMIA, UNSPECIFIED NOT HAVING ACHIEVED REMISSION	20490	UNS LYM LEUK W/O RMSION
C9191	LYMPHOID LEUKEMIA, UNSPECIFIED, IN REMISSION	20491	UNS LYM LEUK W RMSION
C9192	LYMPHOID LEUKEMIA, UNSPECIFIED, IN RELAPSE	20492	UNSPECIFIED LYMPHOID LEUKEMIA, IN RELAPSE
C92	Myeloid Leukemia	205	MYELOID LEUKEMIA*
C920	ACUTE MYELOID LEUKEMIA	2050	ACUTE MYELOID LEUKEMIA*
C9200	ACUTE MYELOBLASTIC LEUKEMIA, NOT HAVING ACHIEVED REMISSION	20500	ACT MYL LEUK W/O RMSION
C9201	ACUTE MYELOBLASTIC LEUKEMIA, IN REMISSION	20501	ACT MYL LEUK W RMSION
C9202	ACUTE MYELOBLASTIC LEUKEMIA, IN RELAPSE	20502	MYELOID LEUKEMIA, ACUTE, IN RELAPSE
C921	Chronic myeloid leukemia, BCR/ABL-positive	2051	CHRONIC MYELOID LEUKEMIA*
C9210	CHRONIC MYELOID LEUKEMIA, BCR/ABL-POSITIVE, NOT HAVING ACHIEVED REMISSION	20510	CHR MYL LEUK W/O RMSION
C9211	CHRONIC MYELOID LEUKEMIA, BCR/ABL-POSITIVE, IN REMISSION	20511	CHR MYL LEUK W RMSION
C9212	CHRONIC MYELOID LEUKEMIA, BCR/ABL-POSITIVE, IN RELAPSE	2052	SUBACUT MYELOID LEUKEMIA*
C9220	ATYPICAL CHRONIC MYELOID LEUKEMIA, BCR/ABL-NEGATIVE, NOT HAVING ACHIEVED REMISSION	20520	SBAC MYL LEUK W/O RMSION
C9221	ATYPICAL CHRONIC MYELOID LEUKEMIA, BCR/ABL-NEGATIVE, IN REMISSION	20521	SBAC MYL LEUK W RMSION
C9222	ATYPICAL CHRONIC MYELOID LEUKEMIA, BCR/ABL-NEGATIVE, IN RELAPSE	20522	MYELOID LEUKEMIA, SUBACUTE, IN RELAPSE
C923	Myeloid sarcoma	2053	MYELOID SARCOMA*
C9230	MYELOID SARCOMA, NOT HAVING ACHIEVED REMISSION	20530	MYL SRCOMA W/O RMSION
C9231	MYELOID SARCOMA, IN REMISSION	20531	MYL SRCOMA W RMSION
C929	Myeloid leukemia, unspecified	2058	MYELOID LEUKEMIA NEC*
C92Z0	OTHER MYELOID LEUKEMIA NOT HAVING ACHIEVED REMISSION	20580	OTH MYL LEUK W/O RMSION
C92Z1	OTHER MYELOID LEUKEMIA, IN RELAPSE	20581	OTH MYL LEUK W RMSION
C92Z2	OTHER MYELOID LEUKEMIA, IN RELAPSE	20582	OTHER MYELOID LEUKEMIA, IN RELAPSE
C929	Myeloid leukemia, unspecified	2059	MYELOID LEUKEMIA NOS*
C9290	MYELOID LEUKEMIA, UNSPECIFIED, NOT HAVING ACHIEVED REMISSION	20590	UNS MYL LEUK W/O RMSION
C9291	MYELOID LEUKEMIA, UNSPECIFIED IN REMISSION	20591	UNS MYL LEUK W RMSION
C9292	MYELOID LEUKEMIA, UNSPECIFIED IN RELAPSE	20592	UNSPECIFIED MYELOID LEUKEMIA, IN RELAPSE
C93	MONOCYTIC LEUKEMIA	206	MONOCYTIC LEUKEMIA*
C930	ACUTE MONOCYTIC LEUKEMIA	2060	ACUTE MONOCYTIC LEUKEMIA*
C9300	ACUTE MONOBLASTIC/MONOCYTIC LEUKEMIA, NOT HAVING ACHIEVED REMISSION	20600	ACT MONO LEUK W/O RMSION
C9301	ACUTE MONOBLASTIC/MONOCYTIC LEUKEMIA, IN REMISSION	20601	ACT MONO LEUK W RMSION
C9302	ACUTE MONOBLASTIC/MONOCYTIC LEUKEMIA, IN RELAPSE	20602	MONOCYTIC LEUKEMIA, ACUTE, IN RELAPSE
C931	CHRONIC MYELOMONOCYTIC LEUKEMIA	2061	CHR MONOCYTIC LEUKEMIA*
C9310	CHRONIC MYELOMONOCYTIC LEUKEMIA NOT HAVING ACHIEVED REMISSION	20610	CHR MONO LEUK W/O RMSION
C9311	CHRONIC MYELOMONOCYTIC LEUKEMIA, IN REMISSION	20611	CHR MONO LEUK W RMSION
C9312	CHRONIC MYELOMONOCYTIC LEUKEMIA, IN RELAPSE	20612	MONOCYTIC LEUKEMIA, CHRONIC, IN RELAPSE
C932	Subacute monocytic leukemia	2062	SUBAC MONOCYTIC LEUKEMIA*
C9390	MONOCYTIC LEUKEMIA, UNSPECIFIED, NOT HAVING ACHIEVED REMISSION	20620	SBAC MONO LEUK W/O RMSON
C9391	MONOCYTIC LEUKEMIA, UNSPECIFIED IN REMISSION	20621	SBAC MONO LEUK W RMSION
C9392	MONOCYTIC LEUKEMIA, UNSPECIFIED IN RELAPSE	20622	MONOCYTIC LEUKEMIA, UNSPECIFIED IN RELAPSE
C929	Myeloid leukemia, unspecified	2068	MONOCYTIC LEUKEMIA NEC*
C93Z0	OTHER MONOCYTIC LEUKEMIA, NOT HAVING ACHIEVED REMISSION	20680	OTH MONO LEUK W/O RMSION
C93Z1	OTHER MONOCYTIC LEUKEMIA, IN REMISSION	20681	OTH MONO LEUK W RMSION

C93Z2	OTHER MONOCYTIC LEUKEMIA, IN RELAPSE	20682	OTHER MONOCYTIC LEUKEMIA, IN RELAPSE
C939	MONOCYTIC LEUKEMIA, UNSPECIFIED	2069	MONOCYTIC LEUKEMIA NOS*
C9390	MONOCYTIC LEUKEMIA, UNSPECIFIED, NOT HAVING ACHIEVED REMISSION	20690	UNS MONO LEUK W/O RMSION
C9391	MONOCYTIC LEUKEMIA, UNSPECIFIED IN REMISSION	20691	UNS MONO LEUK W RMSION
C9392	MONOCYTIC LEUKEMIA, UNSPECIFIED IN RELAPSE	20692	UNSPECIFIED MONOCYTIC LEUKEMIA, IN RELAPSE
C94	Other leukemia of specified cell type	207	OTHER SPECIFIED LEUKEMIA*
C940	Acute erythremia and erythroleukemia	2070	ACUTE ERYTHREMIA*
C9400	ACUTE ERYTHROID LEUKEMIA, NOT HAVING ACHIEVED REMISSION	20700	ACT ERTH/ERYLK W/O RMSON
C9401	ACUTE ERYTHROID LEUKEMIA, IN REMISSION	20701	ACT ERTH/ERYLK W RMSON
C9402	ACUTE ERYTHROID LEUKEMIA, IN RELAPSE	20702	ACUTE ERYTHREMIA AND ERYTHROLEUKEMIA, IN RELAPSE
D45	POLYCYTHEMIA VERA	2071	CHRONIC ERYTHREMIA*
D45	POLYCYTHEMIA VERA	20710	CHR ERYTHRM W/O REMISION
D45	POLYCYTHEMIA VERA	20711	CHR ERYTHRM W REMISION
D45	POLYCYTHEMIA VERA	20712	CHRONIC ERYTHREMIA, IN RELAPSE
C942	ACUTE MEGAKARYOBLASTIC LEUKEMIA	2072	MEGAKARYOCYTIC LEUKEMIA*
C9420	ACUTE MEGAKARYOBLASTIC LEUKEMIA NOT HAVING ACHIEVED REMISSION	20720	MGKRYCYT LEUK W/O RMSION
C9421	ACUTE MEGAKARYOBLASTIC LEUKEMIA, IN REMISSION	20721	MGKRYCYT LEUK W RMSION
C9422	ACUTE MEGAKARYOBLASTIC LEUKEMIA, IN RELAPSE	20722	MEGAKARYOCYTIC LEUKEMIA, IN RELAPSE
C948	Other specified leukemias	2078	SPECIFIED LEUKEMIA NEC*
C9480	OTHER SPECIFIED LEUKEMIAS NOT HAVING ACHIEVED REMISSION	20780	OTH SPF LEUK W/O REMSION
C9481	OTHER SPECIFIED LEUKEMIAS, IN REMISSION	20781	OTH SPF LEUK W REMSION
C9482	OTHER SPECIFIED LEUKEMIAS, IN RELAPSE	20782	OTHER SPECIFIED LEUKEMIA, IN RELAPSE
C95	LEUKEMIA OF UNSPECIFIED CELL TYPE	208	LEUKEMIA-UNSPECIF CELL*
C950	ACUTE LEUKEMIA OF UNSPECIFIED CELL TYPE	2080	ACT LEUK UNS CL W/O RMSN*
C9500	ACUTE LEUKEMIA OF UNSPECIFIED CELL TYPE NOT HAVING ACHIEVED REMISSION	20800	ACT LEUK UNS CL W/O RMSN
C9501	ACUTE LEUKEMIA OF UNSPECIFIED CELL TYPE, IN REMISSION	20801	ACT LEUK UNS CL W RMSON
C9502	ACUTE LEUKEMIA OF UNSPECIFIED CELL TYPE, IN RELAPSE	20802	LEUKEMIA OF UNSPECIFIED CELL TYPE, ACUTE, IN RELAPSE
C951	CHRONIC LEUKEMIA OF UNSPECIFIED CELL TYPE	2081	CHRONIC LEUKEMIA NOS*
C9510	CHRONIC LEUKEMIA OF UNSPECIFIED CELL TYPE NOT HAVING ACHIEVED REMISSION	20810	CHR LEUK UNS CL W/O RMSN
C9511	CHRONIC LEUKEMIA OF UNSPECIFIED CELL TYPE, IN REMISSION	20811	CHR LEUK UNS CL W RMSON
C9512	CHRONIC LEUKEMIA OF UNSPECIFIED CELL TYPE, IN RELAPSE	20812	LEUKEMIA OF UNSPECIFIED CELL TYPE, CHRONIC, IN RELAPSE
C959	LEUKEMIA, UNSPECIFIED	2082	SUBACUTE LEUKEMIA NOS*
C9590	LEUKEMIA, UNSPECIFIED NOT HAVING ACHIEVED REMISSION	20820	SBAC LEUK UNS CL W/O RMS
C9591	LEUKEMIA OF UNSPECIFIED CELL TYPE SUBACUTE IN REMISSION	20821	SBAC LEUK UNS CL W RMSON
C9592	LEUKEMIA OF UNSPECIFIED CELL TYPE, SUBACUTE, IN RELAPSE	20822	LEUKEMIA OF UNSPECIFIED CELL TYPE, SUBACUTE, IN RELAPSE
C959	LEUKEMIA, UNSPECIFIED	2088	LEUKEMIA-UNSPEC CELL NEC*
C9590	LEUKEMIA, UNSPECIFIED NOT HAVING ACHIEVED REMISSION	20880	OTH LEUK UNS CL W/O RMSN
C9591	LEUKEMIA, UNSPECIFIED, IN REMISSION	20881	OTH LEUK UNS CL W RMSON
C9592	LEUKEMIA, UNSPECIFIED, IN RELAPSE	20882	OTHER LEUKEMIA OF UNSPECIFIED CELL TYPE, IN RELAPSE
C959	LEUKEMIA, UNSPECIFIED	2089	LEUKEMIA-UNSPEC CELL NOS*

C9590	LEUKEMIA, UNSPECIFIED NOT HAVING ACHIEVED REMISSION	20890	LEUKEMIA NOS W/O REMSION
C9591	LEUKEMIA, UNSPECIFIED, IN REMISSION	20891	LEUKEMIA NOS W REMISSION
C9592	LEUKEMIA, UNSPECIFIED, IN RELAPSE	20892	UNSPECIFIED LEUKEMIA IN RELAPSE
D00	Carcinoma in situ of oral cavity, esophagus and stomach	230	CA IN SITU DIGESTIVE ORG*
D000	CARCINOMA IN SITU OF ORAL CAVITY, UNSPECIFIED SITE	2300	CA IN SITU ORAL CAV/PHAR
D001	CARCINOMA IN SITU OF ESOPHAGUS	2301	CA IN SITU ESOPHAGUS
D002	CARCINOMA IN SITU OF STOMACH	2302	CA IN SITU STOMACH
D010	CARCINOMA IN SITU OF COLON	2303	CA IN SITU COLON
D012	CARCINOMA IN SITU OF RECTUM	2304	CA IN SITU RECTUM
D013	CARCINOMA IN SITU OF ANUS AND ANAL CANAL	2305	CA IN SITU ANAL CANAL
D013	CARCINOMA IN SITU OF ANUS AND ANAL CANAL	2306	CA IN SITU ANUS NOS
D014	CARCINOMA IN SITU OF OTHER AND UNSPECIFIED PARTS OF INTESTINE	2307	CA IN SITU BOWEL NEC/NOS
D015	CARCINOMA IN SITU OF LIVER, GALLBLADDER AND BILE DUCTS	2308	CA IN SITU LIVER/BILIARY
D019	CARCINOMA IN SITU OF DIGESTIVE ORGAN, UNSPECIFIED	2309	CA IN SITU GI NEC/NOS
D02	CARCINOMA IN SITU OF MIDDLE EAR AND RESPIRATORY SYSTEM	231	CA IN SITU RESPIRATORY*
D020	CARCINOMA IN SITU OF LARYNX	2310	CA IN SITU LARYNX
D021	CARCINOMA IN SITU OF TRACHEA	2311	CA IN SITU TRACHEA
D022	CARCINOMA IN SITU OF BRONCHUS AND LUNG	2312	CA IN SITU BRONCHUS/LUNG
D023	CARCINOMA IN SITU OF OTHER PARTS OF RESPIRATORY SYSTEM	2318	CA IN SITU RESP SYS NEC
D024	CARCINOMA IN SITU OF RESPIRATORY SYSTEM, UNSPECIFIED	2319	CA IN SITU RESP SYS NOS
D040	CARCINOMA IN SITU OF SKIN OF LIP	2320	CA IN SITU SKIN LIP
D0410	CARCINOMA IN SITU OF SKIN OF UNSPECIFIED EYELID, INCLUDING CANTHUS	2321	CA IN SITU EYELID
D0420	CARCINOMA IN SITU OF SKIN OF UNSPECIFIED EAR AND EXTERNAL AURICULAR CANAL	2322	CA IN SITU SKIN EAR
D0430	CARCINOMA IN SITU OF SKIN OF UNSPECIFIED PART OF FACE	2323	CA IN SITU SKIN FACE NEC
D044	CARCINOMA IN SITU OF SKIN OF SCALP AND NECK	2324	CA IN SITU SCALP
D045	CARCINOMA IN SITU OF SKIN OF TRUNK	2325	CA IN SITU SKIN TRUNK
D0460	CARCINOMA IN SITU OF SKIN OF UNSPECIFIED UPPER LIMB, INCLUDING SHOULDER	2326	CA IN SITU SKIN ARM
D0470	CARCINOMA IN SITU OF SKIN OF UNSPECIFIED LOWER LIMB, INCLUDING HIP	2327	CA IN SITU SKIN LEG
D048	CARCINOMA IN SITU OF SKIN OF OTHER SITES	2328	CA IN SITU SKIN NEC
D049	CARCINOMA IN SITU OF SKIN, UNSPECIFIED	2329	CA IN SITU SKIN NOS
D059	CARCINOMA IN SITU OF BREAST, UNSPECIFIED	233	CA IN SITU BREAST/GU*
D0590	UNSPECIFIED TYPE OF CARCINOMA IN SITU OF UNSPECIFIED BREAST	2330	CA IN SITU BREAST
D069	CARCINOMA IN SITU OF CERVIX, UNSPECIFIED	2331	CA IN SITU CERVIX UTERI
D070	CARCINOMA IN SITU OF ENDOMETRIUM	2332	CA IN SITU UTERUS NEC
D073	CARCINOMA IN SITU OF OTHER AND UNSPECIFIED GENITAL ORGANS	2333	CA IN SITU FEM GEN NEC
D0730	CARCINOMA IN SITU OF UNSPECIFIED FEMALE GENITAL ORGANS	23330	CARCINOMA IN SITU OF OTHER AND UNSPECIFIED FEMALE GENITAL ORGANS; UNSPECIFIED FEMALE GENITAL ORGAN
D072	CARCINOMA IN SITU OF VAGINA	23331	CARCINOMA IN SITU OF OTHER AND UNSPECIFIED FEMALE GENITAL ORGANS; VAGINA
D071	CARCINOMA IN SITU OF VULVA	23332	CARCINOMA IN SITU OF OTHER AND UNSPECIFIED FEMALE GENITAL ORGANS; VULVA

D0739	CARCINOMA IN SITU OF OTHER FEMALE GENITAL ORGANS	23339	CARCINOMA IN SITU OF OTHER AND UNSPECIFIED FEMALE GENITAL ORGANS; OTHER FEMALE GENITAL ORGAN
D075	CARCINOMA IN SITU OF PROSTATE	2334	CA IN SITU PROSTATE
D074	CARCINOMA IN SITU OF PENIS	2335	CA IN SITU PENIS
D0760	CARCINOMA IN SITU OF UNSPECIFIED MALE GENITAL ORGANS	2336	CA IN SITU MALE GEN NEC
D090	CARCINOMA IN SITU OF BLADDER	2337	CA IN SITU BLADDER
D091	CARCINOMA IN SITU OF OTHER AND UNSPECIFIED URINARY ORGANS	2339	CA IN SITU URINARY NEC
D09	CARCINOMA IN SITU OF OTHER AND UNSPECIFIED SITES	234	CA IN SITU NEC/NOS*
D0920	CARCINOMA IN SITU OF UNSPECIFIED EYE	2340	CA IN SITU EYE
D098	CARCINOMA IN SITU OF OTHER SPECIFIED SITES	2348	CA IN SITU NEC
D099	CARCINOMA IN SITU, UNSPECIFIED	2349	CA IN SITU NOS
D37	NEOPLASM OF UNCERTAIN OR UNKNOWN BEHAVIOR OF ORAL CAVITY AND DIGESTIVE ORGANS	235	UNC BEHAV NEO GI/RESP*
D37039	NEOPLASM OF UNCERTAIN BEHAVIOR OF THE MAJOR SALIVARY GLANDS, UNSPECIFIED	2350	UNC BEHAV NEO SALIVARY
D370	Neoplasm of uncertain behavior of lip, oral cavity and pharynx	2351	UNC BEHAV NEO ORAL/PHAR
D378	Neoplasm of uncertain behavior of other specified digestive organs	2352	UNC BEHAV NEO INTESTINE
D376	NEOPLASM OF UNCERTAIN BEHAVIOR OF LIVER, GALLBLADDER AND BILE DUCTS	2353	UNC BEHAV NEO LIVER
D484	NEOPLASM OF UNCERTAIN BEHAVIOR OF PERITONEUM	2354	UNC BEHAV NEO PERITONEUM
D379	NEOPLASM OF UNCERTAIN BEHAVIOR OF DIGESTIVE ORGAN, UNSPECIFIED	2355	UNC BEHAV NEO GI NEC
D380	NEOPLASM OF UNCERTAIN BEHAVIOR OF LARYNX	2356	UNC BEHAV NEO LARYNX
D381	NEOPLASM OF UNCERTAIN BEHAVIOR OF TRACHEA, BRONCHUS AND LUNG	2357	UNC BEHAV NEO LUNG
D385	NEOPLASM OF UNCERTAIN BEHAVIOR OF OTHER RESPIRATORY ORGANS	2358	UNC BEHAV NEO PLEURA
D386	NEOPLASM OF UNCERTAIN BEHAVIOR OF RESPIRATORY ORGAN, UNSPECIFIED	2359	UNC BEHAV NEO RESP NEC
D39	Neoplasm of uncertain behavior of female genital organs	236	UNC BEHAV NEO GU*
D390	NEOPLASM OF UNCERTAIN BEHAVIOR OF UTERUS	2360	UNCERT BEHAV NEO UTERUS
D392	NEOPLASM OF UNCERTAIN BEHAVIOR OF PLACENTA	2361	UNC BEHAV NEO PLACENTA
D3910	NEOPLASM OF UNCERTAIN BEHAVIOR OF UNSPECIFIED OVARY	2362	UNC BEHAV NEO OVARY
D399	NEOPLASM OF UNCERTAIN BEHAVIOR OF FEMALE GENITAL ORGAN, UNSPECIFIED	2363	UNC BEHAV NEO FEMALE NEC
D401	NEOPLASM OF UNCERTAIN BEHAVIOR OF TESTIS	2364	UNC BEHAV NEO TESTIS
D400	NEOPLASM OF UNCERTAIN BEHAVIOR OF PROSTATE	2365	UNC BEHAV NEO PROSTATE
D409	NEOPLASM OF UNCERTAIN BEHAVIOR OF MALE GENITAL ORGAN, UNSPECIFIED	2366	UNC BEHAV NEO MALE NEC
D414	NEOPLASM OF UNCERTAIN BEHAVIOR OF BLADDER	2367	UNC BEHAV NEO BLADDER
D41	NEOPLASM OF UNCERTAIN OR UNKNOWN BEHAVIOR OF URINARY ORGANS	2369	UNC BEHAV NEO OTH URINAR*
D419	NEOPLASM OF UNCERTAIN BEHAVIOR OF UNSPECIFIED URINARY ORGAN	23690	UNC BEHAV NEO URINAR NOS
D4100	NEOPLASM OF UNCERTAIN BEHAVIOR OF UNSPECIFIED KIDNEY	23691	UNC BEHAV NEO KIDNEY
D418	NEOPLASM OF UNCERTAIN BEHAVIOR OF OTHER SPECIFIED URINARY ORGANS	23699	UNC BEHAV NEO URINAR NEC
D44	NEOPLASM OF UNCERTAIN BEHAVIOR OF OF ENDOCRINE GLANDS	237	UNCER NEO ENDOCRINE/NERV*
D44	NEOPLASM OF UNCERTAIN BEHAVIOR OF OF ENDOCRINE GLANDS	2370	UNC BEHAV NEO PITUITARY
D445	NEOPLASM OF UNCERTAIN BEHAVIOR OF PINEAL GLAND	2371	UNC BEHAV NEO PINEAL
D4410	NEOPLASM OF UNCERTAIN BEHAVIOR OF UNSPECIFIED ADRENAL GLAND	2372	UNC BEHAV NEO ADRENAL
D447	NEOPLASM OF UNCERTAIN BEHAVIOR OF AORTIC BODY AND OTHER PARAGANGLIA	2373	UNC BEHAV NEO PARAGANG
D449	NEOPLASM OF UNCERTAIN BEHAVIOR OF UNSPECIFIED ENDOCRINE GLAND	2374	UNCER NEO ENDOCRINE NEC
D43	NEOPLASM OF UNCERTAIN OR UNKNOWN BEHAVIOR OF BRAIN AND CENTRAL NERVOUS SYSTEM	2375	UNC BEH NEO BRAIN/SPINAL
D429	NEOPLASM OF UNCERTAIN BEHAVIOR OF MENINGES, UNSPECIFIED	2376	UNC BEHAV NEO MENINGES

Q85	Phakomatoses, not elsewhere classified	2377	NEUROFIBROMATOSIS*
Q8500	NEUROFIBROMATOSIS, UNSPECIFIED	23770	NEUROFIBROMATOSIS NOS
Q8501	NEUROFIBROMATOSIS, TYPE 1	23771	NEUROFIBROMATOSIS TYPE I
Q8502	NEUROFIBROMATOSIS, TYPE 2	23772	NEUROFIBROMATOSIS TYP II
Q8503	SCHWANNOMATOSIS	23773	SCHWANNOMATOSIS
Q8509	OTHER NEUROFIBROMATOSIS	23779	OTHER NEUROFIBROMATOSIS
D439	NEOPLASM OF UNCERTAIN BEHAVIOR OF CENTRAL NERVOUS SYSTEM, UNSPECIFIED	2379	UNC BEH NEO NERV SYS NEC
D48	Neoplasm of uncertain behavior of other and unspecified sites	238	UNC BEHAV NEO NEC/NOS*
D480	NEOPLASM OF UNCERTAIN BEHAVIOR OF BONE AND ARTICULAR CARTILAGE	2380	UNC BEHAV NEO BONE
D481	NEOPLASM OF UNCERTAIN BEHAVIOR OF CONNECTIVE AND OTHER SOFT TISSUE	2381	UNC BEHAV NEO SOFT TISSU
D485	NEOPLASM OF UNCERTAIN BEHAVIOR OF SKIN	2382	UNC BEHAV NEO SKIN
D4860	NEOPLASM OF UNCERTAIN BEHAVIOR OF UNSPECIFIED BREAST	2383	UNC BEHAV NEO BREAST
D45	POLYCYTHEMIA VERA	2384	POLYCYTHEMIA VERA
D470	HISTIOCYTIC AND MAST CELL TUMORS OF UNCERTAIN BEHAVIOR	2385	MASTOCYTOMA NOS
D47Z9	OTHER SPECIFIED NEOPLASMS OF UNCERTAIN BEHAVIOR OF LYMPHOID, HEMATOPOIETIC AND RELATED TISSUE	2386	PLASMACYTOMA NOS
D47Z9	OTHER SPECIFIED NEOPLASMS OF UNCERTAIN BEHAVIOR OF LYMPHOID, HEMATOPOIETIC AND RELATED TISSUE	2387	LYMPHOPROLIFERAT DIS NOS
D473	ESSENTIAL (HEMORRHAGIC) THROMBOCYTHEMIA	23871	ESSENTIAL THROMBOCYTHEMIA
D46	Myelodysplastic syndromes	23872	LOW GRADE MYELODYSPLASTIC SYNDROME LESIONS
D4622	REFRACTORY ANEMIA WITH EXCESS OF BLASTS 2	23873	HIGH GRADE MYELODYSPLASTIC SYNDROME LESIONS
D487	NEOPLASM OF UNCERTAIN BEHAVIOR OF OTHER SPECIFIED SITES	2388	UNCERT BEHAVIOR NEO NEC
D489	NEOPLASM OF UNCERTAIN BEHAVIOR, UNSPECIFIED	2389	UNCERT BEHAVIOR NEO NOS
D49	Neoplasms of unspecified behavior	239	UNSPECIFIED NEOPLASM*
D490	NEOPLASM OF UNSPECIFIED BEHAVIOR OF DIGESTIVE SYSTEM	2390	DIGESTIVE NEOPLASM NOS
D491	NEOPLASM OF UNSPECIFIED BEHAVIOR OF RESPIRATORY SYSTEM	2391	RESPIRATORY NEOPLASM NOS
D492	NEOPLASM OF UNSPECIFIED BEHAVIOR OF BONE, SOFT TISSUE, AND SKIN	2392	BONE/SKIN NEOPLASM NOS
D493	NEOPLASM OF UNSPECIFIED BEHAVIOR OF BREAST	2393	BREAST NEOPLASM NOS
D494	NEOPLASM OF UNSPECIFIED BEHAVIOR OF BLADDER	2394	BLADDER NEOPLASM NOS
D495	NEOPLASM OF UNSPECIFIED BEHAVIOR OF OTHER GENITOURINARY ORGANS	2395	OTHER GU NEOPLASM NOS
D496	NEOPLASM OF UNSPECIFIED BEHAVIOR OF BRAIN	2396	BRAIN NEOPLASM NOS
D497	NEOPLASM OF UNSPECIFIED BEHAVIOR OF ENDOCRINE GLANDS AND OTHER PARTS OF NERVOUS SYSTEM	2397	ENDOCRINE/NERV NEO NOS
D498	Neoplasm of unspecified behavior of other specified sites	2398	NEOPLASM NOS, SITE NEC
D499	NEOPLASM OF UNSPECIFIED BEHAVIOR OF UNSPECIFIED SITE	2399	NEOPLASM NOS

Table S2e. ICD-9 and ICD-19 Codes for Kidney disease or other complications due to renal dialysis

ICD-10 Codes	ICD-9 Codes	Description
N00	580	ACUTE NEPHRITIS
N003	5800	AC PROLIFERAT NEPHRITIS
N013	5804	AC RAPIDLY PROGR NEPHRIT
N008	5808	AC NEPHRITIS W OTH LES
N08	58081	AC NEPHRITIS IN OTH DIS
N008	58089	ACUTE NEPHRITIS NEC
N009	5809	ACUTE NEPHRITIS NOS
N04	581	NEPHROTIC SYNDROME
N044	5810	NEPHROTIC SYN, PROLIFER
N022	5811	EPIMEMBRANOUS NEPHRITIS
N043	5812	MEMBRANOPROLIF NEPHROSIS
N040	5813	MINIMAL CHANGE NEPHROSIS
N041	5818	NEPHROTIC SYN W OTH LES
N08	58181	NEPHROTIC SYN IN OTH DIS
N048	58189	NEPHROTIC SYNDROME NEC
N049	5819	NEPHROTIC SYNDROME NOS
N03	582	CHRONIC NEPHRITIS
N032	5820	CHR PROLIFERAT NEPHRITIS
N033	5821	CHR MEMBRANOUS NEPHRITIS
N035	5822	CHR MEMBRANOPROLIF NEPHR
N038	5824	CHR RAPID PROGR NEPHRIT
N038	5828	CHR NEPHRITIS W OTH LES
N08	58281	CHR NEPHRITIS IN OTH DIS
N038	58289	CHRONIC NEPHRITIS NEC
N039	5829	CHRONIC NEPHRITIS NOS
N059	583	NEPHRITIS NOS
N059	5830	PROLIFERAT NEPHRITIS NOS
N052	5831	MEMBRANOUS NEPHRITIS NOS
N055	5832	MEMBRANOPROLIF NEPHR NOS
N059	5834	RAPIDLY PROG NEPHRIT NOS
N171	5836	RENAL CORT NECROSIS NOS
N172	5837	NEPHR NOS/MEDULL NECROS
N059	5838	NEPHRITIS NOS W OTH LES
N08	58381	NEPHRITIS NOS IN OTH DIS
N058	58389	NEPHRITIS NEC
N059	5839	NEPHRITIS NOS
N17	584	ACUTE RENAL FAILURE
N170	5845	LOWER NEPHRON NEPHROSIS
N171	5846	AC RENAL FAIL, CORT NECR
N172	5847	AC REN FAIL, MEDULL NECR

N178	5848	AC RENAL FAILURE NEC
N179	5849	ACUTE RENAL FAILURE NOS
N18	585	CHRONIC RENAL FAILURE
N181	5851	Chronic kidney disease, stage 1
N182	5852	Chronic kidney disease, stage 2 (mild)
N183	5853	Chronic kidney disease, stage 3 (moderate)
N1830	5853	Chronic kidney disease, stage 3 unspecified
N1831	5853	Chronic kidney disease, stage 3a
N1832	5853	Chronic kidney disease, stage 3b
N184	5854	Chronic kidney disease, stage 4 (severe)
N185	5855	Chronic kidney disease, stage 5
N186	5856	End stage renal disease
N189	5859	Chronic kidney disease, unspecified
N19	586	RENAL FAILURE NOS
N269	587	RENAL SCLEROSIS NOS
N25	588	IMPAIRED RENAL FUNCTION
N250	5880	RENAL OSTEODYSTROPHY
N251	5881	NEPHROGEN DIABETES INSIP
N2589	5888	IMPAIRED RENAL FUNCT NEC
N259	5889	IMPAIRED RENAL FUNCT NOS
N27	589	SMALL KIDNEY
N270	5890	UNILATERAL SMALL KIDNEY
N271	5891	BILATERAL SMALL KIDNEYS
N279	5899	SMALL KIDNEY NOS
N11	590	CHRONIC NEPHRITIS
N110	5900	CHRONIC PYELONEPHRITIS
N110	59000	CHR PYELONEPHRITIS NOS
N118	59001	CHR PYELONEPH W MED NECR
N10	5901	ACUTE PYELONEPHRITIS
N10	59010	AC PYELONEPHRITIS NOS
N10	59011	AC PYELONEPHR W MED NECR
N151	5902	RENAL/PERIRENAL ABSCESS
N2884	5903	PYELOURETERITIS CYSTICA
N12	5908	OTHER PYELONEPHRITIS
N12	59080	PYELONEPHRITIS NOS
N12	59081	PYELONEPHRIT IN OTH DIS
N159	5909	INFECTION OF KIDNEY NOS
N130	591	HYDRONEPHROSIS
N131	591	HYDRONEPHROSIS
N132	591	HYDRONEPHROSIS
N133	591	HYDRONEPHROSIS
N136	591	HYDRONEPHROSIS
N20	592	RENAL/URETERAL CALCULUS
N200	5920	CALCULUS OF KIDNEY

N201	5921	CALCULUS OF URETER
N209	5929	URINARY CALCULUS NOS
N288	593	OTH RENAL & URETERAL DIS
N2883	5930	NEPHROPTOSIS
N2881	5931	HYPERTROPHY OF KIDNEY
N281	5932	CYST OF KIDNEY, ACQUIRED
N135	5933	STRICTURE OF URETER
N138	5934	URETERIC OBSTRUCTION NEC
N134	5935	HYDROURETER
R802	5936	POSTURAL PROTEINURIA
N137	5937	VESICOURETERAL REFLUX
N1370	59370	VESCOURETRL RFLUX UNSPCF
N13721	59371	VSCURT RFLX NPHT UNILTRL
N13722	59372	VSCOURTL RFLX NPHT BLTRL
N13729	59373	VSCOURTL RFLX W NPHT NOS
N28	5938	OTH RENAL & URETERAL DIS
N280	59381	RENAL VASCULAR DISORDER
N2889	59382	URETERAL FISTULA
N2889	59389	RENAL & URETERAL DIS NEC
N289	5939	RENAL & URETERAL DIS NOS
Q610	7531	CYSTIC KIDNEY DISEASE
Q611	7531	CYSTIC KIDNEY DISEASE
Q612	75310	CYSTIC KIDNEY DISEAS NOS
Q613	75311	CONGENITAL RENAL CYST
Q614	75312	POLYCYSTIC KIDNEY NOS
Q615	75313	POLYCYST KID-AUTOSOM DOM
Q618	75314	POLYCYST KID-AUTOSOM REC
Q619	75315	RENAL DYSPLASIA
Q615	75316	MEDULLARY CYSTIC KIDNEY
Q615	75317	MEDULLARY SPONGE KIDNEY
Q615	75319	CYSTIC KIDNEY DISEAS NEC
Q630	7533	KIDNEY ANOMALY NEC
Q631	7533	KIDNEY ANOMALY NEC
Q632	7533	KIDNEY ANOMALY NEC
Q633	7533	KIDNEY ANOMALY NEC
Q638	7533	KIDNEY ANOMALY NEC
T82898A	99673	COMP-REN DIALYS DEV/GRFT
T861	99681	COMPL KIDNEY TRANSPLANT
Z992	3995	HEMODIALYSIS
Z940	556	KIDNEY TRANSPLANT
Z940	5561	RENAL AUTOTRANSPLANT
Z940	5569	KIDNEY TRANSPLANT NEC

Table S3. Baseline characteristics of participants eligible for Medicare and enrolled for Medicare Part-D by three comparison groups in 2007

	Participants, No (%)				P value
	Chlorthalidone	Amlodipine	Lisinopril	Total	
Eligible number of participants	3637	2189	2181	8007	
Age, mean [range], years	67.8 [55-94]	67.9 [55-89]	67.7 [55-90]	67.8 [55-94]	0.231
55-64	1162 (31.9)	694 (31.7)	706 (32.4)	2562 (32.0)	0.786
65-69	1183 (32.5)	693 (31.7)	716 (32.8)	2592 (32.4)	
70 or older	1292 (35.5)	802 (36.6)	759 (34.8)	2651 (35.2)	
Sex					
Women	2355 (64.8)	1442 (65.9)	1399 (64.1)	5196 (64.9)	0.474
Men	1282 (35.2)	747 (34.1)	782 (35.9)	2811 (35.1)	
Race/ethnicity					
Black	1395 (38.4)	826 (37.7)	802 (36.8)	3023 (37.8)	0.483
Non-Black	2242 (61.6)	1363 (62.3)	1379 (63.2)	4984 (62.2)	
Hispanic ethnicity*					
Hispanic	898 (24.8)	509 (23.4)	574 (26.5)	1981 (24.9)	0.063
Non-Hispanic	2723 (75.2)	1663 (76.6)	1591 (73.5)	5977 (75.1)	
Education, mean (SD), years*	10.4 (4.1)	10.3 (4.0)	10.3 (4.3)	10.4 (4.1)	<0.001
Antihypertensive treatment					
Treated (prior to baseline)	3303 (90.8)	1974 (90.2)	1984 (91.0)	7261 (90.7)	0.623

Untreated	334 (9.2)	215 (9.8)	197 (9.0)	746 (9.3)	
Aspirin use at baseline*					
Yes aspirin used	1160 (32.4)	699 (32.3)	704 (32.7)	2563 (32.5)	0.964
On pravastatin	506 (13.9)	301 (13.8)	309 (14.2)	1116 (13.9)	0.922
Women taking estrogen*	417 (18.0)	238 (16.7)	219 (15.9)	874 (17.1)	0.224
HDL, mean (SD), mg/dl*	48.8 (14.6)	48.8 (14.5)	49.0 (14.8)	48.8 (14.6)	0.673
LDL, mean (SD), mg/dl*	137.9 (37.8)	136.6 (36.9)	137.3 (37.0)	137.4 (37.3)	0.339
HDL <35 mg/dl	386 (10.6)	240 (11.0)	227 (10.4)	853 (10.7)	0.833
GFR, mean (SD)*	77.9 (18.2)	77.9 (18.1)	77.9 (18.4)	77.9 (18.2)	0.821
Diabetes classification					
Diabetic	1280 (35.2)	761 (34.8)	783 (35.9)	2824 (35.3)	0.728
Non-diabetic	2357 (64.8)	1428 (65.2)	1398 (64.1)	5183 (64.7)	
Cigarette smoking					
Smoker (current)	605 (16.6)	381 (17.4)	338 (15.5)	1324 (16.5)	0.234
Nonsmoker (non/former)	3032 (83.4)	1808 (82.6)	1842 (84.5)	6682 (83.5)	
History of CHD*	848 (23.5)	484 (22.3)	473 (21.9)	1805 (22.7)	0.326
Atherosclerotic CVD (yes if any of 4 below)	1870 (51.4)	1095 (50.0)	1083 (49.7)	4048 (50.6)	0.362
History MI or stroke	735 (20.2)	463 (21.2)	422 (19.3)	1620 (20.2)	0.333
History coronary revascularization	406 (11.2)	211 (9.6)	241 (11.0)	858 (10.7)	0.160
Other atherosclerotic CVD	914 (25.1)	553 (25.3)	518 (23.8)	1985 (24.8)	0.416
ST-T wave*	383 (10.6)	204 (9.4)	249 (11.5)	836 (10.5)	0.074
LVH by Minnesota code*	134 (4.4)	94 (5.1)	76 (4.2)	304 (4.5)	0.329

SBP, mean (SD), mmHg	145.5 (12.8)	145.6 (13.1)	145.5 (12.9)	145.5 (12.9)	0.445
DBP, mean (SD), mmHg	83.8 (8.7)	83.8 (8.8)	83.5 (8.7)	83.7 (8.8)	0.814
Final SBP, mean (SD), mmHg	136.1 (15.9)	135.9 (15.3)	137.8 (17.5)	136.5 (16.2)	<0.001
BMI, mean (SD), mg/kg^{2}*	30.3 (6.3)	30.1 (6.1)	30.1 (6.1)	30.2 (6.2)	0.032
Obesity*	1594 (43.9)	955 (43.7)	976 (44.9)	3525 (44.1)	0.717
Lipid trial participants	987 (27.1)	575 (26.3)	572 (26.2)	2134 (26.7)	0.668

*Reduced denominator Chlorthalidone/Amlodipine/Lisinopril available for Aspirin: C=3581/A=2162/L=2154; BMI: C=3627/A=2183/L=2175; Education: C=3374/A=2025/L=2011; Estrogen: C=2312/A=1423/L=1378; GFR: C=3497/A=2083/L=2075; HDL: C=3484/A=2078/L=2067; Hispanic: C=3621/A=2172/L=2165; History of CHD: C=3611/A=2170/L=2160; LDL: C=3314/A=1971/L=1962; LVH by Minnesota code: C=3065/A=1837/L=1827; Obesity: C=3627/A=2183/L=2175; ST-T wave: C=3608/A=2165/L=2156.

Table S4a. Patterns of antihypertensive drug utilization from Medicare Part-D drug coverage in 2007, by 3 arms and added-on drugs (Black)

**Total Black Participants: N=3024**	**N (%) of antihypertensive drug who received the study class drug**				**N (%) of antihypertensive drug who did not receive the study trial drug**			
**Diuretic= Thiazide/thiazide-type**<br>[Randomized to Chlorthalidone in trial: 1395 (46.1)]	1) any diuretic<br>(1 drug)	2) any diuretic plus 1 drug<br>(2 drugs)	3) any diuretic plus 2 drugs<br>(3 drugs)	4) any diuretic plus ≥3 drugs<br>(4+ drugs)	1) no diuretic<br>(on 1 drug)	2) no diuretic<br>(on 2 drugs)	3) no diuretic<br>(on 3 drugs)	4) no diuretic<br>(on 4+ drugs)
Aldosterone antagonists †		0 (0)	1 (0.1)	16 (1.1)	2 (0.1)	1 (0.1)	11 (0.8)	32 (2.3)
Alpha 1 adrenergic receptor agonist (selective; **α-blocker**)		0 (0)	6 (0.4)	24 (1.7)	1 (0.1)	1 (0.1)	8 (0.6)	13 (0.9)
Angiotensin-converting enzyme (**ACE**) inhibitor/ Angiotensin II receptor blockers (**ARB)**		76 (5.4)	171 (12.3)	259 (18.6)	50 (3.6)	108 (7.7)	127 (9.1)	140 (10)
Arteriolar vasodilators		0 (0)	4 (0.3)	30 (2.2)	1 (0.1)	2 (0.1)	10 (0.7)	38 (2.7)
Autonomic ganglionic vasodilators		0 (0)	0 (0)	0 (0)	0 (0)	0 (0)	0 (0)	0 (0)
Beta adrenergic blockers (**β-blockers**)		34 (2.4)	85 (6.1)	224 (16.1)	24 (1.7)	78 (5.6)	112 (8)	132 (9.5)
Calcium channel blocker (**CCB**)-dihydropyridine/non-dihydropyridine		17 (1.2)	110 (7.9)	218 (15.6)	56 (4)	92 (6.6)	106 (7.6)	126 (9)
Central alpha 2 adrenergic agonists		2 (0.1)	6 (0.4)	60 (4.3)	5 (0.4)	9 (0.6)	24 (1.7)	48 (3.4)
Diuretics: thiazide/thiazide-type	32 (2.3)	148 (10.6)	212 (15.2)	277 (19.9)	0 (0)	0 (0)	0 (0)	0 (0)
Diuretics: loop		5 (0.4)	21 (1.5)	96 (6.9)	12 (0.9)	64 (4.6)	101 (7.2)	128 (9.2)
Diuretics: potassium-sparing†		14 (1)	20 (1.4)	42 (3)	1 (0.1)	3 (0.2)	2 (0.1)	4 (0.3)
Peripheral adrenergic neuron antagonist†		0 (0)	0 (0)	1 (0.1)	0 (0)	0 (0)	0 (0)	1 (0.1)
Renin inhibitors		0 (0)	0 (0)	8 (0.6)	0 (0)	0 (0)	0 (0)	0 (0)
**Total participants**	32 (2.3)	148 (10.6)	212 (15.2)	277 (19.9)	152 (10.9)	179 (12.8)	167 (12)	153 (11)
**Calcium channel blocker (CCB)**<br>[Randomized to Amlodipine in trial: 827 (27.3)]	1) any CCB<br>(1 drug)	2) any CCB plus 1 drug	3) any CCB plus 2 drugs	4) any CCB plus ≥3 drugs	1) no CCB (on 1 drug)	2) no CCB (on 2 drugs)	3) no CCB (on 3 drugs)	4) no CCB (on 4+ drugs)

		(2 drugs)	(3 drugs)	(4+ drugs)				
Aldosterone antagonists †		0 (0)	1 (0.1)	15 (1.8)	0 (0)	2 (0.2)	5 (0.6)	9 (1.1)
Alpha 1 adrenergic receptor agonist (selective; **α-blocker**)		1 (0.1)	2 (0.2)	20 (2.4)	1 (0.1)	4 (0.5)	5 (0.6)	8 (1)
Angiotensin-converting enzyme (**ACE**) inhibitor/Angiotensin II receptor blockers (**ARB)**		42 (5.1)	102 (12.3)	205 (24.8)	29 (3.5)	74 (8.9)	72 (8.7)	37 (4.5)
Arteriolar vasodilators		0 (0)	5 (0.6)	40 (4.8)	0 (0)	3 (0.4)	3 (0.4)	12 (1.5)
Autonomic ganglionic vasodilators		0 (0)	0 (0)	0 (0)	0 (0)	0 (0)	0 (0)	0 (0)
Beta adrenergic blockers (**β-blockers**)		16 (1.9)	55 (6.7)	184 (22.2)	18 (2.2)	38 (4.6)	58 (7)	35 (4.2)
Calcium channel blocker (**CCB**)-dihydropyridine/non-dihydropyridine	37 (4.5)	87 (10.5)	139 (16.8)	229 (27.7)	0 (0)	0 (0)	0 (0)	0 (0)
Central alpha 2 adrenergic agonists		4 (0.5)	7 (0.8)	61 (7.4)	1 (0.1)	5 (0.6)	13 (1.6)	14 (1.7)
Diuretics: thiazide/thiazide-type		18 (2.2)	64 (7.7)	149 (18)	8 (1)	63 (7.6)	49 (5.9)	28 (3.4)
Diuretics: loop		6 (0.7)	36 (4.4)	114 (13.8)	10 (1.2)	20 (2.4)	38 (4.6)	30 (3.6)
Diuretics: potassium-sparing†		0 (0)	6 (0.7)	21 (2.5)	0 (0)	3 (0.4)	5 (0.6)	5 (0.6)
Peripheral adrenergic neuron antagonist†		0 (0)	0 (0)	0 (0)	0 (0)	0 (0)	0 (0)	0 (0)
Renin inhibitors		0 (0)	0 (0)	0 (0)	0 (0)	0 (0)	1 (0.1)	1 (0.1)
**Total participants**	37 (4.5)	87 (10.5)	139 (16.8)	229 (27.7)	67 (8.1)	106 (12.8)	83 (10)	42 (5.1)

**Angiotensin-converting enzyme (ACE) inhibitor/ Angiotensin II receptor blockers (ARB)** [Randomized to Lisinopril in trial: 802 (26.5)]	1) any ACE/ARB (1 drug)	2) any ACE/ARB plus 1 drug (2 drugs)	3) any ACE/ARB plus 2 drugs (3 drugs)	4) any ACE/ARB plus ≥3 drugs (4+ drugs)	1) no ACE/ARB (on 1 drug)	2) no ACE/ARB (on 2 drugs)	3) no ACE/ARB (on 3 drugs)	4) no ACE/ARB (on 4+ drugs)
Aldosterone antagonists †		0 (0)	2 (0.2)	20 (2.5)	0 (0)	2 (0.2)	1 (0.1)	1 (0.1)
Alpha 1 adrenergic receptor agonist (selective; **α-blocker**)		2 (0.2)	8 (1)	26 (3.2)	1 (0.1)	4 (0.5)	1 (0.1)	5 (0.6)
Angiotensin-converting enzyme (**ACE**) inhibitor/ Angiotensin II receptor blockers (**ARB)**	32 (4)	121 (15.1)	199 (24.8)	234 (29.2)	0 (0)	0 (0)	0 (0)	0 (0)

Arteriolar vasodilators		0 (0)	12 (1.5)	50 (6.2)	0 (0)	6 (0.7)	7 (0.9)	15 (1.9)
Autonomic ganglionic vasodilators		0 (0)	0 (0)	0 (0)	0 (0)	0 (0)	0 (0)	0 (0)
Beta adrenergic blockers (**β-blockers**)		17 (2.1)	95 (11.8)	186 (23.2)	21 (2.6)	39 (4.9)	40 (5)	17 (2.1)
Calcium channel blocker (**CCB**)-dihydropyridine/non-dihydropyridine		32 (4)	92 (11.5)	191 (23.8)	19 (2.4)	23 (2.9)	35 (4.4)	15 (1.9)
Central alpha 2 adrenergic agonists		2 (0.2)	9 (1.1)	70 (8.7)	1 (0.1)	6 (0.7)	5 (0.6)	12 (1.5)
Diuretics: thiazide/thiazide-type		55 (6.9)	117 (14.6)	148 (18.5)	4 (0.5)	20 (2.5)	22 (2.7)	11 (1.4)
Diuretics: loop		13 (1.6)	55 (6.9)	129 (16.1)	7 (0.9)	19 (2.4)	24 (3)	16 (2)
Diuretics: potassium-sparing[†]		0 (0)	8 (1)	23 (2.9)	0 (0)	1 (0.1)	6 (0.7)	4 (0.5)
Peripheral adrenergic neuron antagonist[†]		0 (0)	0 (0)	0 (0)	0 (0)	0 (0)	0 (0)	0 (0)
Renin inhibitors		0 (0)	0 (0)	0 (0)	0 (0)	0 (0)	0 (0)	0 (0)
**Total participants**	32 (4)	121 (15.1)	199 (24.8)	234 (29.2)	53 (6.6)	60 (7.5)	47 (5.9)	22 (2.7)

[†]Not used alone/used in combination for chronic hypertension treatment.

Table S4b. Patterns of antihypertensive drug utilization from Medicare Part-D drug coverage in 2007, by 3 arms and added-on drugs (Non-black)

**Total Non-black Participants: N=4986**	**N (%) of antihypertensive drug who received the study class drug**				**N (%) of antihypertensive drug who did not receive the study trial drug**			
**Diuretic= Thiazide/thiazide-type**<br>[Randomized to Chlorthalidone in trial: 2243 (45)]	1) any diuretic<br>(1 drug)	2) any diuretic plus 1 drug<br>(2 drugs)	3) any diuretic plus 2 drugs<br>(3 drugs)	4) any diuretic plus ≥3 drugs<br>(4+ drugs)	1) no diuretic<br>(on 1 drug)	2) no diuretic<br>(on 2 drugs)	3) no diuretic<br>(on 3 drugs)	4) no diuretic<br>(on 4+ drugs)
Aldosterone antagonists †		1 (0)	6 (0.3)	35 (1.6)	1 (0)	12 (0.5)	19 (0.8)	50 (2.2)
Alpha 1 adrenergic receptor agonist (selective; **α-blocker**)		2 (0.1)	15 (0.7)	49 (2.2)	6 (0.3)	16 (0.7)	26 (1.2)	34 (1.5)
Angiotensin-converting enzyme (**ACE**) inhibitor/ Angiotensin II receptor blockers (**ARB)**		126 (5.6)	261 (11.6)	312 (13.9)	112 (5)	259 (11.5)	244 (10.9)	198 (8.8)
Arteriolar vasodilators		2 (0.1)	7 (0.3)	29 (1.3)	0 (0)	2 (0.1)	11 (0.5)	41 (1.8)
Autonomic ganglionic vasodilators		0 (0)	0 (0)	0 (0)	0 (0)	0 (0)	0 (0)	0 (0)
Beta adrenergic blockers (**β-blockers**)		48 (2.1)	189 (8.4)	284 (12.7)	65 (2.9)	201 (9)	241 (10.7)	198 (8.8)
Calcium channel blocker (**CCB**)-dihydropyridine/non-dihydropyridine		17 (0.8)	110 (4.9)	257 (11.5)	50 (2.2)	139 (6.2)	146 (6.5)	158 (7)
Central alpha 2 adrenergic agonists		4 (0.2)	11 (0.5)	61 (2.7)	3 (0.1)	18 (0.8)	15 (0.7)	64 (2.9)
Diuretics: thiazide/thiazide-type	50 (2.2)	218 (9.7)	327 (14.6)	349 (15.6)	0 (0)	0 (0)	0 (0)	0 (0)
Diuretics: loop		4 (0.2)	23 (1)	136 (6.1)	25 (1.1)	116 (5.2)	172 (7.7)	193 (8.6)
Diuretics: potassium-sparing†		14 (0.6)	31 (1.4)	49 (2.2)	2 (0.1)	3 (0.1)	2 (0.1)	2 (0.1)
Peripheral adrenergic neuron antagonist†		0 (0)	1 (0)	2 (0.1)	0 (0)	0 (0)	0 (0)	1 (0)
Renin inhibitors		0 (0)	0 (0)	3 (0.1)	0 (0)	0 (0)	0 (0)	0 (0)
**Total participants**	50 (2.2)	218 (9.7)	327 (14.6)	349 (15.6)	264 (11.8)	383 (17.1)	292 (13)	218 (9.7)
**Calcium channel blocker (CCB)**<br>[Randomized to Amlodipine in trial: 1363 (27.3)]	1) any CCB<br>(1 drug)	2) any CCB plus 1 drug	3) any CCB plus 2 drugs	4) any CCB plus ≥3 drugs	1) no CCB (on 1 drug)	2) no CCB (on 2 drugs)	3) no CCB (on 3 drugs)	4) no CCB (on 4+ drugs)

		(2 drugs)	(3 drugs)	(4+ drugs)				
Aldosterone antagonists †		2 (0.1)	4 (0.3)	17 (1.2)	1 (0.1)	4 (0.3)	11 (0.8)	16 (1.2)
Alpha 1 adrenergic receptor agonist (selective; **α-blocker**)		3 (0.2)	5 (0.4)	33 (2.4)	4 (0.3)	6 (0.4)	16 (1.2)	16 (1.2)
Angiotensin-converting enzyme (**ACE**) inhibitor/Angiotensin II receptor blockers (**ARB)**		72 (5.3)	135 (9.9)	223 (16.4)	70 (5.1)	185 (13.6)	148 (10.9)	63 (4.6)
Arteriolar vasodilators		0 (0)	5 (0.4)	28 (2.1)	0 (0)	3 (0.2)	4 (0.3)	9 (0.7)
Autonomic ganglionic vasodilators		0 (0)	0 (0)	0 (0)	0 (0)	0 (0)	0 (0)	0 (0)
Beta adrenergic blockers (**β-blockers**)		36 (2.6)	86 (6.3)	202 (14.8)	53 (3.9)	107 (7.9)	143 (10.5)	68 (5)
Calcium channel blocker (**CCB**)-dihydropyridine/non-dihydropyridine	56 (4.1)	136 (10)	183 (13.4)	242 (17.8)	0 (0)	0 (0)	0 (0)	0 (0)
Central alpha 2 adrenergic agonists		1 (0.1)	10 (0.7)	44 (3.2)	0 (0)	3 (0.2)	11 (0.8)	11 (0.8)
Diuretics: thiazide/thiazide-type		10 (0.7)	71 (5.2)	152 (11.2)	18 (1.3)	107 (7.9)	105 (7.7)	48 (3.5)
Diuretics: loop		11 (0.8)	44 (3.2)	136 (10)	13 (1)	66 (4.8)	75 (5.5)	53 (3.9)
Diuretics: potassium-sparing†		1 (0.1)	6 (0.4)	19 (1.4)	0 (0)	6 (0.4)	11 (0.8)	7 (0.5)
Peripheral adrenergic neuron antagonist†		0 (0)	0 (0)	0 (0)	0 (0)	1 (0.1)	1 (0.1)	0 (0)
Renin inhibitors		0 (0)	0 (0)	2 (0.1)	0 (0)	0 (0)	0 (0)	0 (0)
**Total participants**	56 (4.1)	136 (10)	183 (13.4)	242 (17.8)	159 (11.7)	244 (17.9)	175 (12.8)	70 (5.1)

**Angiotensin-converting enzyme (ACE) inhibitor/ Angiotensin II receptor blockers (ARB)** [Randomized to Lisinopril in trial: 1380 (27.7)]	1) any ACE/ARB (1 drug)	2) any ACE/ARB plus 1 drug (2 drugs)	3) any ACE/ARB plus 2 drugs (3 drugs)	4) any ACE/ARB plus ≥3 drugs (4+ drugs)	1) no ACE/ARB (on 1 drug)	2) no ACE/ARB (on 2 drugs)	3) no ACE/ARB (on 3 drugs)	4) no ACE/ARB (on 4+ drugs)
Aldosterone antagonists †		0 (0)	5 (0.4)	43 (3.1)	3 (0.2)	8 (0.6)	11 (0.8)	5 (0.4)
Alpha 1 adrenergic receptor agonist (selective; **α-blocker**)		4 (0.3)	16 (1.2)	42 (3)	4 (0.3)	8 (0.6)	6 (0.4)	1 (0.1)
Angiotensin-converting enzyme (**ACE**) inhibitor/ Angiotensin II receptor blockers (**ARB)**	94 (6.8)	261 (18.9)	327 (23.7)	294 (21.3)	0 (0)	0 (0)	0 (0)	0 (0)

Arteriolar vasodilators		1 (0.1)	7 (0.5)	38 (2.8)	1 (0.1)	7 (0.5)	5 (0.4)	4 (0.3)
Autonomic ganglionic vasodilators		0 (0)	0 (0)	0 (0)	0 (0)	0 (0)	0 (0)	0 (0)
Beta adrenergic blockers (**β-blockers**)		75 (5.4)	197 (14.3)	248 (18)	52 (3.8)	66 (4.8)	51 (3.7)	18 (1.3)
Calcium channel blocker (**CCB**)-dihydropyridine/non-dihydropyridine		66 (4.8)	139 (10.1)	217 (15.7)	33 (2.4)	47 (3.4)	44 (3.2)	15 (1.1)
Central alpha 2 adrenergic agonists		4 (0.3)	20 (1.4)	65 (4.7)	3 (0.2)	9 (0.7)	8 (0.6)	5 (0.4)
Diuretics: thiazide/thiazide-type		78 (5.7)	145 (10.5)	186 (13.5)	14 (1)	27 (2)	36 (2.6)	14 (1)
Diuretics: loop		33 (2.4)	117 (8.5)	174 (12.6)	15 (1.1)	33 (2.4)	37 (2.7)	10 (0.7)
Diuretics: potassium-sparing[†]		0 (0)	8 (0.6)	23 (1.7)	1 (0.1)	1 (0.1)	11 (0.8)	6 (0.4)
Peripheral adrenergic neuron antagonist[†]		0 (0)	0 (0)	3 (0.2)	0 (0)	0 (0)	0 (0)	0 (0)
Renin inhibitors		0 (0)	0 (0)	2 (0.1)	1 (0.1)	0 (0)	1 (0.1)	0 (0)
**Total participants**	94 (6.8)	261 (18.9)	327 (23.7)	294 (21.3)	127 (9.2)	103 (7.5)	70 (5.1)	19 (1.4)

[†]Not used alone/used in combination for chronic hypertension treatment.

Table S4c. Patterns of antihypertensive drug utilization from Medicare Part-D drug coverage in 2007, by 3 arms and added-on drugs (Male)

**Total Male Participants: N=2812**	**N (%) of antihypertensive drug who received the study class drug**				**N (%) of antihypertensive drug who did not receive the study trial drug**			
**Diuretic= Thiazide/thiazide-type**<br>[Randomized to Chlorthalidone in trial: 1283 (45.6)]	1) any diuretic<br>(1 drug)	2) any diuretic plus 1 drug<br>(2 drugs)	3) any diuretic plus 2 drugs<br>(3 drugs)	4) any diuretic plus ≥3 drugs<br>(4+ drugs)	1) no diuretic<br>(on 1 drug)	2) no diuretic<br>(on 2 drugs)	3) no diuretic<br>(on 3 drugs)	4) no diuretic<br>(on 4+ drugs)
Aldosterone antagonists †		1 (0.1)	2 (0.2)	17 (1.3)	0 (0)	3 (0.2)	10 (0.8)	32 (2.5)
Alpha 1 adrenergic receptor agonist (selective; **α-blocker**)		2 (0.2)	17 (1.3)	46 (3.6)	6 (0.5)	14 (1.1)	30 (2.3)	36 (2.8)
Angiotensin-converting enzyme (**ACE**) inhibitor/ Angiotensin II receptor blockers (**ARB)**		57 (4.4)	143 (11.1)	180 (14)	65 (5.1)	142 (11.1)	135 (10.5)	119 (9.3)
Arteriolar vasodilators		0 (0)	3 (0.2)	23 (1.8)	0 (0)	1 (0.1)	11 (0.9)	33 (2.6)
Autonomic ganglionic vasodilators		0 (0)	0 (0)	0 (0)	0 (0)	0 (0)	0 (0)	0 (0)
Beta adrenergic blockers (**β-blockers**)		25 (1.9)	93 (7.2)	167 (13)	49 (3.8)	107 (8.3)	131 (10.2)	119 (9.3)
Calcium channel blocker (**CCB**)-dihydropyridine/non-dihydropyridine		10 (0.8)	62 (4.8)	139 (10.8)	27 (2.1)	68 (5.3)	81 (6.3)	96 (7.5)
Central alpha 2 adrenergic agonists		0 (0)	7 (0.5)	38 (3)	4 (0.3)	6 (0.5)	9 (0.7)	41 (3.2)
Diuretics: thiazide/thiazide-type	32 (2.5)	104 (8.1)	176 (13.7)	202 (15.7)	0 (0)	0 (0)	0 (0)	0 (0)
Diuretics: loop		1 (0.1)	12 (0.9)	77 (6)	11 (0.9)	57 (4.4)	88 (6.9)	114 (8.9)
Diuretics: potassium-sparing†		8 (0.6)	13 (1)	31 (2.4)	0 (0)	2 (0.2)	3 (0.2)	4 (0.3)
Peripheral adrenergic neuron antagonist†		0 (0)	0 (0)	1 (0.1)	0 (0)	0 (0)	0 (0)	0 (0)
Renin inhibitors		0 (0)	0 (0)	1 (0.1)	0 (0)	0 (0)	0 (0)	0 (0)
**Total participants**	32 (2.5)	104 (8.1)	176 (13.7)	202 (15.7)	162 (12.6)	200 (15.6)	166 (12.9)	134 (10.4)
**Calcium channel blocker (CCB)**<br>[Randomized to Amlodipine in trial: 747 (26.6)]	1) any CCB<br>(1 drug)	2) any CCB plus 1 drug<br>(2 drugs)	3) any CCB plus 2 drugs<br>(3 drugs)	4) any CCB plus ≥3 drugs<br>(4+ drugs)	1) no CCB<br>(on 1 drug)	2) no CCB<br>(on 2 drugs)	3) no CCB<br>(on 3 drugs)	4) no CCB<br>(on 4+ drugs)

Aldosterone antagonists †		0 (0)	1 (0.1)	11 (1.5)	1 (0.1)	2 (0.3)	4 (0.5)	8 (1.1)
Alpha 1 adrenergic receptor agonist (selective; **α-blocker**)		4 (0.5)	6 (0.8)	29 (3.9)	4 (0.5)	9 (1.2)	20 (2.7)	19 (2.5)
Angiotensin-converting enzyme (**ACE**) inhibitor/Angiotensin II receptor blockers (**ARB**)		47 (6.3)	71 (9.5)	125 (16.7)	32 (4.3)	73 (9.8)	74 (9.9)	35 (4.7)
Arteriolar vasodilators		0 (0)	4 (0.5)	20 (2.7)	0 (0)	2 (0.3)	1 (0.1)	5 (0.7)
Autonomic ganglionic vasodilators		0 (0)	0 (0)	0 (0)	0 (0)	0 (0)	0 (0)	0 (0)
Beta adrenergic blockers (**β-blockers**)		18 (2.4)	45 (6)	121 (16.2)	29 (3.9)	52 (7)	60 (8)	34 (4.6)
Calcium channel blocker (**CCB**)-dihydropyridine/non-dihydropyridine	29 (3.9)	83 (11.1)	100 (13.4)	144 (19.3)	0 (0)	0 (0)	0 (0)	0 (0)
Central alpha 2 adrenergic agonists		1 (0.1)	7 (0.9)	32 (4.3)	0 (0)	4 (0.5)	8 (1.1)	5 (0.7)
Diuretics: thiazide/thiazide-type		9 (1.2)	35 (4.7)	93 (12.4)	9 (1.2)	54 (7.2)	51 (6.8)	21 (2.8)
Diuretics: loop		3 (0.4)	26 (3.5)	74 (9.9)	9 (1.2)	18 (2.4)	41 (5.5)	30 (4)
Diuretics: potassium-sparing†		1 (0.1)	5 (0.7)	12 (1.6)	0 (0)	5 (0.7)	4 (0.5)	5 (0.7)
Peripheral adrenergic neuron antagonist†		0 (0)	0 (0)	0 (0)	0 (0)	1 (0.1)	1 (0.1)	0 (0)
Renin inhibitors		0 (0)	0 (0)	1 (0.1)	0 (0)	0 (0)	0 (0)	0 (0)
**Total participants**	29 (3.9)	83 (11.1)	100 (13.4)	144 (19.3)	84 (11.2)	110 (14.7)	88 (11.8)	37 (5)
**Angiotensin-converting enzyme (ACE) inhibitor/ Angiotensin II receptor blockers (ARB)** [Randomized to Lisinopril in trial: 782 (27.8)]	1) any ACE/ARB (1 drug)	2) any ACE/ARB plus 1 drug (2 drugs)	3) any ACE/ARB plus 2 drugs (3 drugs)	4) any ACE/ARB plus ≥3 drugs (4+ drugs)	1) no ACE/ARB (on 1 drug)	2) no ACE/ARB (on 2 drugs)	3) no ACE/ARB (on 3 drugs)	4) no ACE/ARB (on 4+ drugs)
Aldosterone antagonists †		0 (0)	3 (0.4)	31 (4)	2 (0.3)	6 (0.8)	8 (1)	3 (0.4)
Alpha 1 adrenergic receptor agonist (selective; **α-blocker**)		6 (0.8)	22 (2.8)	44 (5.6)	5 (0.6)	12 (1.5)	5 (0.6)	4 (0.5)
Angiotensin-converting enzyme (**ACE**) inhibitor/ Angiotensin II receptor blockers (**ARB**)	49 (6.3)	130 (16.6)	167 (21.4)	176 (22.5)	0 (0)	0 (0)	0 (0)	0 (0)

Arteriolar vasodilators		0 (0)	7 (0.9)	33 (4.2)	0 (0)	8 (1)	6 (0.8)	8 (1)
Autonomic ganglionic vasodilators		0 (0)	0 (0)	0 (0)	0 (0)	0 (0)	0 (0)	0 (0)
Beta adrenergic blockers (**β-blockers**)		42 (5.4)	107 (13.7)	148 (18.9)	38 (4.9)	37 (4.7)	25 (3.2)	15 (1.9)
Calcium channel blocker (**CCB**)-dihydropyridine/non-dihydropyridine		38 (4.9)	66 (8.4)	128 (16.4)	16 (2)	20 (2.6)	24 (3.1)	9 (1.2)
Central alpha 2 adrenergic agonists		2 (0.3)	9 (1.2)	38 (4.9)	2 (0.3)	5 (0.6)	3 (0.4)	7 (0.9)
Diuretics: thiazide/thiazide-type		32 (4.1)	61 (7.8)	101 (12.9)	7 (0.9)	20 (2.6)	18 (2.3)	9 (1.2)
Diuretics: loop		10 (1.3)	56 (7.2)	105 (13.4)	9 (1.2)	20 (2.6)	21 (2.7)	7 (0.9)
Diuretics: potassium-sparing†		0 (0)	3 (0.4)	16 (2)	0 (0)	0 (0)	4 (0.5)	6 (0.8)
Peripheral adrenergic neuron antagonist†		0 (0)	0 (0)	3 (0.4)	0 (0)	0 (0)	0 (0)	0 (0)
Renin inhibitors		0 (0)	0 (0)	1 (0.1)	1 (0.1)	0 (0)	0 (0)	0 (0)
**Total participants**	49 (6.3)	130 (16.6)	167 (21.4)	176 (22.5)	80 (10.2)	64 (8.2)	38 (4.9)	16 (2)

†Not used alone/used in combination for chronic hypertension treatment.

Table S4d. Patterns of antihypertensive drug utilization from Medicare Part-D drug coverage in 2007, by 3 arms and added-on drugs (Female)

**Total Female Participants: N=5198**	**N (%) of antihypertensive drug who received the study class drug**				**N (%) of antihypertensive drug who did not receive the study trial drug**			
**Diuretic= Thiazide/thiazide-type**<br>[Randomized to Chlorthalidone in trial: 2355 (45.3)]	1) any diuretic<br>(1 drug)	2) any diuretic plus 1 drug<br>(2 drugs)	3) any diuretic plus 2 drugs<br>(3 drugs)	4) any diuretic plus ≥3 drugs<br>(4+ drugs)	1) no diuretic<br>(on 1 drug)	2) no diuretic<br>(on 2 drugs)	3) no diuretic<br>(on 3 drugs)	4) no diuretic<br>(on 4+ drugs)
Aldosterone antagonists †		0 (0)	5 (0.2)	34 (1.4)	3 (0.1)	10 (0.4)	20 (0.8)	50 (2.1)
Alpha 1 adrenergic receptor agonist (selective; **α-blocker**)		0 (0)	4 (0.2)	27 (1.1)	1 (0)	3 (0.1)	4 (0.2)	11 (0.5)
Angiotensin-converting enzyme (**ACE**) inhibitor/ Angiotensin II receptor blockers (**ARB)**		145 (6.2)	289 (12.3)	391 (16.6)	97 (4.1)	225 (9.6)	236 (10)	219 (9.3)
Arteriolar vasodilators		2 (0.1)	8 (0.3)	36 (1.5)	1 (0)	3 (0.1)	10 (0.4)	46 (2)
Autonomic ganglionic vasodilators		0 (0)	0 (0)	0 (0)	0 (0)	0 (0)	0 (0)	0 (0)
Beta adrenergic blockers (**β-blockers**)		57 (2.4)	181 (7.7)	341 (14.5)	40 (1.7)	172 (7.3)	222 (9.4)	211 (9)
Calcium channel blocker (**CCB**)-dihydropyridine/non-dihydropyridine		24 (1)	158 (6.7)	336 (14.3)	79 (3.4)	163 (6.9)	171 (7.3)	188 (8)
Central alpha 2 adrenergic agonists		6 (0.3)	10 (0.4)	83 (3.5)	4 (0.2)	21 (0.9)	30 (1.3)	71 (3)
Diuretics: thiazide/thiazide-type	50 (2.1)	262 (11.1)	363 (15.4)	424 (18)	0 (0)	0 (0)	0 (0)	0 (0)
Diuretics: loop		8 (0.3)	32 (1.4)	155 (6.6)	26 (1.1)	123 (5.2)	185 (7.9)	207 (8.8)
Diuretics: potassium-sparing†		20 (0.8)	38 (1.6)	60 (2.5)	3 (0.1)	4 (0.2)	1 (0)	2 (0.1)
Peripheral adrenergic neuron antagonist†		0 (0)	1 (0)	2 (0.1)	0 (0)	0 (0)	0 (0)	2 (0.1)
Renin inhibitors		0 (0)	0 (0)	10 (0.4)	0 (0)	0 (0)	0 (0)	0 (0)
**Total participants**	50 (2.1)	262 (11.1)	363 (15.4)	424 (18)	254 (10.8)	362 (15.4)	293 (12.4)	237 (10.1)
**Calcium channel blocker (CCB)**<br>[Randomized to Amlodipine in trial: 1443 (27.8)]	1) any CCB<br>(1 drug)	2) any CCB plus 1 drug<br>(2 drugs)	3) any CCB plus 2 drugs<br>(3 drugs)	4) any CCB plus ≥3 drugs<br>(4+ drugs)	1) no CCB<br>(on 1 drug)	2) no CCB<br>(on 2 drugs)	3) no CCB<br>(on 3 drugs)	4) no CCB<br>(on 4+ drugs)

Aldosterone antagonists †		2 (0.1)	4 (0.3)	21 (1.5)	0 (0)	4 (0.3)	12 (0.8)	17 (1.2)
Alpha 1 adrenergic receptor agonist (selective; **α-blocker**)		0 (0)	1 (0.1)	24 (1.7)	1 (0.1)	1 (0.1)	1 (0.1)	5 (0.3)
Angiotensin-converting enzyme (**ACE**) inhibitor/Angiotensin II receptor blockers (**ARB)**		67 (4.6)	166 (11.5)	303 (21)	67 (4.6)	186 (12.9)	146 (10.1)	65 (4.5)
Arteriolar vasodilators		0 (0)	6 (0.4)	48 (3.3)	0 (0)	4 (0.3)	6 (0.4)	16 (1.1)
Autonomic ganglionic vasodilators		0 (0)	0 (0)	0 (0)	0 (0)	0 (0)	0 (0)	0 (0)
Beta adrenergic blockers (**β-blockers**)		34 (2.4)	96 (6.7)	265 (18.4)	42 (2.9)	93 (6.4)	141 (9.8)	69 (4.8)
Calcium channel blocker (**CCB**)-dihydropyridine/non-dihydropyridine	64 (4.4)	140 (9.7)	222 (15.4)	327 (22.7)	0 (0)	0 (0)	0 (0)	0 (0)
Central alpha 2 adrenergic agonists		4 (0.3)	10 (0.7)	73 (5.1)	1 (0.1)	4 (0.3)	16 (1.1)	20 (1.4)
Diuretics: thiazide/thiazide-type		19 (1.3)	100 (6.9)	208 (14.4)	17 (1.2)	116 (8)	103 (7.1)	55 (3.8)
Diuretics: loop		14 (1)	54 (3.7)	176 (12.2)	14 (1)	68 (4.7)	72 (5)	53 (3.7)
Diuretics: potassium-sparing†		0 (0)	7 (0.5)	28 (1.9)	0 (0)	4 (0.3)	12 (0.8)	7 (0.5)
Peripheral adrenergic neuron antagonist†		0 (0)	0 (0)	0 (0)	0 (0)	0 (0)	0 (0)	0 (0)
Renin inhibitors		0 (0)	0 (0)	1 (0.1)	0 (0)	0 (0)	1 (0.1)	1 (0.1)
**Total participants**	64 (4.4)	140 (9.7)	222 (15.4)	327 (22.7)	142 (9.8)	240 (16.6)	170 (11.8)	75 (5.2)

**Angiotensin-converting enzyme (ACE) inhibitor/ Angiotensin II receptor blockers (ARB)** [Randomized to Lisinopril in trial: 1400 (26.9)]	1) any ACE/ARB (1 drug)	2) any ACE/ARB plus 1 drug (2 drugs)	3) any ACE/ARB plus 2 drugs (3 drugs)	4) any ACE/ARB plus ≥3 drugs (4+ drugs)	1) no ACE/ARB (on 1 drug)	2) no ACE/ARB (on 2 drugs)	3) no ACE/ARB (on 3 drugs)	4) no ACE/ARB (on 4+ drugs)
Aldosterone antagonists †		0 (0)	4 (0.3)	32 (2.3)	1 (0.1)	4 (0.3)	4 (0.3)	3 (0.2)
Alpha 1 adrenergic receptor agonist (selective; **α-blocker**)		0 (0)	2 (0.1)	24 (1.7)	0 (0)	0 (0)	2 (0.1)	2 (0.1)
Angiotensin-converting enzyme (**ACE**) inhibitor/ Angiotensin II receptor blockers (**ARB)**	77 (5.5)	252 (18)	359 (25.6)	352 (25.1)	0 (0)	0 (0)	0 (0)	0 (0)

Arteriolar vasodilators		1 (0.1)	12 (0.9)	55 (3.9)	1 (0.1)	5 (0.4)	6 (0.4)	11 (0.8)
Autonomic ganglionic vasodilators		0 (0)	0 (0)	0 (0)	0 (0)	0 (0)	0 (0)	0 (0)
Beta adrenergic blockers (**β-blockers**)		50 (3.6)	185 (13.2)	286 (20.4)	35 (2.5)	68 (4.9)	66 (4.7)	20 (1.4)
Calcium channel blocker (**CCB**)-dihydropyridine/non-dihydropyridine		60 (4.3)	165 (11.8)	280 (20)	36 (2.6)	50 (3.6)	55 (3.9)	21 (1.5)
Central alpha 2 adrenergic agonists		4 (0.3)	20 (1.4)	97 (6.9)	2 (0.1)	10 (0.7)	10 (0.7)	10 (0.7)
Diuretics: thiazide/thiazide-type		101 (7.2)	201 (14.4)	233 (16.6)	11 (0.8)	27 (1.9)	40 (2.9)	16 (1.1)
Diuretics: loop		36 (2.6)	116 (8.3)	198 (14.1)	13 (0.9)	32 (2.3)	40 (2.9)	19 (1.4)
Diuretics: potassium-sparing†		0 (0)	13 (0.9)	30 (2.1)	1 (0.1)	2 (0.1)	13 (0.9)	4 (0.3)
Peripheral adrenergic neuron antagonist†		0 (0)	0 (0)	0 (0)	0 (0)	0 (0)	0 (0)	0 (0)
Renin inhibitors		0 (0)	0 (0)	1 (0.1)	0 (0)	0 (0)	1 (0.1)	0 (0)
**Total participants**	77 (5.5)	252 (18)	359 (25.6)	352 (25.1)	100 (7.1)	99 (7.1)	79 (5.6)	25 (1.8)

†Not used alone/used in combination for chronic hypertension treatment.

Table S5. Cumulative Incidence for morbidity/mortality outcomes between 2007 and 2017 by the changing patterns of antihypertensive drugs as of 2007

**Total Participants: N=8007**	**10-year rate per 100 persons in those who received the study trial class drug as of 2007**				**10-year rate per 100 persons in those who did not receive the study trial drug as of 2007**				
**Diuretic= Thiazide/thiazide-type** [Randomized to Chlorthalidone in trial: 3637 (45.4)]	1) any diuretic (1 drug)	2) any diuretic plus 1 drug (2 drugs)	3) any diuretic plus 2 drugs (3 drugs)	4) any diuretic plus ≥3 drugs (4+ drugs)	1) no diuretic (on 1 drug)	2) no diuretic (on 2 drugs)	3) no diuretic (on 3 drugs)	4) no diuretic (on 4+ drugs)	5) No Drugs
**Mortality Outcomes:**									
**All-cause mortality**	24.4	22.4	22.4	30.2	25.3	29.5	35.5	48.2	23.5
**CVD mortality**	11.8	10.3	10.1	13.3	11.3	13.6	17.5	27.3	9.5
CHD mortality	6.8	4.5	3.8	6.0	3.8	5.4	9.7	14.3	4.7
Stroke mortality	1.3	1.9	2.0	2.0	2.2	2.1	2.6	4.1	2.1
Heart failure mortality	1.2	0.9	1.2	1.9	1.1	1.7	1.7	2.9	0.5
Other CVD mortality	2.9	3.5	3.3	3.9	4.7	5.2	4.5	8.9	2.6
**Non-CVD Mortality**	13.1	13.4	13.6	19.5	15.3	18.2	21.8	28.8	15.4
Cancer	4.2	6.2	4.2	6.0	3.8	4.4	4.7	3.7	4.1
Kidney disease	1.4	0.6	0.4	1.7	0.8	2.0	2.6	3.9	0.5
Accident/suicide/homicide	0.0	0.0	0.6	0.9	0.6	0.2	0.6	1.6	0.5
Other non-CVD disease	8.0	7.1	8.8	12.0	10.7	12.5	15.4	21.8	10.9
**Combined fatal and non-fatal Hospitalized events:**									
CVD	29.7	24.7	29.4	38.4	29.1	32.3	42.0	55.3	24.4
CHD	12.0	7.4	9.8	12.1	10.3	10.9	16.5	23.0	8.1
Heart failure	18.8	18.0	24.1	34.2	23.4	27.0	37.6	51.0	18.8
Stroke	8.0	10.1	9.6	11.3	8.4	10.6	11.3	17.1	7.1
Cancer	13.1	11.0	7.9	12.0	8.4	11.6	12.0	12.3	7.4

Kidney disease/ESRD	2.7	0.9	1.6	5.5	2.4	5.6	6.5	13.0	4.4

**Calcium channel blocker (CCB)** [Randomized to Amlodipine in trial: 2189 (27.3)]	1) any CCB (1 drug)	2) any CCB plus 1 drug (2 drugs)	3) any CCB plus 2 drugs (3 drugs)	4) any CCB plus ≥3 drugs (4+ drugs)	1) no CCB (on 1 drug)	2) no CCB (on 2 drugs)	3) no CCB (on 3 drugs)	4) no CCB (on 4+ drugs)	5) No Drugs
**Mortality Outcomes:**									
**All-cause mortality**	24.7	21.1	29.5	35.0	23.9	24.0	33.7	39.3	23.9
**CVD mortality**	12.5	6.3	15.0	15.3	9.3	10.2	17.7	23.9	11.2
CHD mortality	4.6	2.5	4.8	7.4	3.9	4.7	7.1	11.8	5.0
Stroke mortality	0.0	0.5	2.5	2.1	1.6	1.6	2.2	2.5	2.5
Heart failure mortality	0.0	0.5	3.3	2.2	0.4	0.6	2.2	7.4	1.8
Other CVD mortality	8.3	2.9	5.2	4.5	3.6	3.5	7.3	4.5	2.4
**Non-CVD Mortality**	13.9	15.7	17.1	23.3	16.1	15.1	19.5	20.2	14.3
Cancer	4.9	5.7	5.3	5.1	5.3	4.8	5.8	5.6	5.1
Kidney disease	1.2	1.5	1.0	1.8	0.5	0.6	2.9	2.2	0.9
Accident/suicide/homicide	0.0	0.0	0.0	0.3	1.4	0.3	0.6	0.0	0.0
Other non-CVD disease	8.4	9.3	11.6	17.5	9.6	10.0	11.5	13.6	8.8
**Combined fatal and non-fatal**									
**Hospitalized events:**									
CVD	25.1	26.0	36.0	41.6	26.5	28.5	39.0	44.0	24.3
CHD	9.3	9.2	14.5	14.9	7.2	9.7	13.3	15.4	9.5
Heart failure	21.2	19.7	30.5	36.3	20.0	24.3	34.9	43.1	19.2
Stroke	5.6	8.9	12.0	12.5	9.5	11.5	9.0	13.0	7.2
Cancer	9.3	9.6	9.6	11.8	11.4	10.9	9.4	13.8	9.8
Kidney disease/ESRD	4.7	2.5	5.4	8.9	4.0	2.9	4.3	6.8	4.0

**Angiotensin-converting enzyme (ACE) inhibitor/ Angiotensin II receptor blockers (ARB)**<br>[Randomized to Lisinopril in trial: 2181 (27.2)]	1) any ACE/ARB<br>(1 drug)	2) any ACE/ARB plus 1 drug<br>(2 drugs)	3) any ACE/ARB plus 2 drugs<br>(3 drugs)	4) any ACE/ARB plus ≥3 drugs<br>(4+ drugs)	1) no ACE/ARB<br>(on 1 drug)	2) no ACE/ARB<br>(on 2 drugs)	3) no ACE/ARB<br>(on 3 drugs)	4) no ACE/ARB<br>(on 4+ drugs)	5) No Drugs
**Mortality Outcomes:**									
**All-cause mortality**	21.4	22.0	30.4	35.0	27.4	33.7	32.5	34.1	21.0
**CVD mortality**	13.4	8.6	14.9	18.7	14.3	17.8	14.4	13.9	6.5
CHD mortality	7.1	4.3	6.3	8.9	8.0	5.8	8.7	2.8	2.8
Stroke mortality	1.8	1.2	1.6	2.4	1.8	5.5	1.1	0.0	0.8
Heart failure mortality	1.8	1.2	3.1	2.8	1.2	3.1	1.0	3.3	0.0
Other CVD mortality	3.4	2.1	4.7	5.9	4.0	4.6	4.2	8.4	2.9
**Non-CVD Mortality**	9.3	14.7	18.2	20.1	15.2	19.4	21.2	23.5	15.5
Cancer	4.2	3.5	5.2	5.8	5.0	4.7	4.8	8.2	9.8
Kidney disease	1.0	0.9	0.6	1.8	3.0	2.4	2.0	3.1	0.9
Accident/suicide/homicide	0.0	0.9	0.9	0.5	0.7	2.2	0.0	2.9	0.0
Other non-CVD disease	4.4	10.0	12.4	13.3	7.4	11.4	15.5	11.5	5.6
**Combined fatal and non-fatal**									
**Hospitalized events:**									
CVD	25.7	27.9	36.3	43.0	28.0	37.3	33.3	30.7	17.5
CHD	11.9	9.2	14.2	15.9	9.9	12.7	14.6	8.4	3.7
Heart failure	17.2	22.4	33.2	39.1	20.7	31.9	27.8	28.2	13.5
Stroke	9.3	10.2	11.5	12.3	12.1	14.6	8.5	11.4	9.1
Cancer	9.2	7.6	11.4	12.5	10.2	11.4	9.7	10.4	11.4
Kidney disease/ESRD	1.0	2.8	3.2	7.8	5.8	6.1	6.0	13.2	4.5

Table S6. Morbidity/mortality outcomes between 2007 and 2017 by the pattern of antihypertensive drug use

Total Participants: N=8007	N (%) of having morbidity/mortality outcomes in participants who received the study class drug				N (%) of having morbidity/mortality outcomes in participants who did not receive the study trial drug				
**Diuretic= Thiazide/thiazide-type**<br>[Randomized to Chlorthalidone in trial: 3637 (45.4)]	1) any diuretic<br>(1 drug)	2) any diuretic plus 1 drug<br>(2 drugs)	3) any diuretic plus 2 drugs<br>(3 drugs)	4) any diuretic plus ≥3 drugs<br>(4+ drugs)	1) no diuretic<br>(on 1 drug)	2) no diuretic<br>(on 2 drugs)	3) no diuretic<br>(on 3 drugs)	4) no diuretic<br>(on 4+ drugs)	5) No Drugs
**Mortality Outcomes:**									
**All-cause mortality**	46 (56.1)	214 (58.5)	303 (56.2)	409 (65.3)	285 (68.7)	391 (69.6)	350 (76.3)	308 (83)	157 (72.4)
**CVD mortality**	18 (22)	94 (25.7)	107 (19.9)	170 (27.2)	97 (23.4)	159 (28.3)	160 (34.9)	147 (39.6)	61 (28.1)
CHD mortality	8 (9.8)	36 (9.8)	42 (7.8)	87 (13.9)	41 (9.9)	60 (10.7)	78 (17)	68 (18.3)	26 (12)
Stroke mortality	3 (3.7)	15 (4.1)	18 (3.3)	21 (3.4)	17 (4.1)	24 (4.3)	18 (3.9)	18 (4.9)	11 (5.1)
Heart failure mortality	3 (3.7)	6 (1.6)	10 (1.9)	16 (2.6)	6 (1.4)	13 (2.3)	18 (3.9)	17 (4.6)	5 (2.3)
Other CVD mortality	4 (4.9)	37 (10.1)	37 (6.9)	46 (7.3)	33 (8)	62 (11)	46 (10)	44 (11.9)	19 (8.8)
**Non-CVD Mortality**	27 (32.9)	120 (32.8)	194 (36)	237 (37.9)	186 (44.8)	229 (40.7)	190 (41.4)	161 (43.4)	94 (43.3)
Cancer	6 (7.3)	37 (10.1)	50 (9.3)	54 (8.6)	37 (8.9)	52 (9.3)	43 (9.4)	25 (6.7)	20 (9.2)
Kidney disease	1 (1.2)	6 (1.6)	7 (1.3)	17 (2.7)	7 (1.7)	15 (2.7)	13 (2.8)	17 (4.6)	1 (0.5)
Accident/suicide/homicide	1 (1.2)	3 (0.8)	7 (1.3)	8 (1.3)	10 (2.4)	5 (0.9)	5 (1.1)	8 (2.2)	3 (1.4)
Other non-CVD disease	19 (23.2)	74 (20.2)	130 (24.1)	158 (25.2)	132 (31.8)	157 (27.9)	129 (28.1)	111 (29.9)	70 (32.3)
**Unknown causes of mortality**	1 (1.2)	0 (0)	2 (0.4)	2 (0.3)	2 (0.5)	3 (0.5)	0 (0)	0 (0)	2 (0.9)
**Combined fatal and non-fatal**									

**Hospitalized events:***									
CHD	12 (14.6)	48 (13.1)	72 (13.4)	123 (19.6)	66 (15.9)	88 (15.7)	107 (23.3)	100 (27)	33 (15.2)
CVD	29 (35.4)	113 (30.9)	181 (33.6)	294 (47)	146 (35.2)	214 (38.1)	231 (50.3)	230 (62)	69 (31.8)
Heart failure	18 (22)	67 (18.3)	130 (24.1)	218 (34.8)	98 (23.6)	154 (27.4)	177 (38.6)	196 (52.8)	45 (20.7)
Stroke	8 (9.8)	43 (11.7)	58 (10.8)	75 (12)	41 (9.9)	68 (12.1)	53 (11.5)	59 (15.9)	21 (9.7)
Cancer	14 (17.1)	55 (15)	72 (13.4)	92 (14.7)	55 (13.3)	86 (15.3)	72 (15.7)	52 (14)	27 (12.4)
Kidney disease/ESRD	2 (2.4)	7 (1.9)	14 (2.6)	39 (6.2)	13 (3.1)	34 (6)	28 (6.1)	46 (12.4)	9 (4.1)
**Calcium channel blocker (CCB)**<br>[Randomized to Amlodipine in trial: 2189 (27.3)]	1) any CCB<br>(1 drug)	2) any CCB plus 1 drug<br>(2 drugs)	3) any CCB plus 2 drugs<br>(3 drugs)	4) any CCB plus ≥3 drugs<br>(4+ drugs)	1) no CCB (on 1 drug)	2) no CCB (on 2 drugs)	3) no CCB (on 3 drugs)	4) no CCB (on 4+ drugs)	5) No Drugs
**Mortality Outcomes:**									
**All-cause mortality**	63 (67.7)	144 (64.6)	202 (62.7)	323 (68.6)	158 (69.9)	226 (64.6)	181 (70.2)	89 (79.5)	97 (72.4)
CV mortality	25 (26.9)	46 (20.6)	97 (30.1)	123 (26.1)	62 (27.4)	81 (23.1)	82 (31.8)	41 (36.6)	35 (26.1)
CHD mortality	7 (7.5)	17 (7.6)	42 (13)	56 (11.9)	30 (13.3)	33 (9.4)	31 (12)	18 (16.1)	16 (11.9)
Stroke mortality	1 (1.1)	5 (2.2)	14 (4.3)	15 (3.2)	8 (3.5)	15 (4.3)	11 (4.3)	2 (1.8)	6 (4.5)
Heart failure mortality	1 (1.1)	3 (1.3)	14 (4.3)	19 (4)	6 (2.7)	6 (1.7)	12 (4.7)	12 (10.7)	3 (2.2)
Other CVD mortality	16 (17.2)	21 (9.4)	27 (8.4)	33 (7)	18 (8)	27 (7.7)	28 (10.9)	9 (8)	10 (7.5)
**Non-CV Mortality**	38 (40.9)	98 (43.9)	105 (32.6)	199 (42.3)	96 (42.5)	143 (40.9)	98 (38)	48 (42.9)	62 (46.3)
Cancer	10 (10.8)	26 (11.7)	26 (8.1)	39 (8.3)	19 (8.4)	33 (9.4)	20 (7.8)	12 (10.7)	13 (9.7)
Kidney disease	4 (4.3)	5 (2.2)	5 (1.6)	14 (3)	5 (2.2)	7 (2)	7 (2.7)	4 (3.6)	1 (0.7)
Accident/suicide/homicide	3 (3.2)	3 (1.3)	2 (0.6)	4 (0.8)	4 (1.8)	3 (0.9)	2 (0.8)	0 (0)	1 (0.7)

Other non-CVD disease	21 (22.6)	64 (28.7)	72 (22.4)	142 (30.1)	68 (30.1)	100 (28.6)	69 (26.7)	32 (28.6)	47 (35.1)
**Unknown causes of mortality**	0 (0)	0 (0)	0 (0)	1 (0.2)	0 (0)	2 (0.6)	1 (0.4)	0 (0)	0 (0)
**Combined fatal and non-fatal**									
**Hospitalized events:**									
CHD	11 (11.8)	31 (13.9)	71 (22)	90 (19.1)	37 (16.4)	49 (14)	46 (17.8)	22 (19.6)	23 (17.2)
CVD	26 (28)	69 (30.9)	143 (44.4)	224 (47.6)	82 (36.3)	116 (33.1)	115 (44.6)	55 (49.1)	44 (32.8)
Heart failure	20 (21.5)	44 (19.7)	101 (31.4)	182 (38.6)	49 (21.7)	87 (24.9)	97 (37.6)	53 (47.3)	27 (20.1)
Stroke	7 (7.5)	24 (10.8)	42 (13)	58 (12.3)	25 (11.1)	47 (13.4)	27 (10.5)	12 (10.7)	12 (9)
Cancer	14 (15.1)	34 (15.2)	40 (12.4)	69 (14.6)	32 (14.2)	54 (15.4)	29 (11.2)	21 (18.8)	19 (14.2)
Kidney disease/ESRD	7 (7.5)	7 (3.1)	18 (5.6)	44 (9.3)	12 (5.3)	15 (4.3)	10 (3.9)	8 (7.1)	6 (4.5)

**Angiotensin-converting enzyme (ACE) inhibitor/ Angiotensin II receptor blockers (ARB)** [Randomized to Lisinopril in trial: 2181 (27.2)]	1) any ACE/ARB (1 drug)	2) any ACE/ARB plus 1 drug (2 drugs)	3) any ACE/ARB plus 2 drugs (3 drugs)	4) any ACE/ARB plus ≥3 drugs (4+ drugs)	1) no ACE/ARB (on 1 drug)	2) no ACE/ARB (on 2 drugs)	3) no ACE/ARB (on 3 drugs)	4) no ACE/ARB (on 4+ drugs)	5) No Drugs
**Mortality Outcomes:**									
**All-cause mortality**	82 (65.1)	225 (58.9)	355 (67.5)	370 (70.1)	125 (69.8)	117 (71.8)	85 (72.6)	30 (73.2)	85 (71.4)
CV mortality	27 (21.4)	77 (20.2)	140 (26.6)	189 (35.8)	50 (27.9)	55 (33.7)	33 (28.2)	10 (24.4)	27 (22.7)
CHD mortality	13 (10.3)	29 (7.6)	63 (12)	78 (14.8)	22 (12.3)	23 (14.1)	21 (17.9)	5 (12.2)	9 (7.6)
Stroke mortality	5 (4)	14 (3.7)	15 (2.9)	27 (5.1)	10 (5.6)	12 (7.4)	1 (0.9)	0 (0)	3 (2.5)
Heart failure mortality	2 (1.6)	7 (1.8)	18 (3.4)	25 (4.7)	2 (1.1)	8 (4.9)	1 (0.9)	1 (2.4)	3 (2.5)
Other CVD mortality	7 (5.6)	27 (7.1)	44 (8.4)	59 (11.2)	16 (8.9)	12 (7.4)	10 (8.5)	4 (9.8)	12 (10.1)

**Non-CV Mortality**	55 (43.7)	146 (38.2)	214 (40.7)	178 (33.7)	75 (41.9)	62 (38)	52 (44.4)	20 (48.8)	58 (48.7)
Cancer	14 (11.1)	30 (7.9)	56 (10.6)	36 (6.8)	22 (12.3)	14 (8.6)	11 (9.4)	6 (14.6)	25 (21)
Kidney disease	2 (1.6)	8 (2.1)	12 (2.3)	19 (3.6)	10 (5.6)	7 (4.3)	3 (2.6)	2 (4.9)	4 (3.4)
Accident/suicide/homicide	2 (1.6)	8 (2.1)	7 (1.3)	5 (0.9)	3 (1.7)	4 (2.5)	1 (0.9)	2 (4.9)	0 (0)
Other non-CVD disease	37 (29.4)	100 (26.2)	139 (26.4)	118 (22.3)	40 (22.3)	37 (22.7)	37 (31.6)	10 (24.4)	29 (24.4)
**Unknown causes of mortality**	0 (0)	2 (0.5)	1 (0.2)	3 (0.6)	0 (0)	0 (0)	0 (0)	0 (0)	0 (0)
**Combined fatal and non-fatal Hospitalized events:**									
CHD	21 (16.7)	49 (12.8)	99 (18.8)	114 (21.6)	25 (14)	33 (20.2)	27 (23.1)	7 (17.1)	11 (9.2)
CVD	38 (30.2)	122 (31.9)	223 (42.4)	262 (49.6)	59 (33)	74 (45.4)	50 (42.7)	16 (39)	28 (23.5)
Heart failure	21 (16.7)	89 (23.3)	175 (33.3)	215 (40.7)	36 (20.1)	55 (33.7)	32 (27.4)	11 (26.8)	19 (16)
Stroke	15 (11.9)	46 (12)	64 (12.2)	73 (13.8)	27 (15.1)	25 (15.3)	9 (7.7)	4 (9.8)	13 (10.9)
Cancer	20 (15.9)	45 (11.8)	84 (16)	68 (12.9)	29 (16.2)	24 (14.7)	16 (13.7)	7 (17.1)	27 (22.7)
Kidney disease/ESRD	2 (1.6)	15 (3.9)	24 (4.6)	48 (9.1)	15 (8.4)	13 (8)	7 (6)	6 (14.6)	8 (6.7)

*For morbidity, patients may have more than one category of events.

Table S7a. Adjusted hazard ratio (95% CI) of morbidity and mortality by the end of post-trial (12-31-2017) period by 3 arms

**Total Participants: N=8007**	**In trial participants who received the study trial drug**	**In trial participants who did not receive the study trial drug**
**Diuretic= Thiazide/thiazide-type**		
[Randomized to Chlorthalidone in trial: 3637 (45.4)]	1) any diuretic	1) no diuretic
**Mortality Outcomes:**		
**All-cause mortality**	1.00 (ref)	1.24 (1.13-1.35)
**CVD mortality**	1.00 (ref)	1.31 (1.14-1.51)
CHD mortality	1.00 (ref)	1.30 (1.05-1.60)
Stroke mortality	1.00 (ref)	1.27 (0.87-1.84)
Heart failure mortality	1.00 (ref)	1.11 (0.70-1.76)
Other CVD mortality	1.00 (ref)	1.42 (1.11-1.81)
**Non-CVD Mortality**	1.00 (ref)	1.18 (1.05-1.33)
Cancer	1.00 (ref)	1.04 (0.81-1.33)
Kidney disease	1.00 (ref)	1.34 (0.82-2.20)
Accident/suicide/homicide	1.00 (ref)	1.40 (0.76-2.57)
Other non-CVD disease	1.00 (ref)	1.20 (1.05-1.39)
**Combined fatal and non-fatal**		
**Hospitalized events:**		
CVD	1.00 (ref)	1.23 (1.09-1.37)
CHD	1.00 (ref)	1.25 (1.05-1.49)
Heart failure	1.00 (ref)	1.31 (1.14-1.49)
Stroke	1.00 (ref)	1.20 (0.97-1.49)
Cancer	1.00 (ref)	1.05 (0.87-1.27)

Kidney disease/ESRD	1.00 (ref)	1.57 (1.12-2.19)
**Calcium channel blocker (CCB)**		
[Randomized to Amlodipine in trial: 2189 (27.3)]	1) any CCB	1) no CCB
**Mortality Outcomes:**		
**All-cause mortality**	1.00 (ref)	1.01 (0.90-1.14)
**CVD mortality**	1.00 (ref)	1.05 (0.87-1.25)
CHD mortality	1.00 (ref)	1.09 (0.82-1.43)
Stroke mortality	1.00 (ref)	1.26 (0.76-2.09)
Heart failure mortality	1.00 (ref)	1.03 (0.63-1.66)
Other CVD mortality	1.00 (ref)	0.92 (0.67-1.28)
**Non-CVD Mortality**	1.00 (ref)	0.99 (0.85-1.15)
Cancer	1.00 (ref)	0.95 (0.69-1.31)
Kidney disease	1.00 (ref)	1.08 (0.60-1.95)
Accident/suicide/homicide	1.00 (ref)	0.61 (0.23-1.60)
Other non-CVD disease	1.00 (ref)	1.01 (0.85-1.21)
**Combined fatal and non-fatal**		
**Hospitalized events:**		
CVD	1.00 (ref)	0.91 (0.78-1.05)
CHD	1.00 (ref)	0.88 (0.70-1.11)
Heart failure	1.00 (ref)	0.92 (0.77-1.09)
Stroke	1.00 (ref)	1.03 (0.78-1.35)
Cancer	1.00 (ref)	0.96 (0.75-1.24)
Kidney disease/ESRD	1.00 (ref)	0.91 (0.62-1.35)

Angiotensin-converting enzyme (ACE) inhibitor / Angiotensin II receptor blockers (ARB)		
[Randomized to Lisinopril in trial: 2181 (27.2)]	1) any ACE/ARB	1) no ACE/ARB
**Mortality Outcomes:**		
**All-cause mortality**	1.00 (ref)	1.02 (0.90-1.16)
**CVD mortality**	1.00 (ref)	0.96 (0.79-1.16)
CHD mortality	1.00 (ref)	1.02 (0.76-1.35)
Stroke mortality	1.00 (ref)	0.95 (0.57-1.60)
Heart failure mortality	1.00 (ref)	0.81 (0.44-1.50)
Other CVD mortality	1.00 (ref)	0.92 (0.65-1.32)
**Non-CVD Mortality**	1.00 (ref)	1.08 (0.92-1.27)
Cancer	1.00 (ref)	1.19 (0.86-1.65)
Kidney disease	1.00 (ref)	1.88 (1.07-3.31)
Accident/suicide/homicide	1.00 (ref)	1.15 (0.54-2.46)
Other non-CVD disease	1.00 (ref)	0.96 (0.78-1.18)
**Combined fatal and non-fatal**		
**Hospitalized events:**		
CVD	1.00 (ref)	0.85 (0.72-1.01)
CHD	1.00 (ref)	0.88 (0.69-1.13)
Heart failure	1.00 (ref)	0.75 (0.62-0.92)
Stroke	1.00 (ref)	0.90 (0.66-1.21)
Cancer	1.00 (ref)	1.11 (0.85-1.45)
Kidney disease/ESRD	1.00 (ref)	1.66 (1.12-2.44)

*hazard did not converge.

Baseline covariates in the adjusted hazard ratio models include: age (yrs), race (Black/Non-Black), gender, education (yrs), current smoking status, BMI, entry criterion HDL<35, LDL, GFR, history of diabetes, antihypertensive treatment, on pravastatin, SBP; In-trial covariate included: final SBP.

Table S7b. Adjusted hazard ratio (95% CI) of morbidity and mortality by the end of post-trial (12-31-2017) period by post-trial drug uses

**Total Participants: N=8007**	1) Diuretic only (N=463 [5.8%])	2) CCB only (N=685 [8.6%])	3) ACE/ARB only (N=1,444 [18.0%])	4) 2 or 3 of Diuretic, CCB, or ACE/ARB (N=4,311 [53.8%])	5) No Drugs (N=1,104 [13.8%])
**Mortality Outcomes:**					
**All-cause mortality**	1.00 (ref)	1.20 (1.03-1.41)	1.27 (1.10-1.46)	1.06 (0.93-1.21)	1.22 (1.06-1.42)
**CVD mortality**	1.00 (ref)	1.16 (0.91-1.49)	1.30 (1.04-1.62)	1.05 (0.86-1.29)	1.17 (0.94-1.47)
CHD mortality	1.00 (ref)	1.36 (0.92-2.01)	1.41 (0.99-2.01)	1.24 (0.90-1.72)	1.46 (1.02-2.09)
Stroke mortality	1.00 (ref)	0.93 (0.48-1.80)	1.14 (0.64-2.03)	0.93 (0.56-1.55)	1.03 (0.57-1.87)
Heart failure mortality	1.00 (ref)	0.60 (0.27-1.30)	0.88 (0.47-1.64)	0.92 (0.53-1.59)	0.84 (0.44-1.59)
Other CVD mortality	1.00 (ref)	1.27 (0.83-1.94)	1.40 (0.96-2.06)	0.95 (0.67-1.35)	1.06 (0.71-1.59)
**Non-CVD Mortality**	1.00 (ref)	1.22 (0.99-1.50)	1.24 (1.02-1.49)	1.06 (0.89-1.26)	1.25 (1.03-1.52)
Cancer	1.00 (ref)	1.08 (0.70-1.67)	1.22 (0.83-1.78)	1.01 (0.72-1.43)	1.17 (0.79-1.73)
Kidney disease	1.00 (ref)	2.81 (1.13-6.95)	1.56 (0.63-3.89)	1.46 (0.63-3.37)	2.05 (0.83-5.09)
Accident/suicide/homicide	1.00 (ref)	0.87 (0.28-2.66)	1.14 (0.44-2.94)	0.90 (0.38-2.14)	1.30 (0.50-3.38)
Other non-CVD disease	1.00 (ref)	1.19 (0.92-1.53)	1.23 (0.98-1.55)	1.06 (0.86-1.31)	1.22 (0.97-1.55)
**Combined fatal and non-fatal**					
**Hospitalized events:**					
CHD	1.00 (ref)	1.38 (1.00-1.91)	1.36 (1.02-1.83)	1.25 (0.95-1.63)	1.29 (0.95-1.74)
CVD	1.00 (ref)	1.37 (1.10-1.70)	1.50 (1.23-1.83)	1.30 (1.09-1.56)	1.29 (1.05-1.59)
Heart failure	1.00 (ref)	1.54 (1.18-2.00)	1.71 (1.34-2.18)	1.47 (1.17-1.83)	1.39 (1.08-1.79)
Stroke	1.00 (ref)	1.15 (0.76-1.72)	1.62 (1.13-2.32)	1.27 (0.91-1.77)	1.30 (0.89-1.90)
Cancer	1.00 (ref)	1.03 (0.73-1.44)	1.08 (0.80-1.45)	0.91 (0.69-1.18)	1.06 (0.78-1.43)
Kidney disease/ESRD	1.00 (ref)	4.31 (2.11-8.78)	2.26 (1.10-4.64)	2.15 (1.10-4.21)	3.19 (1.56-6.53)

*incalculable
